# Ultrastable Au nanoparticles on titania through an encapsulation strategy under oxidative atmosphere

**DOI:** 10.1038/s41467-019-13755-5

**Published:** 2019-12-19

**Authors:** Shaofeng Liu, Wei Xu, Yiming Niu, Bingsen Zhang, Lirong Zheng, Wei Liu, Lin Li, Junhu Wang

**Affiliations:** 1 State Key Laboratory of Catalysis, Dalian Institute of Chemical Physics, Chinese Academy of Sciences, Dalian, 116023 China; 2 Mössbauer Effect Data Center, Dalian Institute of Chemical Physics, Chinese Academy of Sciences, Dalian, 116023 China; 3 University of Chinese Academy of Sciences, Beijing, 100049 China; 4 Institute of High Energy Physics, Chinese Academy of Sciences, Beijing, 100049 China; 5 Rome International Center for Materials Science Superstripes, Via dei Sabelli 119A, 00185 Rome, Italy; 6 Shenyang National Laboratory of Materials Science, Institute of Metal Research, Chinese Academy of Sciences, Shenyang, 110016 China

**Keywords:** Catalyst synthesis, Heterogeneous catalysis, Synthesis and processing

## Abstract

Supported gold catalysts play a crucial role in the chemical industry; however, their poor on-stream stability because of the sintering of the gold nanoparticles restricts their practical application. The strong metal-support interaction (SMSI), an important concept in heterogeneous catalysis, may be applied to construct the structure of catalysts and, hence, improve their reactivity and stability. Here we report an ultrastable Au nanocatalyst after calcination at 800 °C, in which Au nanoparticles are encapsulated by a permeable TiO_x_ thin layer induced by melamine under oxidative atmosphere. Owning to the formed TiO_x_ overlayer, the resulting Au catalyst is resistant to sintering and exhibits excellent activity and stability for catalytic CO oxidation. Furthermore, this special strategy can be extended to colloidal Au nanoparticles supported on TiO_2_ and commercial gold catalyst denoted as RR2Ti, providing a universal way to engineer and develop highly stable supported Au catalysts with tunable activity.

## Abstract (web summary)

Sintering-resistant Au nanoparticles are highly desirable due to their low Tammann temperature. Here, the authors report an ultrastable titania-supported Au nanocatalyst through an encapsulation strategy under oxidative atmosphere.

## Introduction

Supported catalysts, dispersed on high specific area, are one of the most important heterogeneous catalysts^1,2^. The primary role of the support is ascribed to increase the dispersion of metal and stabilize the active sites. And the interaction between metal and support named carried effects are also recognized to play a vital role in tuning the activity, selectivity and stability of the catalysts^2–8^. However, much less is known about how meal-support interaction will affect the activity and selectivity of metal-oxide supported catalysts^9,10^.

Gold catalysts have been developed for several industrial processes because of their unique catalytic activity^11–17^. However, poor on-stream stability resulting from Au nanoparticles (NPs) sintering restricts their practical application^18,19^. Up to now, some strategies have been proposed to increase the stability of Au NPs in high-temperature reactions, including fixing the Au NPs inside porous channels, coating the Au NPs with thin shells of carbon or porous oxide and strengthening the meal-support interaction. However, there are still some issues remain to be resolved although great progresses have been achieved^20–35^. For example, Au NPs inside the porous channels are tending to sinter because the pores are poorly controlled^20,21^. As for coating strategy, which may cover the active sites and/or lead diffusion limitations, therefore will reduce the activity although it can stabilize the Au NPs in a degree^22,36^. Comparatively, encapsulating Au NPs with reducible oxide overlayer is valid, achieved by constructing an oxide barrier on the surface of Au NPs, which is known as strong metal-support interaction (SMSI)^27–30^. Generally, strong metal-support interaction (SMSI; now denoted as classical SMSI) was first described by Tauster et al. in the late 1970s to explain the dramatically suppressed CO and H_2_ adsorption on titania-supported platinum group metals after high-temperature reduction treatment^37^. From then on, SMSI has been investigated comprehensively and an agreement has been reached that SMSI emanates from the encapsulation of metals by supports^38,39^. Up to now, SMSI has been exploited to enhance catalytic performance of the catalysts by modifying the electronic and geometric factors of the metal NPs^27,40–48^.

For long ages it has been well-recognized that Au cannot manifest SMSI, which was ascribed to its low work function and surface energy and its low ability to dissociate H_2_ to active the support in the previous investigation^49–52^. Under this assumption, a few successes have been achieved that an oxide overlayer can be formed on gold supported on ZnO, hydroxyapatite and phosphate supports in oxygen atmosphere, contrary to the condition required for classical SMSI^27,53,54^. Later, we demonstrated the existence of classical SMSI between Au and TiO_2_ and the encapsulation of Au by TiO_*x*_ overlayer was also observed^28^. Recently, Xiao et al. constructed a TiO_*x*_ overlayer on Au NPs via wet-chemical strategy in a relative mild condition without reduction pretreatment^30^. More excitingly, Christopher et al. proposed that the adsorbates HCO_*x*_ on TiO_2_- and Nb_2_O_5_-supported Rh catalysts could induce the encapsulation of Rh NPs by supports in CO_2_–H_2_ environment at temperatures of 150–300 °C, denoted as adsorbate-mediated SMSI (A-SASI)^46^.

Herein, we demonstrate that Au NPs can be encapsulated by a permeable TiO_x_ overlayer under oxidative atmosphere induced by melamine, the reverse of condition required for classical SMSI between Au and TiO_2_^28^. The key to construct the overlayer is the application of melamine and pretreatment in nitrogen gas, followed by calcination at 800 °C in air atmosphere, which strengthen the interaction between Au and TiO_2_. Owning to the formation of the overlayer, the resultant catalyst is sintering-resistant with high activity after calcination at 800 °C as well as excellent durability in a simulated practical testing, implying the potential for practical application. More importantly, this special strategy can be extended to colloidal Au NPs supported on TiO_2_ and commercial gold catalyst denoted as RR2Ti, which enables to rationally devise and develop highly stable supported Au catalysts with controllable activity.

## Results

### Physical and chemical nature of the encapsulation layer

**Fig. 1:**
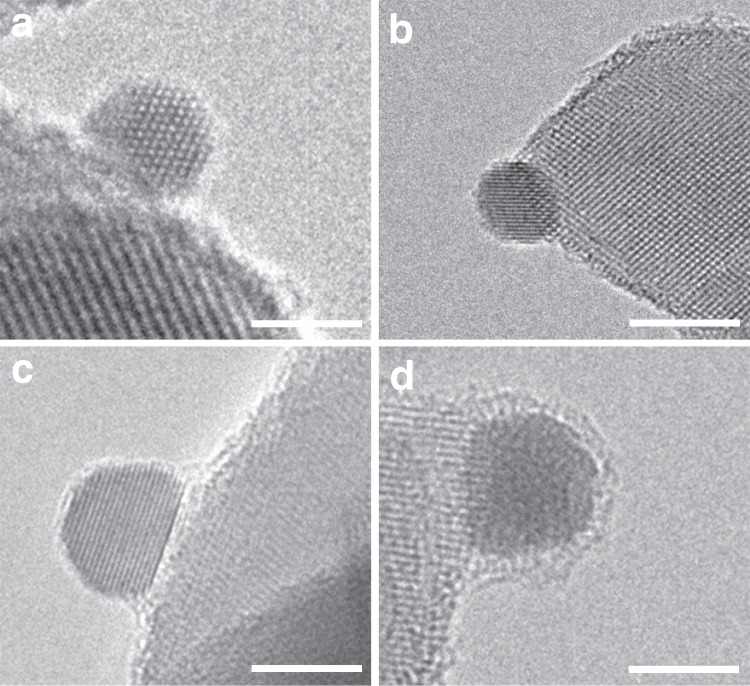
HRTEM analysis. **a** HRTEM image of Au/TiO_2_. **b** HRTEM image of Au/TiO_2_@M. **c** HRTEM image of Au/TiO_2_@M-N. **d** HRTEM image of Au/TiO_2_@M-N-800. The scale bar corresponds to 5 nm. Additional results for these samples can be found in Supplementary Figs. 1–3.

The catalysts were prepared with deposition-precipitation (DP) method and followed by modification with melamine and pretreatment at 600 °C in N_2_ atmosphere and further calcination at 800 °C in air. High-resolution transmission electron microscopy (HRTEM) (Fig. 1) showed the morphology change of Au NPs during synthesis. For the as-prepared Au/TiO_2_, the average particle size of gold was about 3.5 nm after pretreatment at 250 °C in air (Fig. 1a and Supplementary Fig. 1a). After modification with melamine (denoted as Au/TiO_2_@M), the particle size increased to 3.6 nm. However, no melamine was observed on the surface of Au NPs, which was different from the previous work (Fig. 1b and Supplementary Fig. 1b)^23^. When calcination at 600 °C in N_2_ atmosphere for 3 h (denoted as Au/TiO_2_@M-N), the change in the interface was observed, suggesting the variation of the interaction between Au NPs and support (Fig. 1c). Meanwhile, the Au NPs size increased from 3.6 to 6.7 nm (Supplementary Fig. 1c). Surprisingly, after being subjected to calcination at 800 °C in air (denoted as Au/TiO_2_@M-N-800) it was found that Au NPs were covered with an overlayer, which should be from TiO_2_ as melamine would decomposed completely in this condition (Fig. 1d and Supplementary Fig. 2). Meanwhile, the Au NPs size was only about 7.5 nm, indicating a robust sintering-resistant Au nanocatalyst (Supplementary Fig. 1d). It is worth noting that the smaller Au NPs can also be encapsulated similarly to that of the larger ones (Supplementary Fig. 3). However, it is difficult to observe the morphology and encapsulation of gold when the Au NPs size is less than 2 nm. And it was calculated that more than 90% of Au NPs was encapsulated based on the particle size distribution. Comparatively, for the Au/TiO_2_ catalyst without melamine modification after pretreatment at 800 °C (denoted as Au/TiO_2_-800), the catalyst sintered seriously with a particle size about 32.6 nm and no cover layer was observed (Supplementary Fig. 4).

The above results were further examined by X-ray diffraction (XRD) patterns. As pictured in Supplementary Fig. 5, there was no diffraction peaks ascribed to Au NPs for Au/TiO_2_ and Au/TiO_2_@M, suggesting that Au NPs were dispersed uniformly, in agreement with TEM results. While after calcination, diffraction peaks of Au (111) at 38.2° and Au (200) at 44° were observed for Au/TiO_2_@M-N, Au/TiO_2_@M-N-800 and Au/TiO_2_-800, especially for the last two. Furthermore, the crystal structure of TiO_2_ was also analyzed (Supplementary Table 1). And anatase was identified as the primary phase for Au/TiO_2_, Au/TiO_2_@M and Au/TiO_2_@M-N. After calcination at 800 °C, anatase transformed to rutile completely in Au/TiO_2_-800. However, for Au/TiO_2_@M-N-800 sample 12% anatase still existed, implying the phase conversion from anatase to rutile was delayed in a degree. The inhibition may be ascribed to the enhanced interaction between Au and support, in line with the recent reports^23^.

**Fig. 2:**
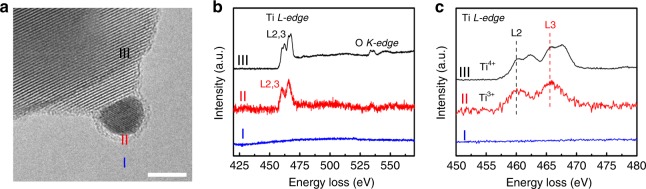
Electron Energy Loss Spectroscopy analysis. **a** HRTEM image of Au/TiO_2_@M-N-800. **b** and **c** Fitted EELS spectra from 450 to 480 eV. The spectra were background-subtracted. The scale bar corresponds to 5 nm. Additional EELS results can be found in Supplementary Figs. 6–8.

To identify the chemical composition of the cover layer in Au/TiO_2_@M-N-800, the sample was probed by electron energy loss spectroscopy (EELS). Figure 2b and Supplementary Fig. 6b showed the obvious Ti L-edge signals in the amorphous overlayer, demonstrating the encapsulation by Ti-containing coating. While no C or N signal was observed in the overlayer region, proving the composition of overlayer was pure TiO_*x*_ (Supplementary Figs. 7, 8). (Note: The signals of C and N in EELS are located at 280 and 400 eV, respectively). On the other hand, N 1*s* XPS spectra in Au/TiO_2_@M definitely demonstrated that melamine was adsorbed on the Au/TiO_2_ (Supplementary Fig. 9). And a decrease of N 1*s* XPS intensity in Au/TiO_2_@M-N may be ascribed to the carbonization of melamine and no N 1*s* XPS signal was observed in Au/TiO_2_@M-N-800 which demonstrated melamine had decomposed completely. The EELS results also revealed that Ti species on the overlayer existed as Ti^3+^ oxidation state (region II) while those on the support were Ti^4+^ oxidation state (region III), respectively, in consistent with the previous reports (Fig. 2c and Supplementary Fig. 6c)^28,46,55^. However, no reduced Ti^3+^ species were observed in the Ti 2*p* XPS spectra (Supplementary Fig. 10), in contrast to the case of classical SMSI, may be due to the smaller number of TiO_*x*_ species^51,56^. The EELS analysis of Au/TiO_2_@M-N-800 catalyst definitely demonstrated the existence and the composition of the overlayer on Au NPs. To the best of our knowledge, this phenomenon has never been reported before as it was well-recognized that Au NPs can only be encapsulated by TiO_x_ in reductive condition at high temperature (classical SMSI), inconsistent with the condition in this work^28,37–39^.

### Changes of Au structure during treatments

**Fig. 3:**
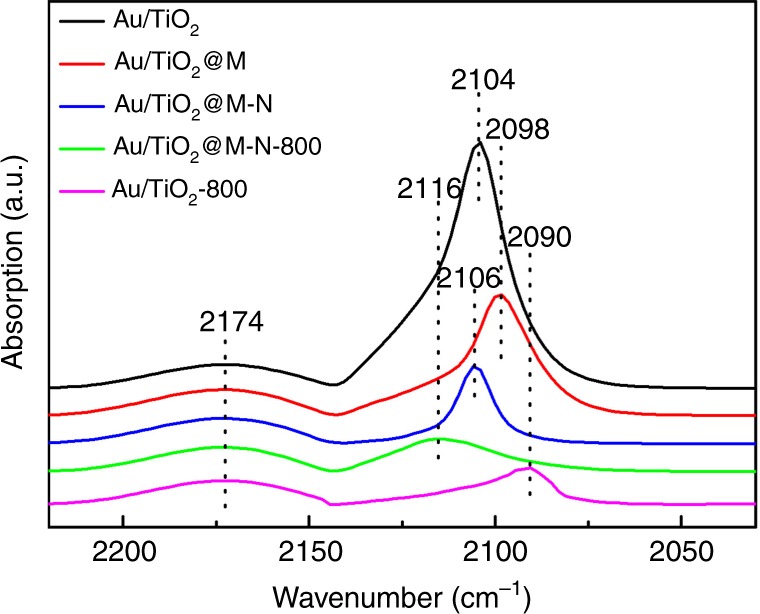
DRIFTS analysis. In situ DRIFT spectra of CO adsorption on Au/TiO_2_, Au/TiO_2_@M, Au/TiO_2_@M-N, Au/TiO_2_@M-N-800, and Au/TiO_2_-800.

To determine if there is adsorption change and/or electron transfer between gold and support, in situ diffuse reflectance infrared Fourier transform spectroscopy (DRIFTS) measurements of CO adsorption were examined. As shown in Fig. 3, two bands were detected on Au/TiO_2_ at 2174 and 2104 cm^−1^, respectively. The former was ascribed to gaseous CO as the peak decreased rapidly and disappeared totally under purge with He (Supplementary Fig. 11a, b). The latter was due to CO adsorbed on metallic Au NPs^57^. When modification with melamine, CO absorption band red-shifted to 2098 cm^−1^ assigned to Au^δ−^ and accompanied by a decrease in intensity. The red-shift of CO adsorption may be due to the electron transfer from melamine to Au and therefore the adsorption intensity decreased, as it was difficult for CO to adsorb on Au^δ−^ and there was no change in the structure morphology of Au NPs^28^. However, after pretreatment at 600 °C in N_2_ flow, 2098 cm^−1^ band was blue-shifted to 2106 cm^−1^ and accompanied by a decrease in intensity. The blue-shift of CO adsorption band may be attributed to electron withdrawing from Au NPs to melamine due to the carbonization of melamine. Furthermore, the decrease of the intensity may be ascribed to the particle size increase. After being subjected to calcination at 800 °C, 2106 cm^−1^ adsorption band blue-shifted to 2116 cm^−1^, suggesting much more positive charge on the surface Au species, which may be attributed to the electron transfer from Au NPs to TiO_2_ support as melamine had decomposed completely at this high temperature. It is noteworthy that 2116 cm^−1^ is not the band of gas-phase CO as it still existed under purge with He while gaseous CO band (2174 cm^−1^) decreased rapidly after 30 s and disappeared completely within 2 min (Supplementary Fig. 11c, d), suggesting that this band is the peak of CO adsorption on Au^δ+^ as CO-Au^δ+^ is quite stable^53,58^. The intensity decrease can be ascribed to the loss of CO adsorption sites resulting from the encapsulation of Au NPs by TiO_*x*_ instead of sintering of the Au NPs as the mean size of Au NPs only increased from 6.7 to 7.5 nm, in good consistent with high-resolution transmission electron microscopy (HRTEM) results. Comparatively, the adsorption band at 2090 cm^−1^ and loss CO adsorption were observed for Au/TiO_2_−800, which was ascribed to the serious sintering of Au NPs leading to the decrease of adsorption sites, consistent with HRTEM results. The red-shift was due to the decrease of the positively charged Au species, in accordance with previous report^27^. While no obvious changes in the binding energy of Au 4*f* were observed, suggesting that melamine treatment and subsequent calcinations did not affect the oxidation state of the surface Au significantly (Supplementary Fig. 12).

**Fig. 4:**
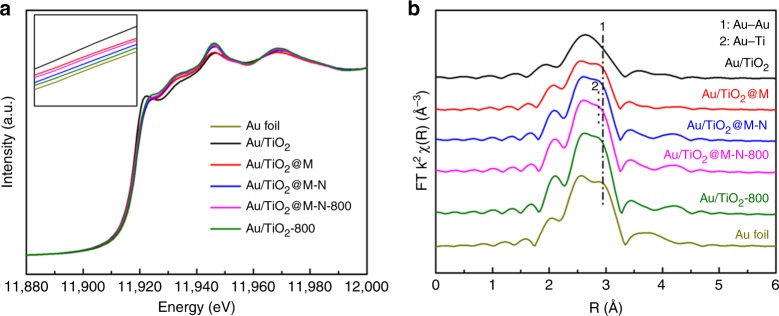
X-ray absorption spectroscopy analysis. **a** Normalized XANES spectra at the Au L_III_-edge of Au foil, Au/TiO_2_, Au/TiO_2_@M, Au/TiO_2_@M-N, Au/TiO_2_@M-N-800, Au/TiO_2_-800. **b** Fourier transform of *k*^3^-weighted EXAFS spectra of Au foil, Au/TiO_2_, Au/TiO_2_@M, Au/TiO_2_@M-N, Au/TiO_2_@M-N-800, and Au/TiO_2_-800.

To further investigate the interaction between Au NPs and TiO_2_, Au L_III_-edge XAFS spectra were collected on a series of samples to follow the coordination environment and valence state of the gold. As displayed in Fig. 4a, we showed the normalized Au L_III_-edge XANES spectra recorded on a series of Au/TiO_2_ samples. The white line intensity was much stronger for Au/TiO_2_ and Au/TiO_2_@M than other samples, which was ascribed to the presence Au^3+^^59^. Moreover, the white line intensity of Au/TiO_2_@M-N-800 was stronger than Au/TiO_2_@M-N and Au/TiO_2_-800, indicating much more positive charge on the gold. In the Fourier transforms of *k*^3^-weighted EXAFS oscillations (Fig. 4b), a two-shells fitting was performed. In the first shell, the coordination number of Au-Au1 increased from 5.9 to 8.4 and the average bond length of Au-Au1 increased from 2.82 to 2.85 Å when the pretreatment temperature increased from 250 to 800 °C. While for Au/TiO_2_-800 the coordination number and the average bond length of Au-Au1 were 9.8 and 2.86 Å, respectively, which was much closer to that of Au foil that suggested the d orbital of Au was almost entirely occupied, in good accordance with the XANES results. Moreover, it was found that including additional Au–O shells would lower the quality of fitting results, which may be ascribed to the growth of the Au NPs size. Furthermore, a two-shell analysis of the narrow window region data of Au/TiO_2_@M-N-800 using Au–Au and Au–Ti scattering parameters gave a good fitting result. The best fitting was obtained with Au-Au1 bond distance of 2.85 Å (*N* = 8.4) and Au–Ti bond distance of 2.79 Å (*N* = 5.6). The expected Au–Ti distance of 2.79 Å was similar to that of AuTi alloy phase^60^. The fitting results were summarized in Supplementary Table 2 and the *k*-space curves were depicted in Supplementary Fig. 13. And the previous studies on Pt/TiO_2_ and Rh/TiO_2_ catalysts in the SMSI state provided direct evidence for Pt–Ti bond and Rh–Ti bond with a distance of 2.76 and 2.56 Å, respectively^61,62^. Therefore, there was no doubt that the encapsulation state leaded to a structural reorganization of the TiO_2_ support in the neighborhood of the Au NPs. And this reorganization of the support only generated where there was Au NPs and encapsulation occurred. Otherwise, no reorganization would occur.

### Catalytic performance in a series of reactions

**Fig. 5:**
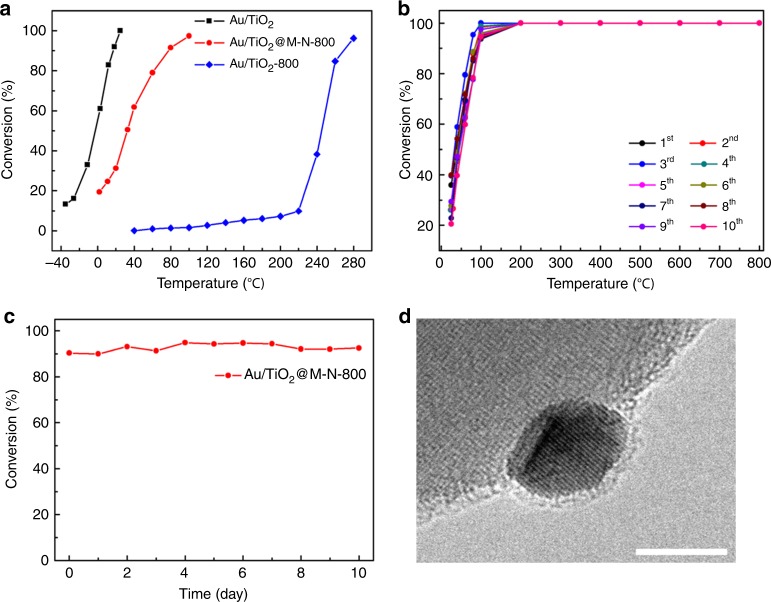
Evaluation of Au/TiO_2_ nanocatalysts in CO oxidation and simulated CO emission control reaction. **a** CO oxidation curves of Au/TiO_2_, Au/TiO_2_@M-N-800 and Au/TiO_2_-800 with a feed gas comprising 1 vol% CO/ 1 vol% O_2_/ 98 vol% He at 33.3 mL min^−1^. **b** Conversion of CO from 0 to 800 °C with 1st-10th cycles on Au/TiO_2_@M-N-800 catalyst for ten ignition-extinction cycles with a feed gas comprising 1 vol% CO/1 vol% O_2_/98 vol% He at 33.3 mL min^−1^. **c** Long-term simulated CO emission control reaction at 400 °C on the Au/TiO_2_@M-N-800 with space velocity of 220 L h^−1^ g_cat_^−1^. Reaction gas composition: 1.6 vol% CO, 1 vol% O_2_, 0.01 vol% propene, 0.00852 vol% toluene, 10 vol% water and balanced with He. **d** HRTEM image of Au/TiO_2_@M-N-800 after simulated CO emission control reaction at 400 °C for 10 days. The scale bar corresponds to 5 nm.

CO oxidation was performed as a typical probe reaction due to its great importance in fundamental study, especially its size-dependent behavior^63^. As shown in Fig. 5a, Au/TiO_2_ revealed the highest activity with a T50 (the temperature 50% conversion is acquired) of −2.1 °C. After calcination at 800 °C, Au/TiO_2_@M-N-800 exhibited a much higher activity compared with Au/TiO_2_-800, explicitly displaying the strong effect of particle size on catalytic performance. According to the active curve, the T50 was determined to be 32 °C for Au/TiO_2_@M-N-800. While for Au/TiO_2_-800 the activity was negligible below 220 °C with a T50 of 245 °C. On the basis of TEM and DRIFTS characterization results, the poor activity of Au/TiO_2_-800 was attributed to the serious sintering of Au NPs. However, for Au/TiO_2_@M-N-800 it was due to the encapsulation of Au by TiO_x_ overlayer leading to the partial decrease of accessible active sites. To investigate the kinetic study of the above catalysts, the specific reaction rates and turn over frequency (TOF) were determined at 25 °C. For Au/TiO_2_ catalyst, the reaction rate and TOF were 0.536 mol_CO_ h^−1^ g_Au_^−1^ and 0.12 s^−1^, respectively, in good accordance with the above results. While for Au/TiO_2_@M-N-800, the reaction rate was induced by a 3-fold decrease from 0.536 to 0.186 mol_CO_ h^−1^ g_Au_^−1^. As for Au/TiO_2_-800, the sintering of Au NPs decreased the reaction rate by about 90-fold from 0.536 to 0.006 mol_CO_ h^−1^ g_Au_^−1^.

Furthermore, the activity of the Au/TiO_2_@M-N-800 was also compared with that of Au/TiO_2_ catalysts in classical SMSI state before and after calcination. It was showed that the T50 of Au/TiO_2_-H500 and Au/TiO_2_-H500-O400 were −19.5 and 16.5 °C and the corresponding particle size were about 3.2 and 4.2 nm, respectively (Supplementary Figs. 14–16). HRTEM results showed that Au NPs were encapsulated with a TiO_*x*_ overlayer in Au/TiO_2_-H500, which retreated after further calcination under oxidation condition, consistent with our previous report^28^. It should be noted that the TiO_*x*_ overlayer in classical SMSI was much thinner than the overlayer induced by melamine in this work. After calcination at 800 °C in air for 3 h, the T50 of Au/TiO_2_-H500–800 and Au/TiO_2_-H500-O400–800 were 191 and 168 °C and the particle size were 28.6 and 27.2 nm, respectively, in which no overlayer was observed and Au NPs sintered seriously (Supplementary Figs. 17, 18). Therefore, the overlayer formed in the reduction condition retreated under oxidation condition (classical SMSI) may account for the sintering of Au in Au/TiO_2_-H500–800, leading to invalid effect on the catalytic and stability property of the underlying metal. And the T50 of Au/TiO_2_@M-N-800 was much lower than that of Au/TiO_2_-H500–800, exhibiting the excellent sintering-resistant ability and superiority of Au/TiO_2_@M-N-800 compared with Au/TiO_2_-H500–800. For comparison, the specific rate and/or TOF of the above the catalysts and some reported catalysts are listed in Supplementary Table 3. To the best of our knowledge, it is the first time to report that Au NPs can be encapsulated by titania under oxidative atmosphere at high temperature, in contrast with condition required for classical SMSI. Although the resultant Au/TiO_2_@M-N-800 does not outperform the state-of-the-art, it is still comparable to the Au/TiO_2_-HAP-800 and Au/TiO_2_-SiO_2_-800 catalysts, which are the most excellent sintering-resistance catalyst up to now.

The reaction stability of Au/TiO_2_@M-N-800 catalyst was tested with CO oxidation at 400 °C with a high space velocity of 500 L h^−1^ g_cat_^−1^. As shown in Supplementary Fig. 19, the conversion increased from 79.1 to 90.1% during 100 h test instead of deactivation, demonstrating the excellent sintering-resistance and stability. HRTEM images of the used Au/TiO_2_@M-N-800 catalyst proved that the overlayer was still covering Au NPs, explaining the distinguished stability (Supplementary Fig. 20c, d). And the increase of the activity during the reaction may be due to much more active sites were accessible, as the overlayer on the used catalyst was not so dense compared with the fresh catalyst (Supplementary Fig. 20e, f). The recycle performance of Au/TiO_2_@M-N-800 was also examined by successive 10 ignition-extinction cycles up to 800 °C. Figure 5b displayed that the catalyst exhibited a slight decrease of the activity after 10th cycles. HRTEM images of the used Au/TiO_2_@M-N-800 catalyst demonstrated that TiO_x_ overlayer still existed on the Au NPs which afforded the sinter-resistant Au nanocatalyst (Supplementary Fig. 21). And it has been acknowledged that water would expedite the sintering of Au NPs. Therefore, water-gas shift (WGS) reaction was performed on Au/TiO_2_@M-N-800 catalyst at 500 °C with space velocity of 18 L h^−1^ g_cat_^−1^. As expected, the conversion almost kept at 45 % during the 90 h test, further certifying the excellent stability of Au/TiO_2_@M-N-800 catalyst (Supplementary Fig. 22). TEM image of the used Au/TiO_2_@M-N-800 catalyst exhibited that the particle size of Au NPs remained unchanged. HRTEM images demonstrated that the Au NPs were still encapsulated by TiO_*x*_ overlayer after reaction, leading to ultrastable Au NPs (Supplementary Fig. 23). To further evaluate the stability of Au/TiO_2_@M-N-800 in presence of water and other gases, simulated CO emission control was performed at 400 °C with the feed mixture gas containing 1.6 vol% CO, 1 vol% O_2_, 0.01 vol% propene, 0.00852 vol% toluene, 10 vol% water and balanced with He. Figure 5c showed that CO conversion of Au/TiO_2_@M-N-800 increased from 90.3 % to 92.5 % during the test for 10 days, exhibiting an excellent durability. TEM image of the used Au/TiO_2_@M-N-800 catalyst showed that the mean size of Au NPs was 7.3 nm, almost similar with the fresh catalyst (Supplementary Fig. 24a, b). HRTEM images (Fig. 5d and Supplementary Fig. 24c) revealed the still existence of the TiO_x_ overlayer on the Au NPs after reaction for 10 days, which meant that water and other gases in the reaction almost had no influence on the TiO_x_ overlayer. And the increase of the activity during the reaction may be due to much more accessible active sites (Supplementary Fig. 24d–f). However, for the catalyst without the encapsulation, the reactivity decreased continuously because of the aggregation of Au NPs^27,30^. Therefore, the encapsulation of Au by TiO_*x*_ cover layer plays a crucial role in influencing the catalytic performances.

### Relating encapsulation induced by melamine to SMSI behavior

**Fig. 6:**
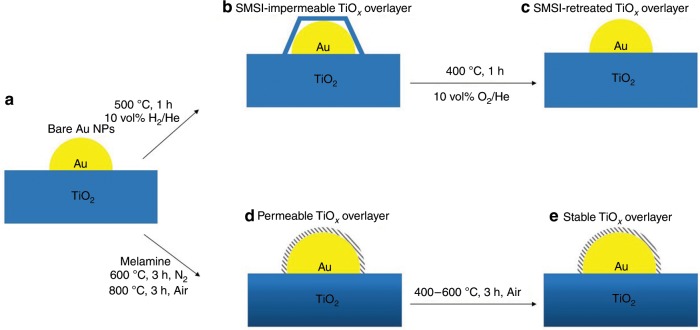
SMSI and melamine-induced TiO_x_ overlayer structure and behavior. **a** Bare Au nanoparticles on TiO_2_. **b** Au/TiO_2_ catalyst that forms an impermeable SMSI TiO_*x*_ overlayer after treatment with 10 vol% H_2_/He at 500 °C. **c** SMSI TiO_*x*_ overlayer retreats when exposed to oxidation condition at 400 °C which is almost similar with that in **a**. **d** Melamine-modified catalyst that forms a permeable TiO_*x*_ overlayer after treatment with N_2_ at high temperature (600 °C) followed by treatment with air at 800 °C. **e** Stable melamine-induced TiO_*x*_ overlayer under air condition modifies the Au NPs catalytic bahavior.

In above, we have demonstrated the encapsulation of Au NPs by TiO_*x*_ overlayer under oxidative condition induced by melamine while high temperature reduction pretreatment is prerequisite for the encapsulation of Au NPs by TiO_*x*_ in classical SMSI. Furthermore, this kind of encapsulation may not belong to classical SMSI as the mean size of the Au NPs increases from 3.5 to 7.5 nm while the mean diameter of Au NPs remains almost unchanged in classical SMSI. Therefore, the formation mechanisms of these two kinds of overlayer are different. The formation process of classical SMSI overlayer and melamine-induced TiO_*x*_ overlayer can be illustrated by Fig. 6. To investigate whether the overlayer will recede, Au/TiO_2_@M-N-800 sample was further calcined in air atmosphere at 400 °C, 500 °C and 600 °C for 3 h, respectively. HRTEM results showed that Au NPs were still encapsulated by TiO_*x*_ overlayer, which was different from classical SMSI in which TiO_*x*_ overlayer will retreat under further heating in oxidation condition (Supplementary Figs. 25–27)^28,37,38,55^. Moreover, the encapsulation TiO_*x*_ overlayer induced by melamine is porous and permeable to reactant and very stable under oxidative reaction condition, which may minimize the reactants diffusion on Au surface and strongly enhance the sintering-resistance and catalytic performance of the Au.

As shown in Fig. 1c and Supplementary Fig. 28, encapsulation did not occur when calcination in N_2_ flow at 600 °C. And no cover layer was observed even calcination in N_2_ at 800 °C (Supplementary Fig. 29), which indicated that encapsulation could not occur only calcination in N_2_ follow. As displayed in Supplementary Figs. 30–31, when Au/TiO_2_@M sample was heated directly in air at 600 or 800 °C without pretreatment in N_2_ atmosphere no overlayer was observed, suggesting the indispensable of pretreatment in N_2_. Supplementary Figs. 32–33 showed that overlayer also could not be formed when calcination in air at 500 or 600 °C after pretreatment in N_2_, which meant higher anneal temperature was requisite. For Au/TiO_2_ catalyst without modification with melamine and pretreatment under similar condition with that of Au/TiO_2_@M-N-800 (denoted as Au/TiO_2_-N-800), no cover layer was observed instead of the sintering of Au NPs, indicating the significant role of melamine in the formation of TiO_x_ overlayer (Supplementary Fig. 34). To identify whether the encapsulation can be extended to other related materials, two catalysts (denoted as Au/anatase@M-N-800 and Au/rutile@M-N-800) were prepared with the same method as Au/TiO_2_@M-N-800. As shown in Supplementary Figs. 35–36, a TiO_x_ overlayer was observed on both catalysts, evidencing a common encapsulation phenomenon between Au and theses two titania supports. Moreover, similar encapsulations can also be obtained on colloidal Au NPs supported on TiO_2_ (denoted as C–Au/TiO_2_@M-N-800) and commercial gold catalyst RR2Ti (denoted as RR2Ti@M-N-800), suggesting the universality of this strategy and providing a widespread way which enables to rationally design sintering-resistant gold catalysts via TiO_*x*_ overlayer encapsulation strategy (Supplementary Figs. 37–38).

It is worth to summarize the mechanism and the effect of adsorbate melamine on the formation of the TiO_*x*_ overlayer and stabilization of Au NPs. The HRTEM and EELS results verify the role of melamine in mediating TiO_*x*_ overlayer formation. On the basis of identification Ti^3+^ in the cover layer by EELS, we hypothesize the plausible mechanism is that electron is transferred from Au NPs to TiO_2_ under the effect of melamine at high annealing temperature under oxidative condition, leading to the reduction of Ti^4+^ to Ti^3+^ and partial positive charge on gold, in consistent with DRIFTS results. Meanwhile, EXAFS fitting results corroborate the presence of Au–Ti bond in the encapsulation state, which may lead to the migration of titania onto Au NPs thermodynamically favorable^46,56^, similar with classical SMSI in which Pt–Ti and Rh–Ti bond are observed after high temperature reduction pretreatment. However, the specific effect of melamine in electron transfer is still unclear and much more work is deserved to be done. And further research is also required to clarify the real driving force for this kind of encapsulation.

## Discussion

In summary, we have presented an ultrastable titania-supported Au nanocatalyst in which Au NPs were encapsulated with a permeable TiO_*x*_ overlayer mediated by melamine under oxidative atmosphere. The indispensable pretreatment condition is anneal in N_2_ flow and further calcination at 800 °C under air condition, in contrast with classical SMSI which occurs under reductive condition. And the overlayer is stable even further calcination under oxidative condition, which is contrary to classical SMSI. Owing to the formation of permeable and stable TiO_*x*_ layer, the designed catalyst is sintering-resistant with high catalytic activity and excellent durability. In addition, this methodology can be extended to colloidal Au catalyst and even commercial RR2Ti catalyst. More importantly, this work paves a new avenue for designing sintering-resistant Au nanocatalysts with high activity via TiO_*x*_ overlayer encapsulation strategy.

## Methods

### Synthesis of Au/TiO_2_

As a typical run, Au/TiO_2_ was synthesized with the DP method. A 40 mL of HAuCl_4_·4H_2_O aqueous solution (6.3 mmol L^−1^) was adjusted to PH 9.0 with 0.1 M NaOH. Then, 1 g of TiO_2_ (Degussa P25) was added to the above solution and the PH was kept to 9.0 for 1 h by adding NaOH. The slurry was heated to 65 °C and magnetically stirred for 1 h. After centrifugation and washed with ultrapure water until free of chloride ions, the resultant solid was dried at 60 °C overnight and calcined at 250 °C for 2 h in air. The actual content of Au was 3.84 wt % determined by inductively coupled plasma-optical emission spectroscopy (ICP-OES).

### Synthesis of C–Au/TiO_2_

C–Au/TiO_2_ was synthesized by colloidal deposition method. 2.6 mL of 0.045 M HAuCl_4_·4H_2_O solution and 3.33 mL of 0.5 wt% PVA (Mw 10,000; 80% hydrolyzed) solution were added to 100 mL ultrapure water. After stirring for 30 min, 6.6 mL NaBH_4_ (0.1 mol L^−1^) solution was rapidly added into the above solution and a dark-brown solution was produced, indicating the formation of gold colloid. 0.5 g of TiO_2_ (Degussa P25) was added under vigorous stirring. After stirred for 6 h, the solid was washed with ultrapure water and dried at 60 °C overnight, followed by heating at 300 °C for 2 h in 10 vol% O_2_/He to remove PVA. The resulting sample was denoted as C-Au/TiO_2_.

### Synthesis of Au/TiO_2_-H500

The Au/TiO_2_-H500 was synthesized by reducing Au/TiO_2_ under 10 vol% H_2_/He at 500 °C for 1 h following a previously reported procedure^28^.

### Synthesis of Au/TiO_2_-H500-800

The Au/TiO_2_-H500-800 was synthesized by directly calcining Au/TiO_2_-H500 at 800 °C in air for 3 h at a heating rate of 2 °C min^−1^.

### Synthesis of Au/TiO_2_-H500-O400

The Au/TiO_2_-H500-O400 was synthesized by reoxidizing Au/TiO_2_-H500 under 10 vol% O_2_/He flow at 400 °C for 1 h following a previously reported procedure^28^.

### Synthesis of Au/TiO_2_-H500-O400–800

The Au/TiO_2_-H500-O400–800 was synthesized by directly calcining Au/TiO_2_-H500-O400 at 800 °C in air for 3 h at a heating rate of 2 °C min^−1^.

### Synthesis of Au/TiO_2_@M-N-800

Typically, 0.15 g as-synthesized Au/TiO_2_ was added to the melamine-containing (1.67 mg mL^−1^) solution (30 mL) and the slurry was stirred at 65 °C for 24 h. After centrifugation and washed with ultrapure water, the product was dried at 60 °C overnight. The obtained sample was denoted as Au/TiO_2_@M and then annealed at 600 °C in N_2_ flow for 3 h at a heating rate of 5 °C min^−1^. The resulting sample was denoted as Au/TiO_2_@M-N. Then the Au/TiO_2_@M-N-800 was synthesized by calcining Au/TiO_2_@M-N at 800 °C in air for 3 h at a heating rate of 2 °C min^−1^.

### Synthesis of Au/TiO_2_@M-T

The Au/TiO_2_@M-T series were synthesized by directly calcining Au/TiO_2_@M in air at different temperature for 3 h at a heating rate of 2 °C min^−1^, where T (600, 800) represents the calcining temperature (°C).

### Synthesis of Au/TiO_2_@M-N800

The Au/TiO_2_@M-N800 was synthesized by calcining Au/TiO_2_@M in N_2_ at 800 °C for 3 h at a heating rate of 5 °C min^−1^.

### Synthesis of Au/TiO_2_@M-N-T

The Au/TiO_2_@M-N-T series were synthesized by calcining Au/TiO_2_@M-N in air at different temperature for 3 h at a heating rate of 2 °C min^−1^, where T (500, 600) represents the calcining temperature (°C).

### Synthesis of Au/TiO_2_@M-N-800-T

The Au/TiO_2_@M-N-800-T series were synthesized by calcining Au/TiO_2_@M-N-800 in air at different temperature for 3 h at a heating rate of 2 °C min^−1^, where T (400, 500, 600) represents the calcining temperature (°C).

### Synthesis of Au/TiO_2_-800

The Au/TiO_2_-800 was synthesized by directly calcining Au/TiO_2_ at 800 °C in air for 3 h at a heating rate of 2 °C min^−1^.

### Synthesis of Au/TiO_2_-N-800

The Au/TiO_2_-N-800 was synthesized by calcining Au/TiO_2_ in N_2_ at 600 °C for 3 h at a heating rate of 5 °C min^−1^, followed by calcining at 800 °C in air for 3 h at a heating rate of 2 °C min^−1^.

### Synthesis of Au/anatase@M-N-800

The synthesis procedure of Au/anatase@M-N-800 is similar to that of Au/TiO_2_@M-N-800 except anatase was sued instead of TiO_2_.

### Synthesis of Au/rutile@M-N-800

The synthesis procedure of Au/rutile@M-N-800 is similar to that of Au/rutile@M-N-800 except rutile was sued instead of TiO_2_.

### Synthesis of C-Au/TiO_2_@M-N-800

The synthesis procedure of C-Au/TiO_2_@M-N-800 is similar to that of Au/TiO_2_@M-N-800 except C-Au/TiO_2_ was sued instead of Au/TiO_2_.

### Synthesis of RR2Ti@M-N-800

The commercial gold catalyst RR2Ti was supplied by Haruta Gold Inc. The synthesis procedure of RR2Ti@M-N-800 is similar to that of Au/TiO_2_@M-N-800 except RR2Ti was sued instead of Au/TiO_2_.

### Characterization

XRD patterns were collected on a PANalytical X’Pert Pro diffractometer with Cu Kα radiation (*λ* = 0.15432 nm). The gold content was determined by inductively coupled plasma-optical emission spectroscopy analysis (Perklin–Elmer 2100 DV). TEM and HRTEM were performed on JEM-2100 electron microscopy (JEOL) with an acceleration voltage of 200 kV. EELS analysis was carried out on an FEI Tecnai G2 F20 microscope equipped with Gatan Imaging Filter (GIF) system operated at 200 kV. The chemical compositions of the covering layer of Au NPs were characterized by directly putting electron beam at the Au NPs in a STEM mode. In situ DRIFTS spectra were recorded on Bruker Equinox 70 spectrometer equipped with MCT detector and operated at a resolution of 4 cm^−1^. Before CO adsorption, the samples were pretreated with He at 120 °C for 1 h and then cooled to room temperature. The background spectrum was collected in following He and then the gas (3 vol% CO/He) was introduced into the reaction cell at a total flow rate of 33.3 mL min^−1^. The spectra were recorded until the peak intensity was steady. The blank test was carried out following the exactly same procedure just without catalyst. X-ray photoelectron spectroscopy measurements (XPS) were collected on Thermo Fisher Scientific ESCALAB 250 spectrometer with monochromatic Al Kα (1486.6 eV) X-ray source. The binding energy (BE) value of C 1 *s* at 284.6 eV was used as the reference. X-ray adsorption fine structure (XAFS) measurements at Au L_III_-edge in fluorescence mode were performed at Beamline 1W1B in Beijing Synchrotron Radiation Facility (BSRF). The electron storage ring was operated at ~200 mA and ~2.2 GeV with a top-up injection mode. A double-crystal Si (111) monochromator was used to scan X-ray energy for X-ray adsorption near edge (XANES) spectra and extended X-ray fine structure (EXAFS) spectra, respectively. The Au foil was used as reference for X-ray energy calibration. Data processing and analysis were performed by Demeter software package. The crystallographic structure of Au, Au_2_O_3_, AuTi were used as models for EXAFS fitting.

### Catalytic activity measurement

CO oxidation was carried out in a fixed-bed reactor under atmospheric pressure. The gaseous mixture of 1 vol% CO + 1 vol% O_2_ and He balance (33.3 mL min^−1^) was passed through the catalysts. The unreacted reactants and products were analyzed online by gas chromatograph equipped with a TCD detector. WGS reaction was performed in the same reactor as was used for CO reaction and a mixture of 2 vol% CO + 10 vol% H_2_O and He balance was used with a space velocity of 18 L h^−1^ g_cat_^−1^. The simulated CO emission control test was carried out in the same reactor and the feed gas was 1.6 vol% CO + 1 vol% O_2_ + 0.01 vol% propene + 0.0087 vol% toluene + 10 vol% water and He balance with a space velocity of 220 L h^−1^ g_cat_^−1^. Specific reaction rates and TOF were evaluated by changing the weight of catalysts from 100 mg to 2 mg to guarantee that CO conversion was below 15%. For each run, the CO conversion was averaged at the steady state and the TOF was calculated according to equation: TOF = r_CO_*M_Au_/D, where r_CO_ is the specific reaction rates.

## Supplementary information

Supplementary Information

Peer Review

## Data Availability

All data are available within the article and its Supplementary Information file are available from the authors upon reasonable request.
