# Supplementary material for: Ultrastable Au nanoparticles on titania through an encapsulation strategy under oxidative atmosphere
Source: Nat Commun. 2019 Dec 19;10:5790. doi: 10.1038/s41467-019-13755-5 (PMC6923380; doi:10.1038/s41467-019-13755-5)
Supplement: Supplementary file 1 — Supplementary Information [file 41467_2019_13755_MOESM1_ESM.pdf]

Supplementary information

Ultrastable Au nanoparticles on titania through an encapsulation strategy under oxidative atmosphere

Liu et al.

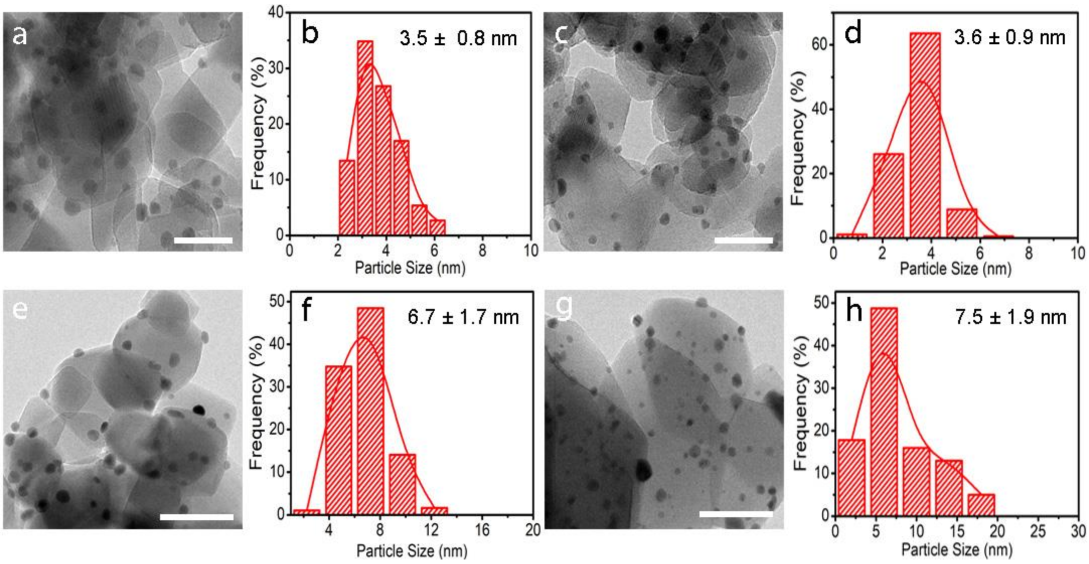

Supplementary Figure 1. TEM images and particle size distribution. a TEM image of Au/TiO_2. **b** Particle size distribution of Au/TiO_2. **c** TEM image of Au/TiO_2@M. **d** Particle size distribution of Au/TiO_2@M. **e** TEM image of Au/TiO_2@M-N. **f** Particle size distribution of Au/TiO_2@M-N. **g** TEM image of Au/TiO_2@M-N-800. **h** Particle size distribution of Au/TiO_2@M-N-800. The scale bar in **a** and **c** corresponds to 20 nm, and in **e** and **g** corresponds to 50 nm.

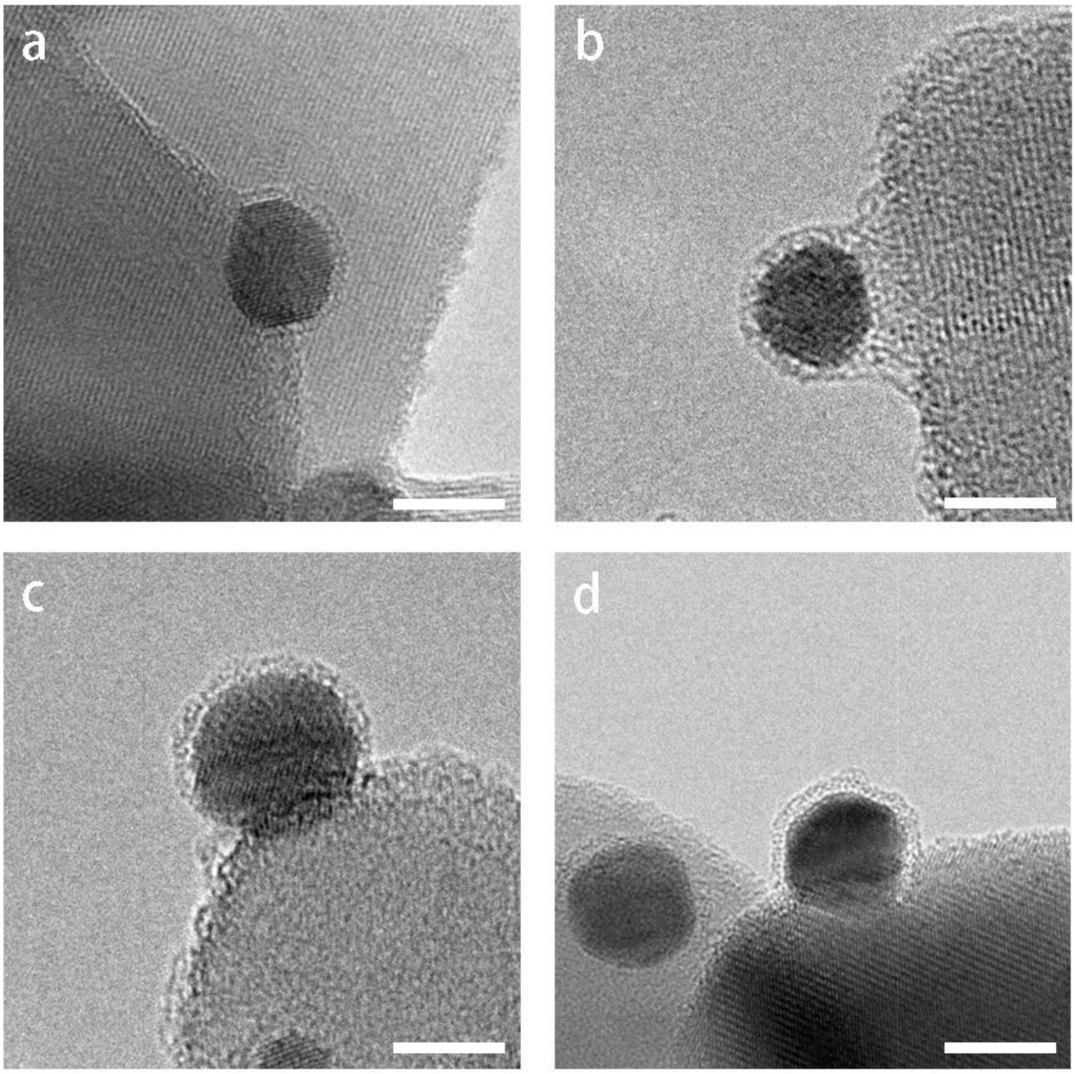

Supplementary Figure 2. HRTEM analysis. a, b, c and **d** HRTEM images of Au/TiO_2@M-N-800, in which all Au NPs were encapsulated by TiO_x overlayer. The scale bars are all 5 nm.

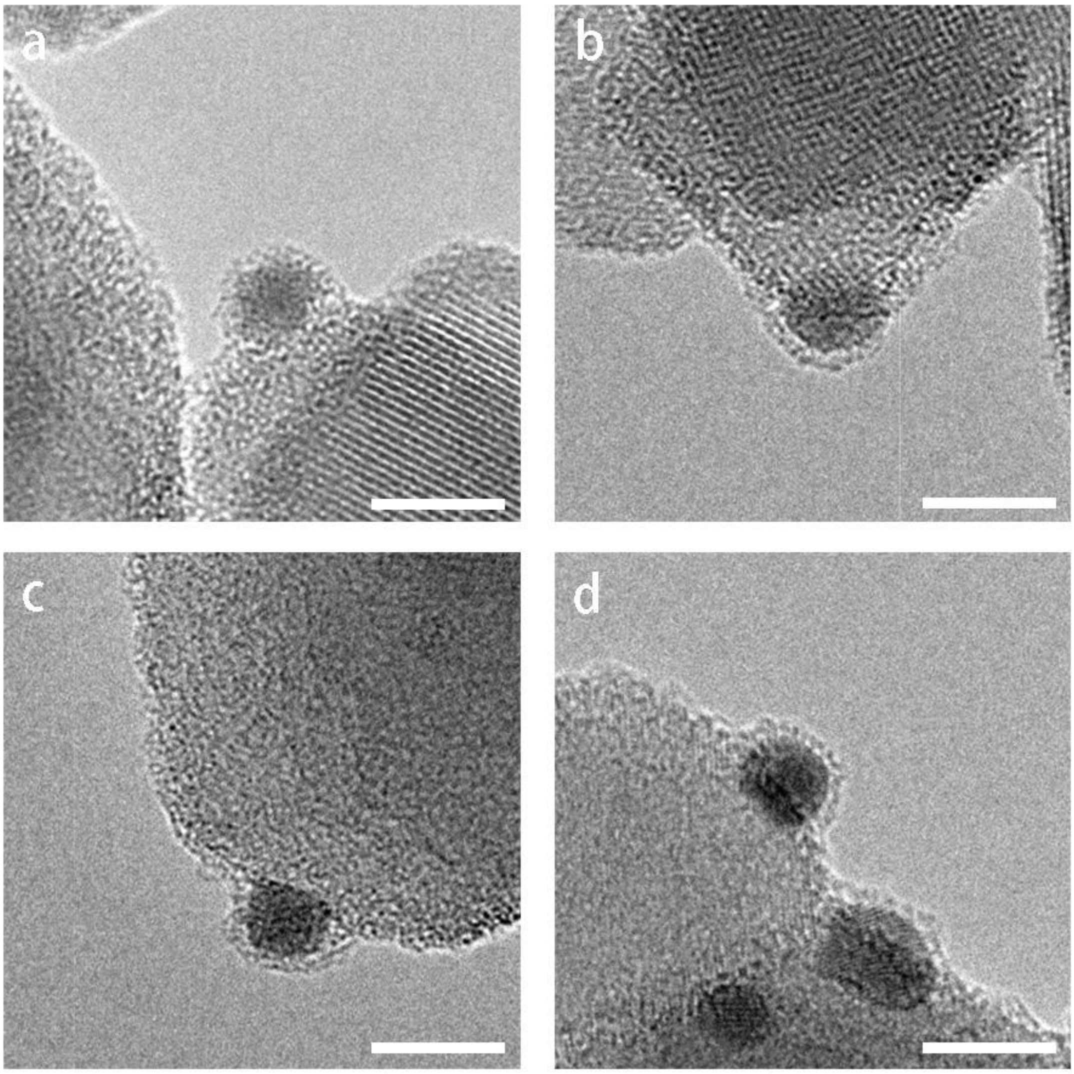

Supplementary Figure 3. HRTEM analysis. a, b, c and **d** HRTEM images of Au/TiO_2@M-N-800, in which all small Au NPs were encapsulated by TiO_x overlayer. The scale bars are all 5 nm.

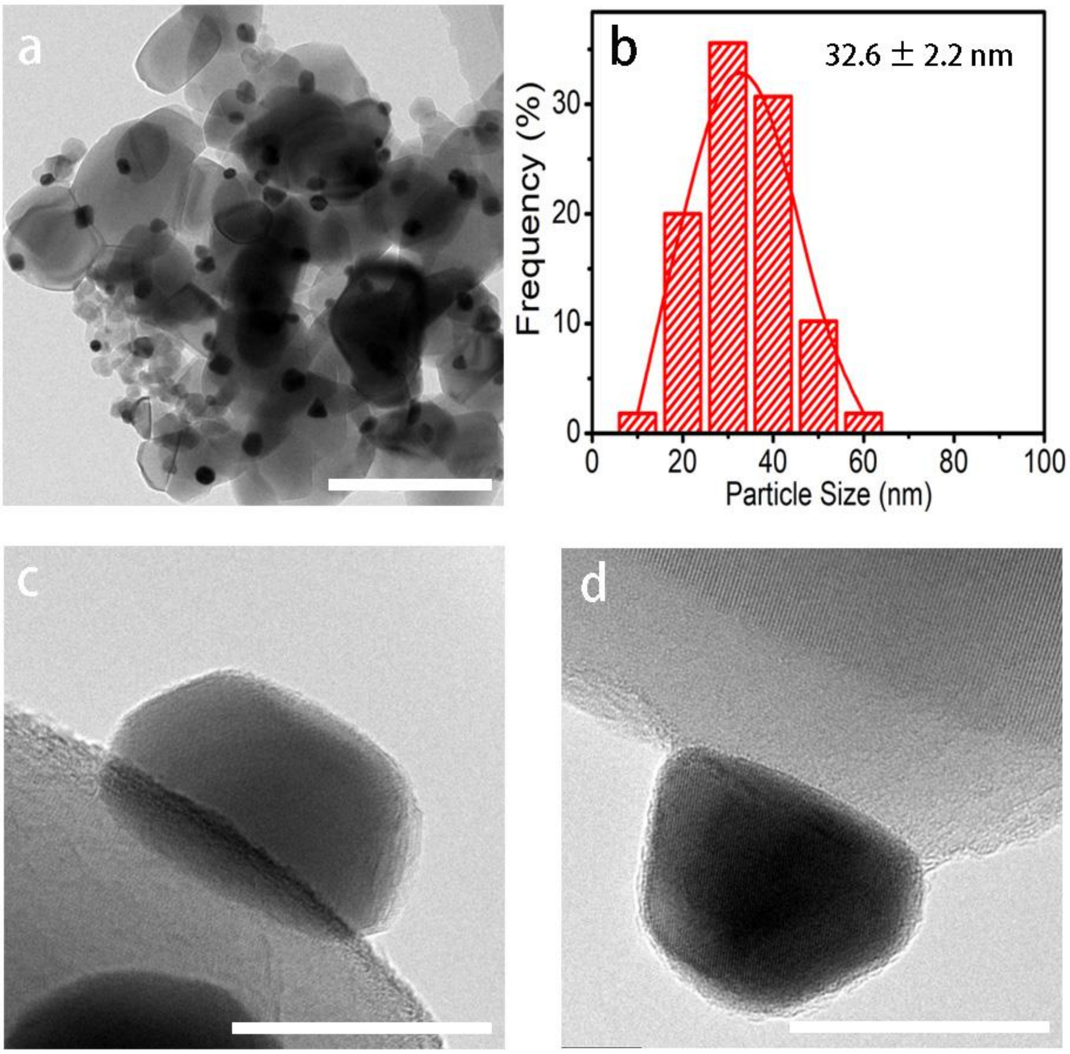

Supplementary Figure 4. Electron microscopy images and particle size distribution of Au/TiO_2-800. a TEM image of Au/TiO_2-800. **b** Particle size distribution of Au/TiO_2-800. **c** and **d** HRTEM images of Au/TiO_2-800, in which Au NPs sintered seriously. The scale bar in **a** corresponds to 200 nm, and in **c** and **d** corresponds to 20 nm.

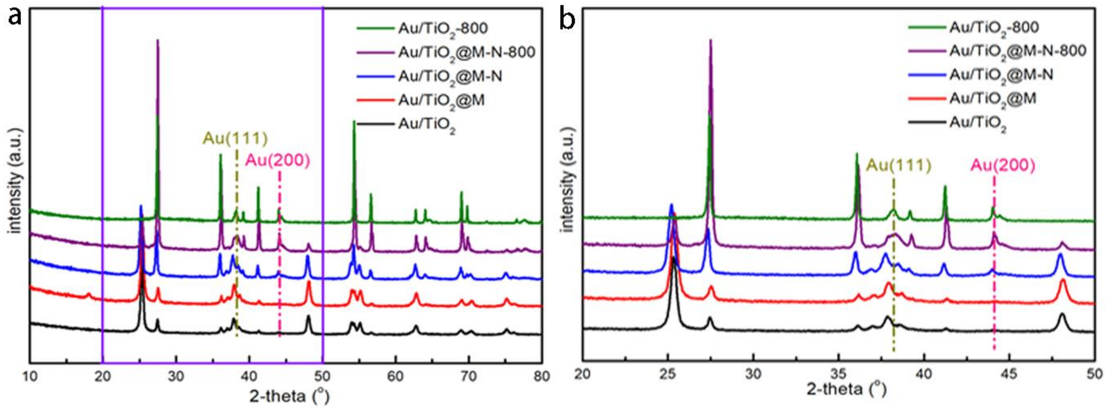

Supplementary Figure 5. XRD characterization. **a** XRD patterns of Au/TiO_2, Au/TiO_2@M, Au/TiO_2@M-N, Au/TiO_2@M-N-800 and Au/TiO_2-800. **b** The enlargement of the box in **a**.

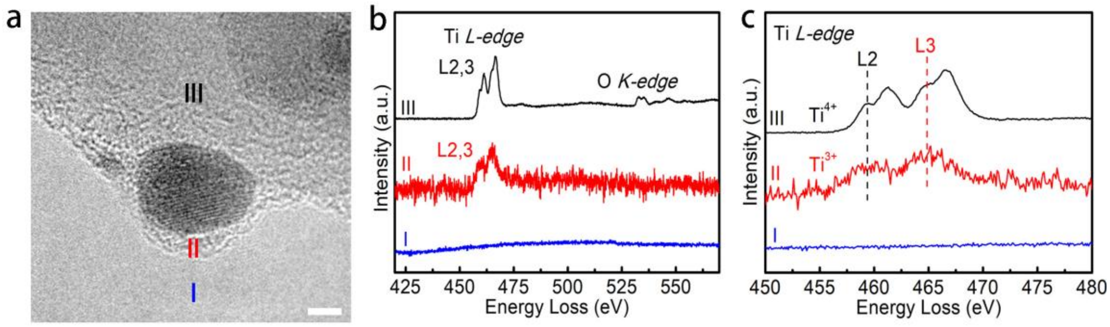

Supplementary Figure 6. Electron energy loss spectroscopy analysis. a HRTEM image of Au/TiO_2@M-N-800. **b** EELS spectra of Au/TiO_2@M-N-800. The spectra were background-subtracted. **c** Fitted EELS spectra of Au/TiO_2@M-N-800 from 450 to 480 eV. The scale bar in **a** corresponds to 2 nm.

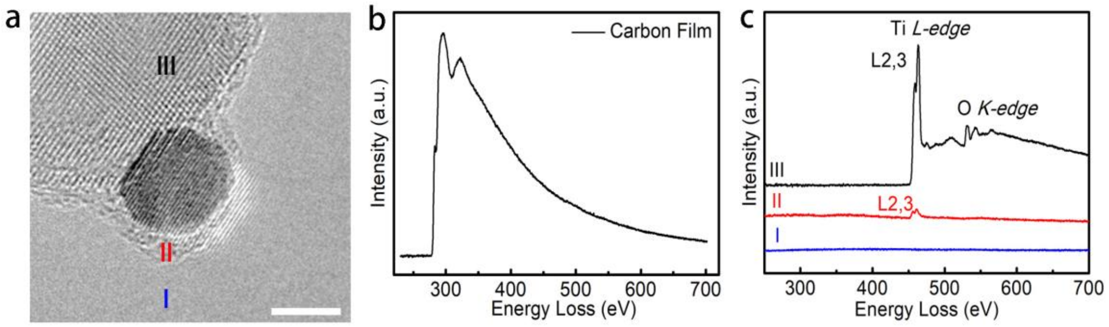

Supplementary Figure 7. Electron energy loss spectroscopy analysis. a HRTEM image of Au/TiO_2@M-N-800. **b** EELS spectra of carbon film. **c** EELS spectra of Au/TiO_2@M-N-800 from 250 to 700 eV. The spectra were background-subtracted. The scale bar in **a** corresponds to 5 nm.

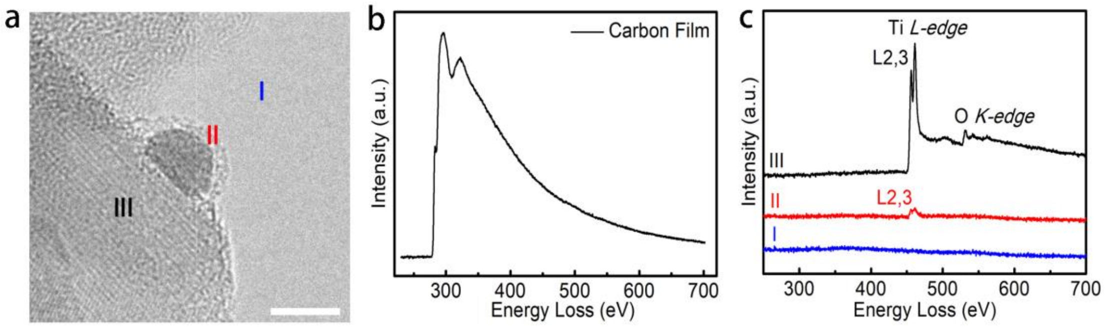

Supplementary Figure 8. Electron energy loss spectroscopy analysis. a HRTEM image of Au/TiO_2@M-N-800. **b** EELS spectra of carbon film. **c** EELS spectra of Au/TiO_2@M-N-800 from 250 to 700 eV. The spectra were background-subtracted. The scale bar in **a** corresponds to 5 nm.

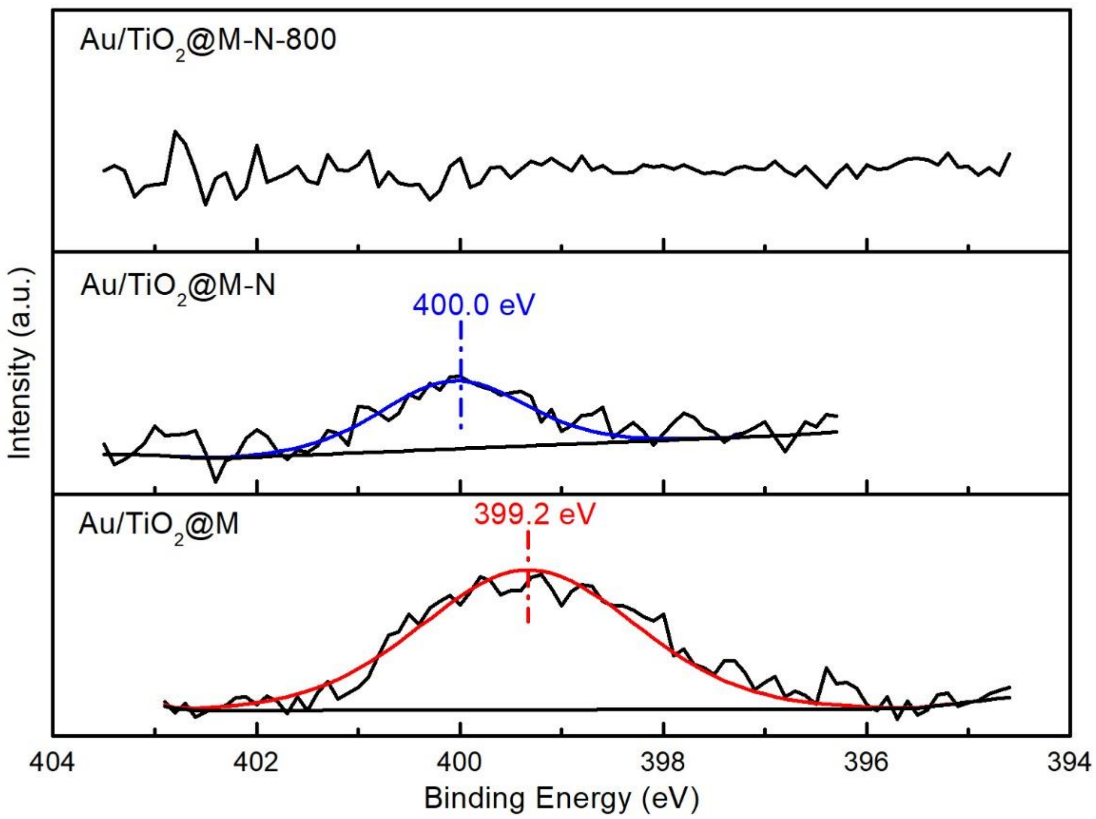

Supplementary Figure 9. N 1*s* XPS analysis. N 1*s* XPS spectra of Au/TiO_2@M, Au/TiO_2@M-N and Au/TiO_2@M-N-800.

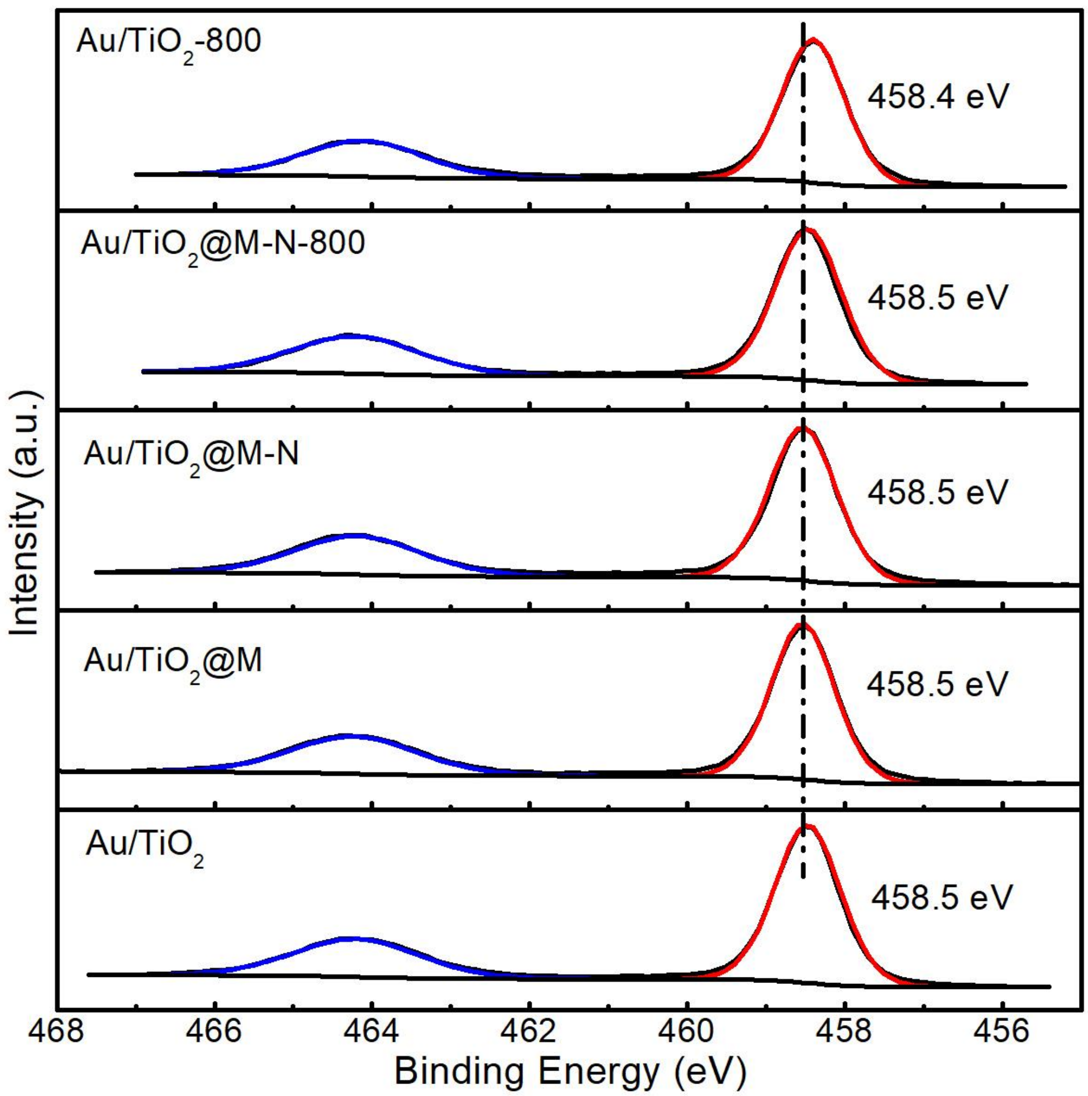

Supplementary Figure 10. Ti 2*p* XPS analysis. Ti 2*p* XPS spectra of Au/TiO_2, Au/TiO_2@M, Au/TiO_2@M-N, Au/TiO_2@M-N-800, Au/TiO_2-800.

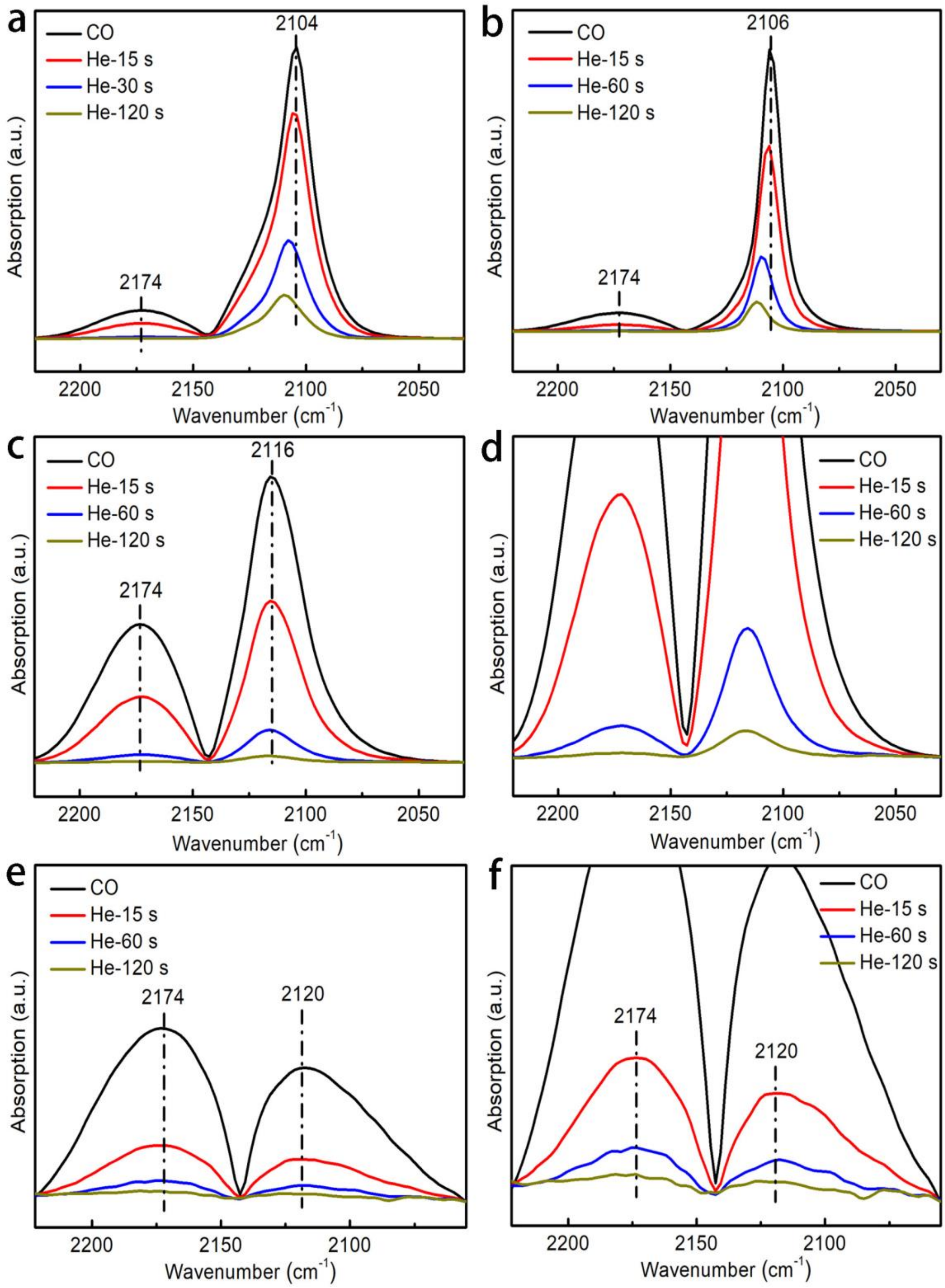

Supplementary Figure 11. DRIFTS analysis. In-situ DRIFT spectra of CO desorption on **a** Au/TiO_2, **b** Au/TiO_2@M-N, **c** Au/TiO_2@M-N-800 and **d** The enlargement of **c**, **e** The blank test, **f** The enlargement of **f** under the purge of He at room temperature.

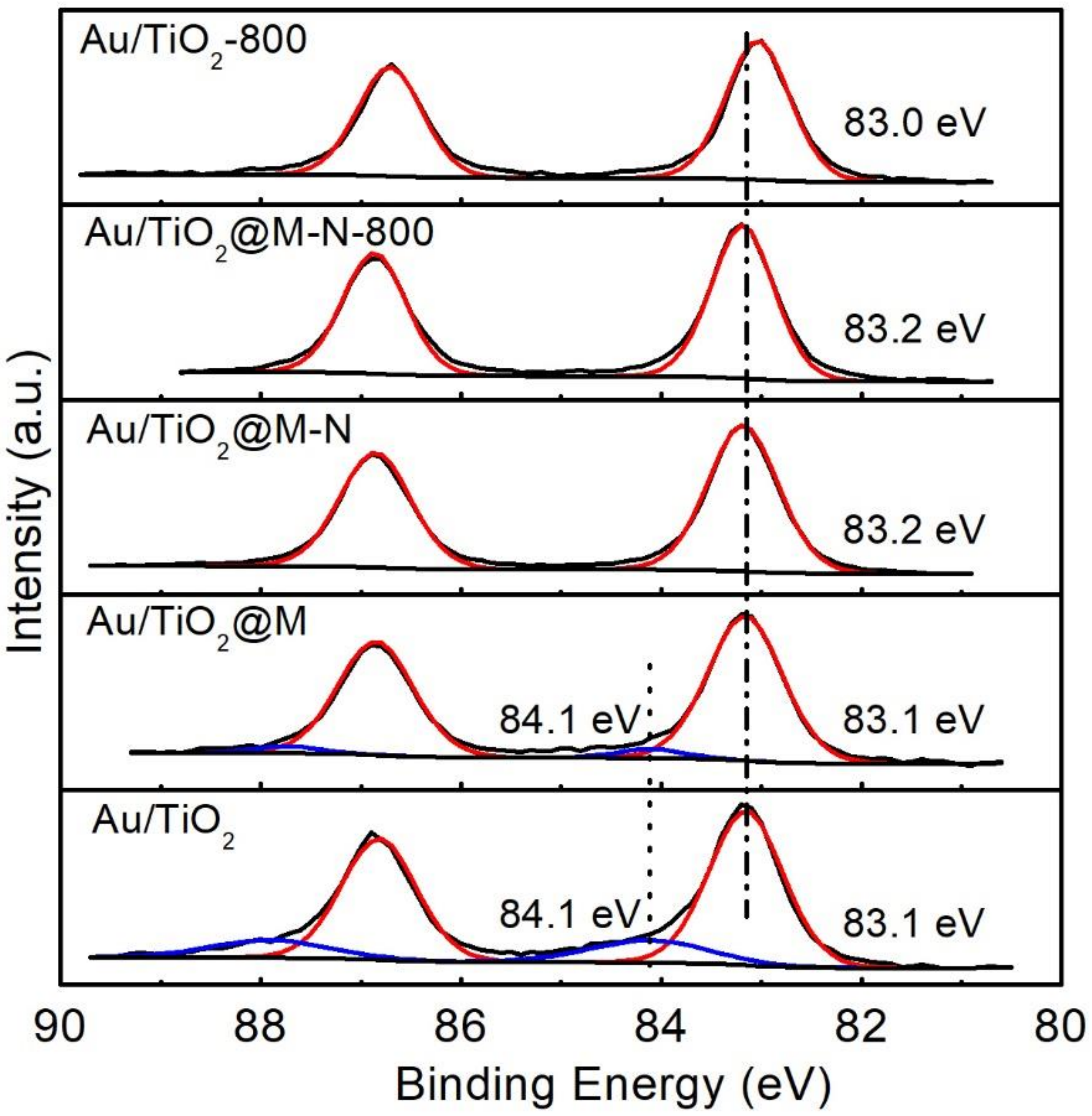

Supplementary Figure 12. Au 4*f* XPS analysis. Au 4*f* XPS spectra of Au/TiO_2, Au/TiO_2@M, Au/TiO_2@M-N, Au/TiO_2@M-N-800, and Au/TiO_2-800.

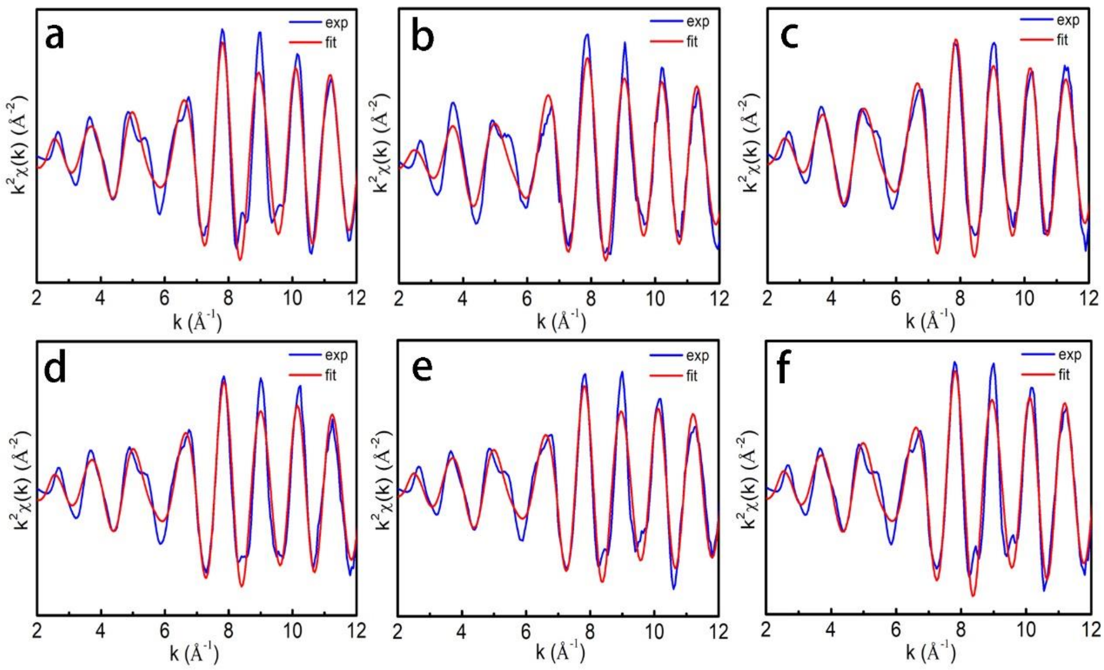

Supplementary Figure 13. **EXAFS characterization. a** Fitting Au L_{III} edge EXAFS spectra in *k*-space of Au foil. **b** Fitting Au L_{III} edge EXAFS spectra in *k*-space of Au/TiO_2. **c** Fitting Au L_{III} edge EXAFS spectra in *k*-space of Au/TiO_2@M. **d** Fitting Au L_{III} edge EXAFS spectra in *k*-space of Au/TiO_2@M-N. **e** Fitting Au L_{III} edge EXAFS spectra in *k*-space of Au/TiO_2@M-N-800. **f** Fitting Au L_{III} edge EXAFS spectra in *k*-space of Au/TiO_2-800, where the blue and red lines correspond to the experimental and curve-fitting results, respectively.

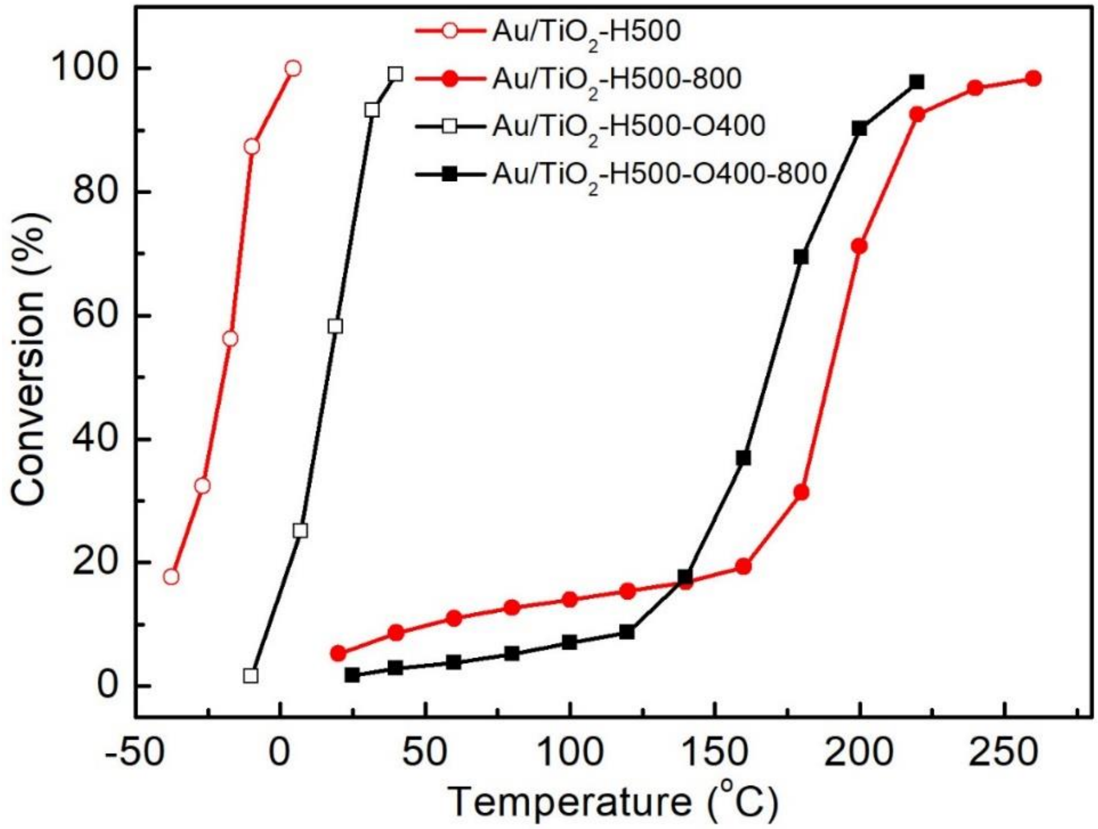

Supplementary Figure 14. Catalytic CO oxidation on Au/TiO_2 nanocatalysts before and after calcination. CO oxidation curves of Au/TiO_2-H500, Au/TiO_2-H500-O400, Au/TiO_2-H500-800 and Au/TiO_2-H500-O400-800 with a feed gas comprising 1 vol% CO/ 1 vol% O_2/ 98 vol% He at 33.3 mL min^{-1}.

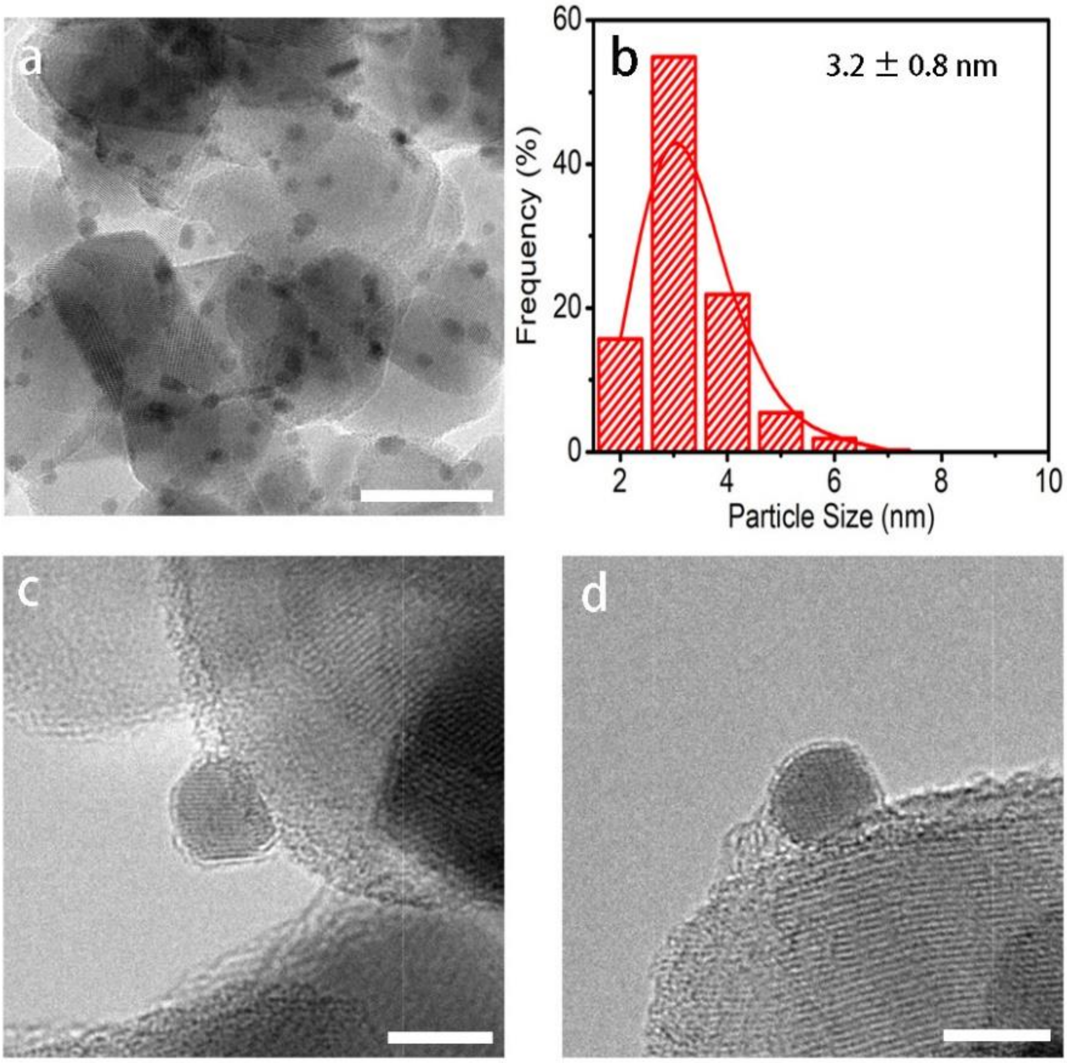

Supplementary Figure 15. Electron microscopy images and particle size distribution of Au/TiO$_2$-H500. a TEM image of Au/TiO$_2$-H500. **b** Particle size distribution of Au/TiO$_2$-H500. **c** and **d** HRTEM images of Au/TiO$_2$-H500, in which Au NPs were covered by TiO$_x$ overlayer. The scale bar in **a** corresponds to 20 nm, and in **c** and **d** corresponds to 5 nm.

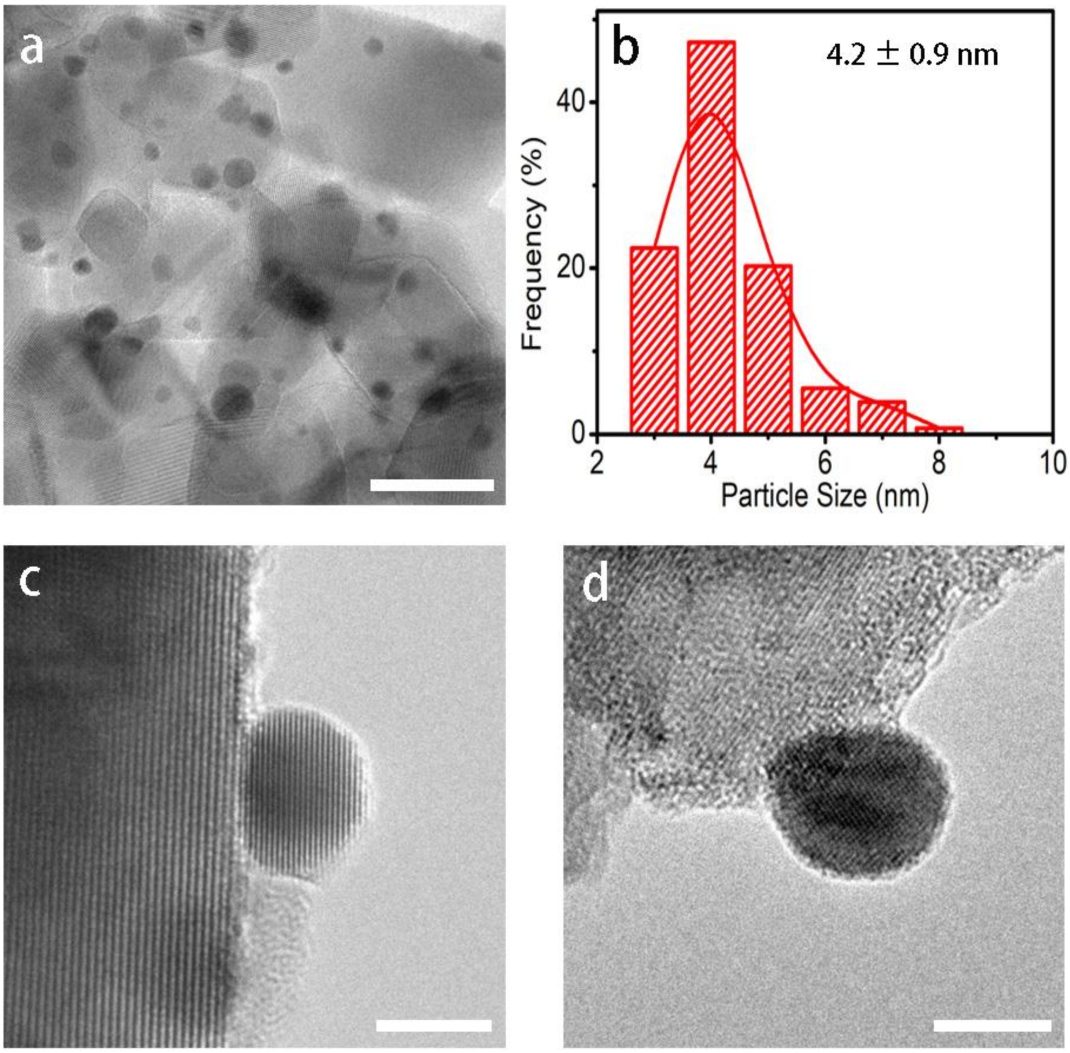

Supplementary Figure 16. Electron microscopy images and particle size distribution of Au/TiO$_2$-H500-O400. **a** TEM image of Au/TiO$_2$-H500-O400. **b** Particle size distribution of Au/TiO$_2$-H500-O400. **c** and **d** HRTEM images of Au/TiO$_2$-H500-O400, in which the overlayer retreated and Au NPs were bare. The scale bar in **a** corresponds to 20 nm, and in **c** and **d** corresponds to 5 nm.

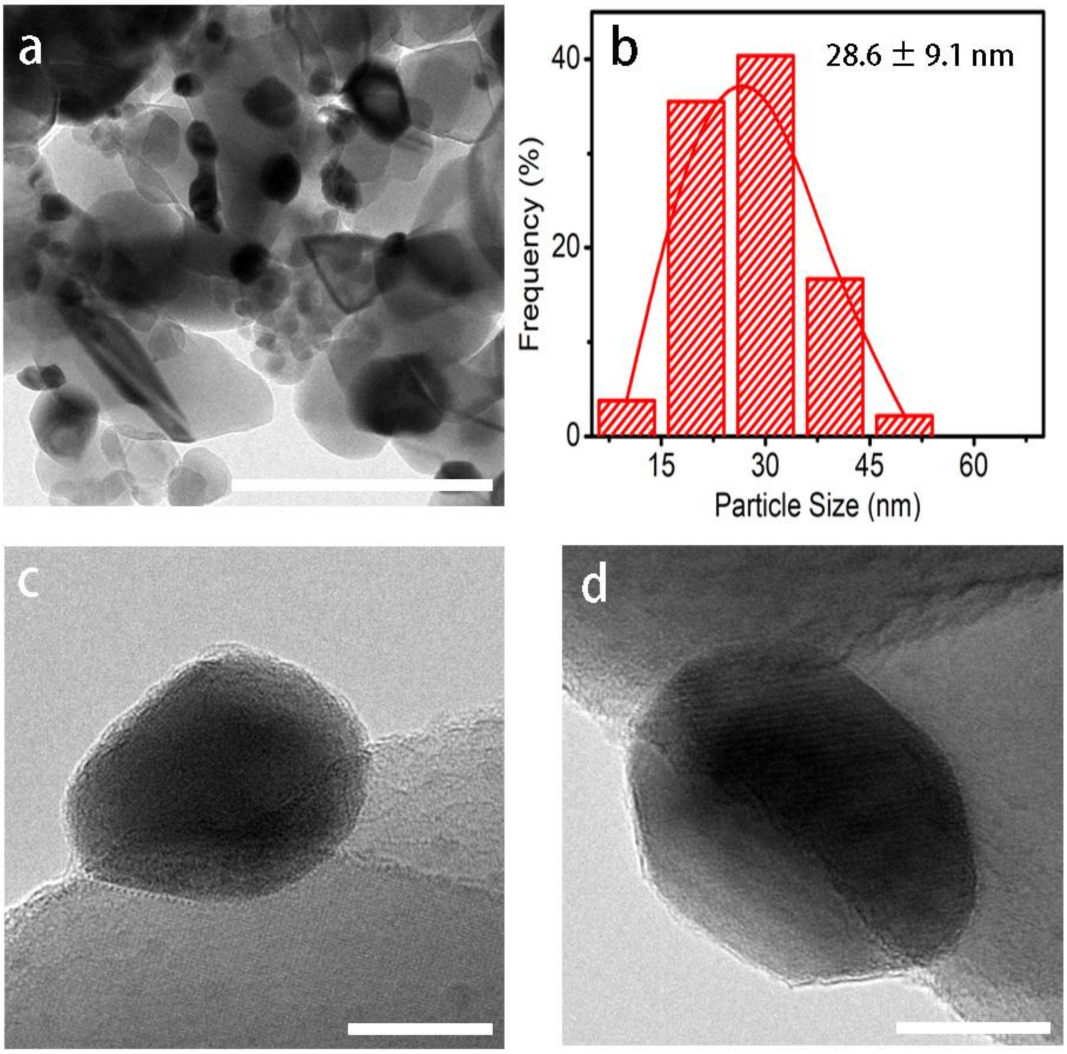

Supplementary Figure 17. Electron microscopy images and particle size distribution of Au/TiO_2-H500-800. **a** TEM image of Au/TiO_2-H500-800. **b** Particle size distribution of Au/TiO_2-H500-800. **c** and **d** HRTEM images of Au/TiO_2-H500-800, in which Au NPs sintered seriously. The scale bar in **a** corresponds to 200 nm, and in **c** and **d** corresponds to 10 nm.

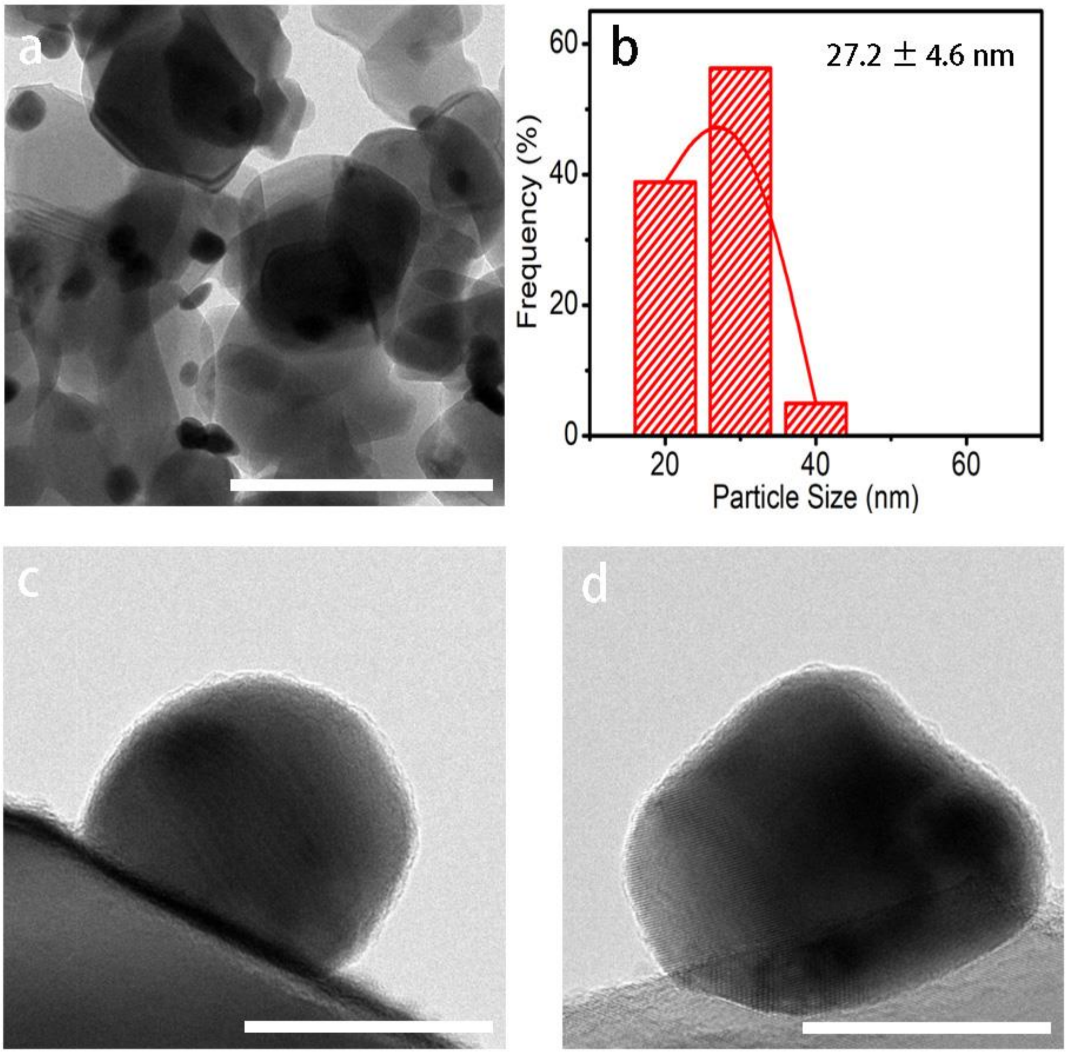

Supplementary Figure 18. Electron microscopy images and particle size distribution of Au/TiO_2-H500-O400-800. a TEM image of Au/TiO_2-H500-O400-800. **b** Particle size distribution of Au/TiO_2-H500-O400-800. **c** and **d** HRTEM images of Au/TiO_2-H500-O400-800, in which Au NPs sintered seriously. The scale bar in **a** corresponds to 200 nm, and in **c** and **d** corresponds to 20 nm.

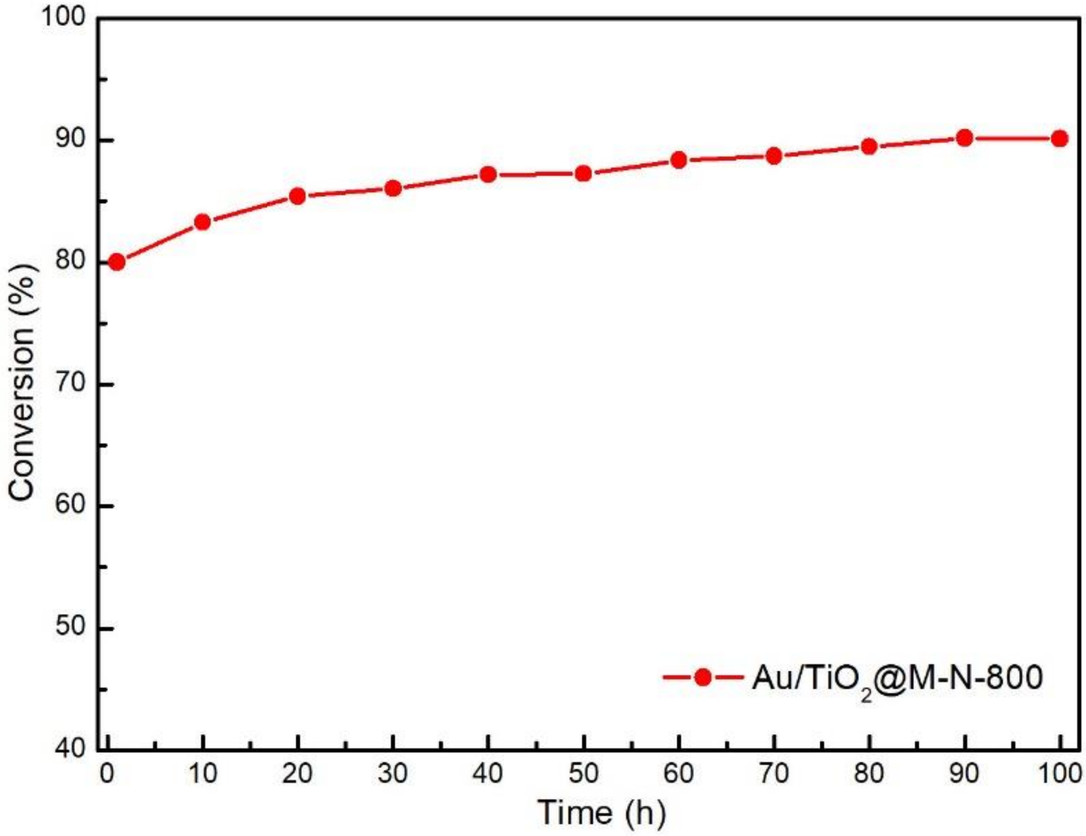

Supplementary Figure 19. Catalytic CO oxidation on Au/TiO_2@M-N-800 nanocatalyst. Long –term CO oxidation at 400 ^oC on Au/TiO_2@M-N-800 with a feed gas comprising 1 vol% CO/ 1 vol% O_2/ 98 vol% He at 33.3 mL min^{-1}, 4.2 mg catalyst, space velocity (SV) = 500 L h^{-1} g_{cat}^{-1}.

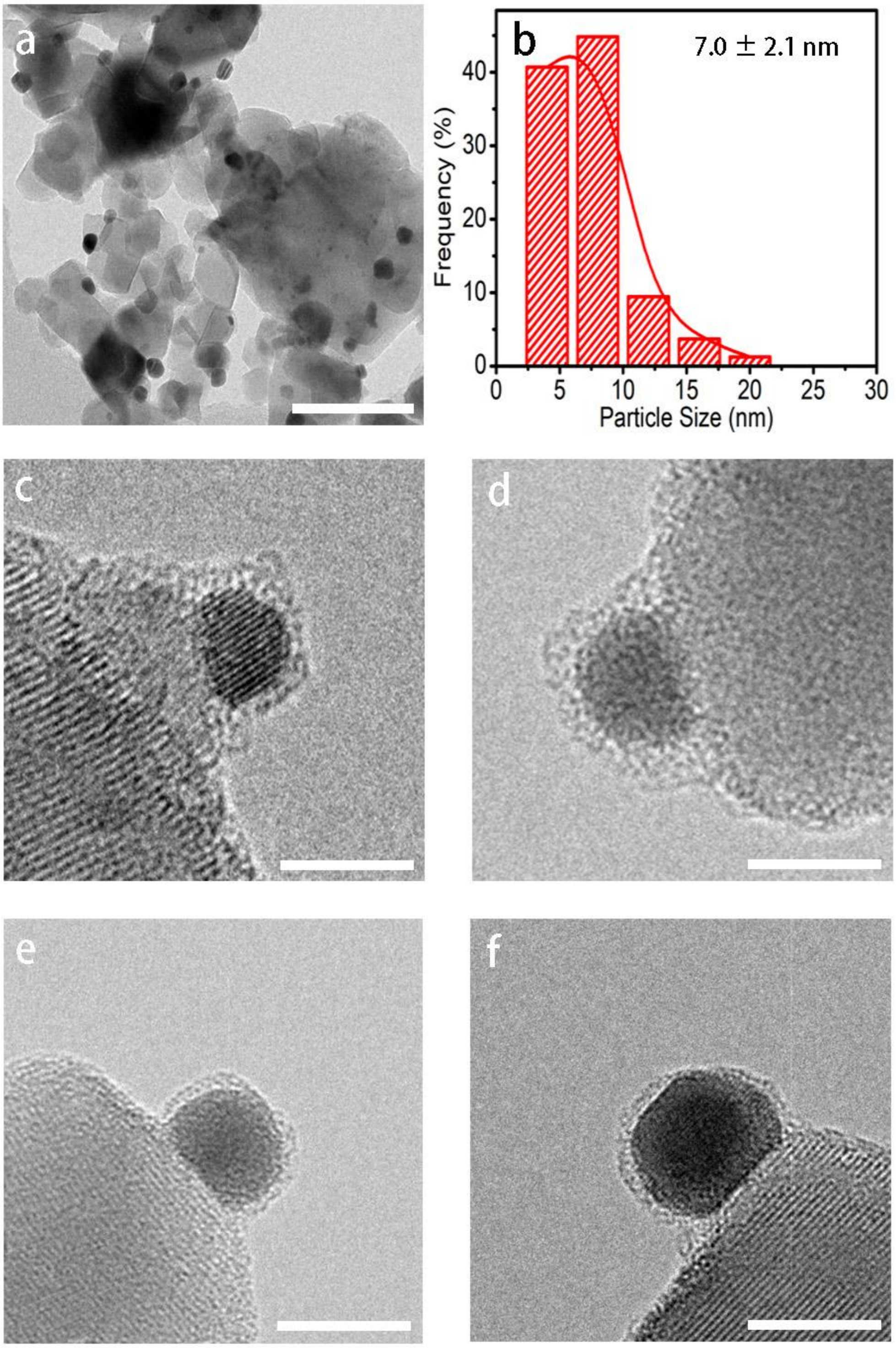

Supplementary Figure 20. Electron microscopy images and particle size distribution of Au/TiO_2@M-N-800. **a** TEM image of Au/TiO_2@M-N-800 after catalyzing CO oxidation at 400 °C for 100 h. **b** Particle size distribution of Au/TiO_2@M-N-800 after catalyzing CO oxidation at 400 °C for 100 h. **c** and **d** HRTEM images of Au/TiO_2@M-N-800 after catalyzing CO oxidation at 400 °C for 100 h, in which Au NPs were still covered by TiO_x overlayer. **e** and **f** HRTEM images of Au/TiO_2@M-N-800 after catalyzing CO oxidation at 400 °C for 100 h, in which the TiO_x overlayer was not dense as that of the fresh catalyst. The scale bar in **a** corresponds to 100 nm, and in **c**, **d**, **e** and **f** corresponds to 5 nm.

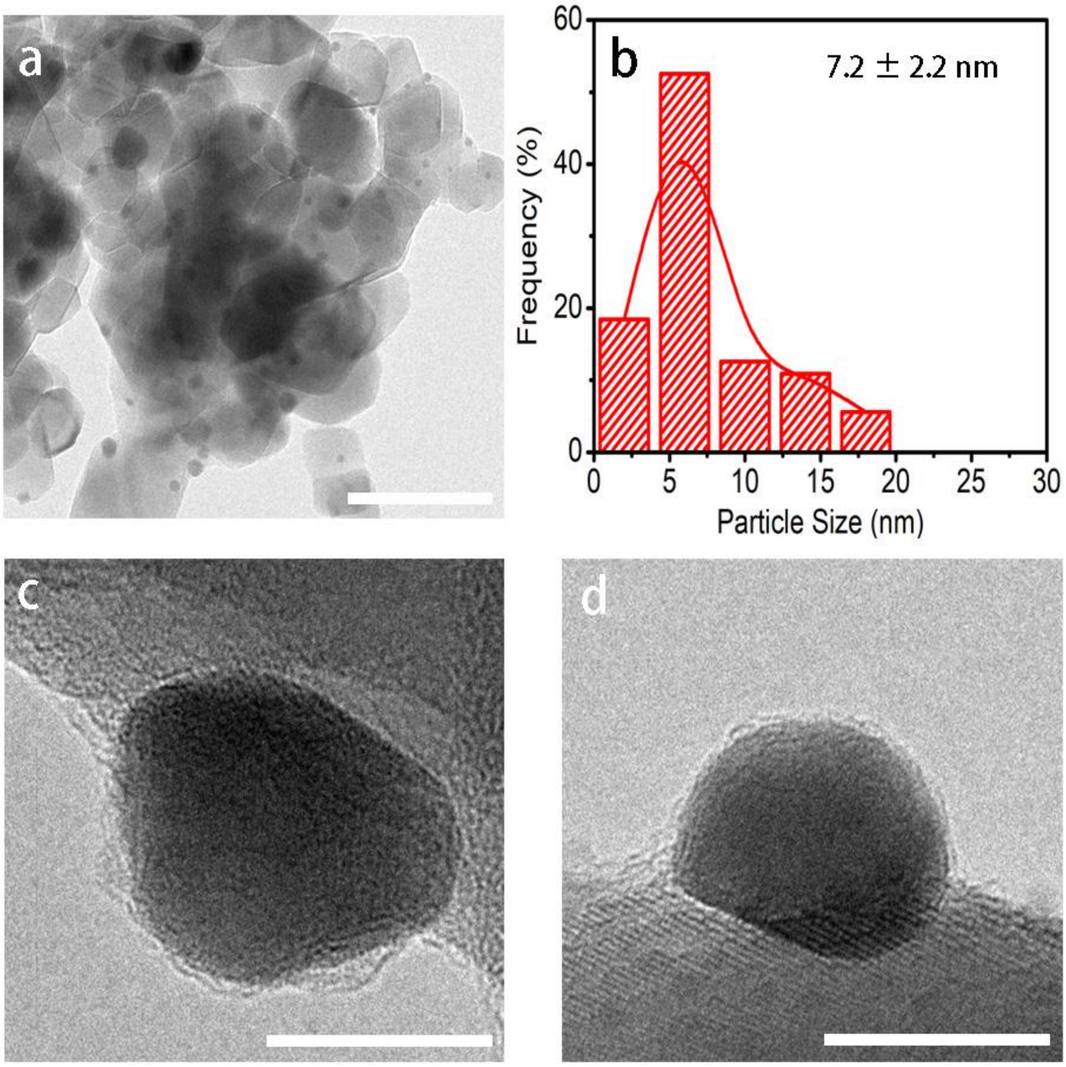

Supplementary Figure 21. Electron microscopy images and particle size distribution of Au/TiO_2@M-N-800 after 10th ignition-extinction cycles. a TEM image of Au/TiO_2@M-N-800 after 10th ignition-extinction cycles. **b** Particle size distribution of Au/TiO_2@M-N-800 after 10th ignition-extinction cycles. **c** and **d** HRTEM images of Au/TiO_2@M-N-800 after 10th ignition-extinction cycles, in which TiO_x overlayer still existed on the Au NPs. The scale bar in **a** corresponds to 50 nm, and in **c** and **d** corresponds to 10 nm.

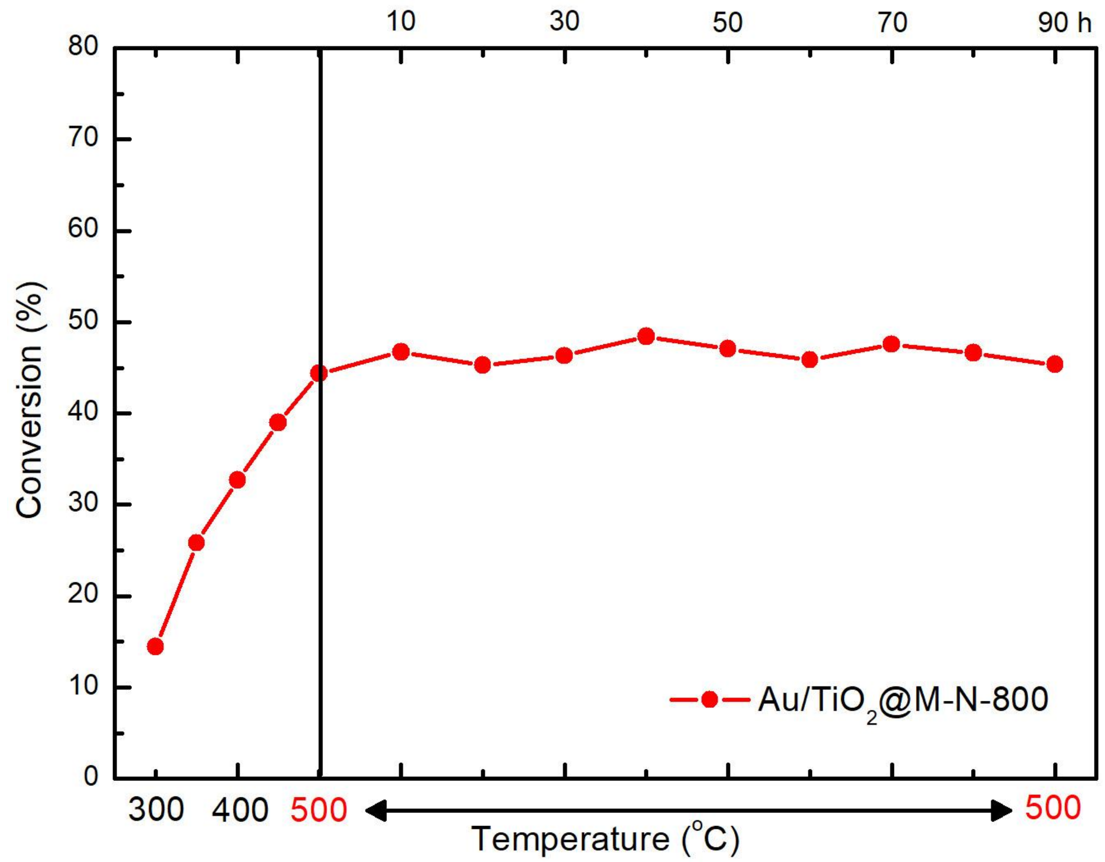

Supplementary Figure 22. Water-gas shift (WGS) reaction on Au/TiO_2@M-N-800 nanocatalyst. Long–term WGS reaction at 500 ºC on Au/TiO_2@M-N-800 with a feed gas comprising 2 vol% CO/ 10 vol% H_2O/ 88 vol% He at 30 mL min^{-1}, 0.1 g catalyst, space velocity (SV) = 18 L h^{-1} g_{cat}^{-1}.

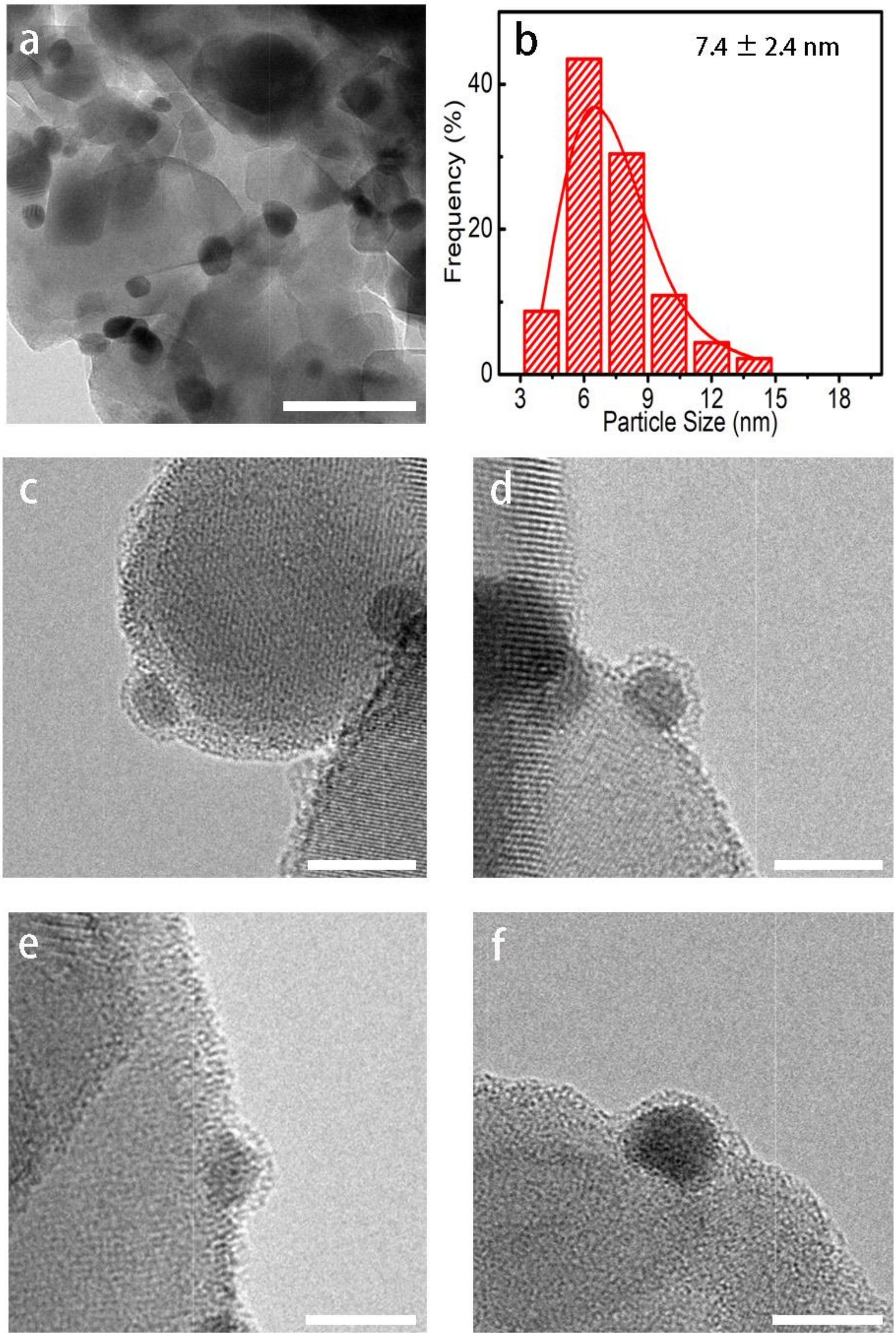

Supplementary Figure 23. Electron microscopy images and particle size distribution of Au/TiO_2@M-N-800. **a** TEM image of Au/TiO_2@M-N-800 after WGS reaction at 500 °C for 90 h. **b** Particle size distribution of Au/TiO_2@M-N-800 after WGS reaction at 500 °C for 90 h. **c**, **d, e** and **f** HRTEM images of Au/TiO_2@M-N-800 after WGS reaction at 500 °C for 90 h, in which Au NPs were still covered by TiO_x overlayer. The scale bar in **a** corresponds to 100 nm, and in **c**, **d**, **e** and **f** corresponds to 5 nm.

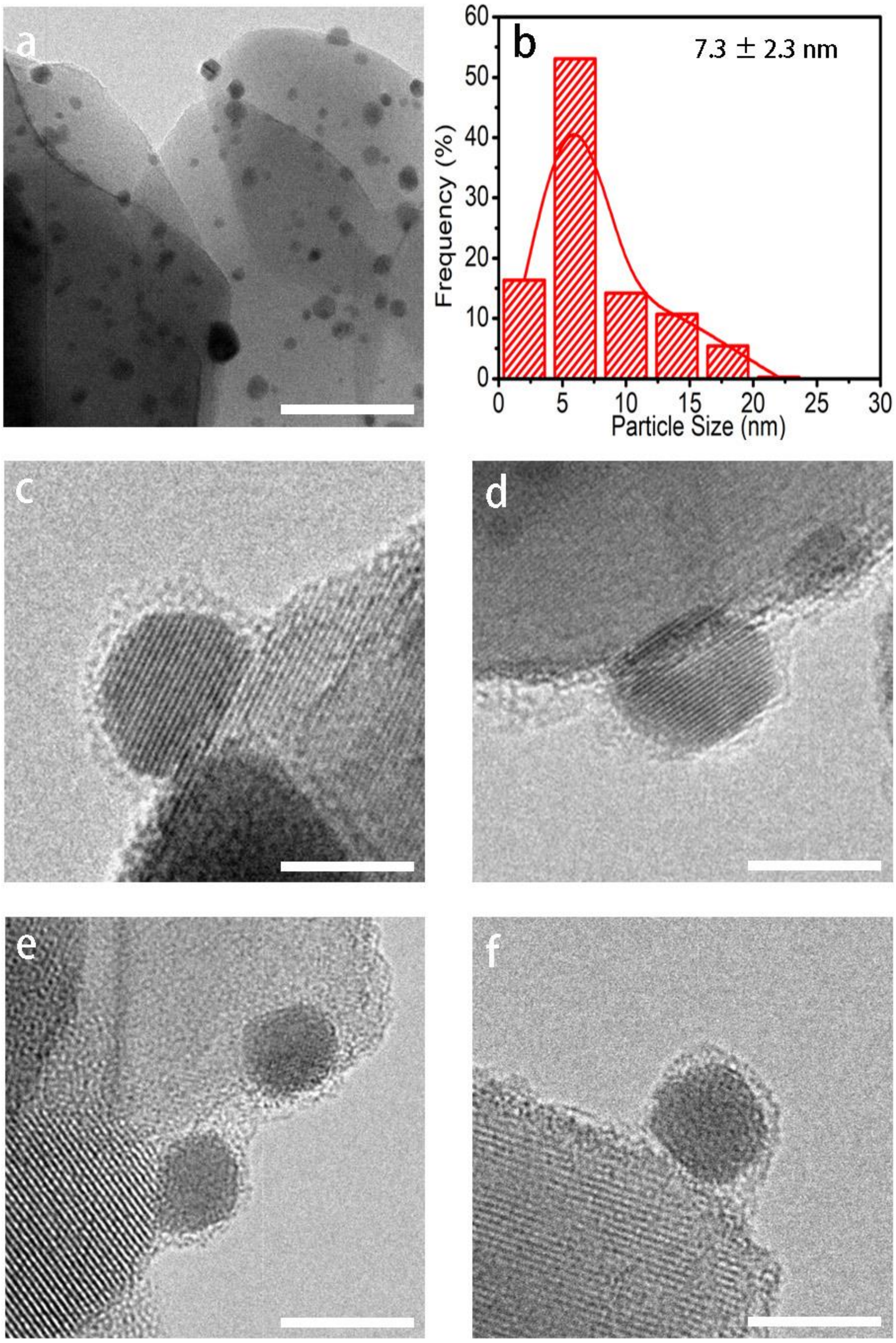

Supplementary Figure 24. Electron microscopy images and particle size distribution of Au/TiO$_2$@M-N-800 after simulated CO emission control reaction at 400 ^{o}C for 10 days. a TEM image of Au/TiO$_2$@M-N-800 after simulated CO emission control reaction at 400 ^{o}C for 10 days. **b** Particle size distribution of Au/TiO$_2$@M-N-800 after simulated CO emission control reaction at 400 ^{o}C for 10 days. **c** HRTEM image of Au/TiO$_2$@M-N-800 after simulated CO emission control reaction at 400 ^{o}C for 10 days, in which Au NPs were still encapsulted by TiO$_x$ overlayer. **d**, **e** and **f** HRTEM images of Au/TiO$_2$@M-N-800 after simulated CO emission control reaction at 400 ^{o}C for 10 days, in which the TiO$_x$ overlayer was not dense as that of the fresh catalyst. The scale bar in **a** corresponds to 50 nm, and in **c**, **d**, **e** and **f** corresponds to 5 nm.

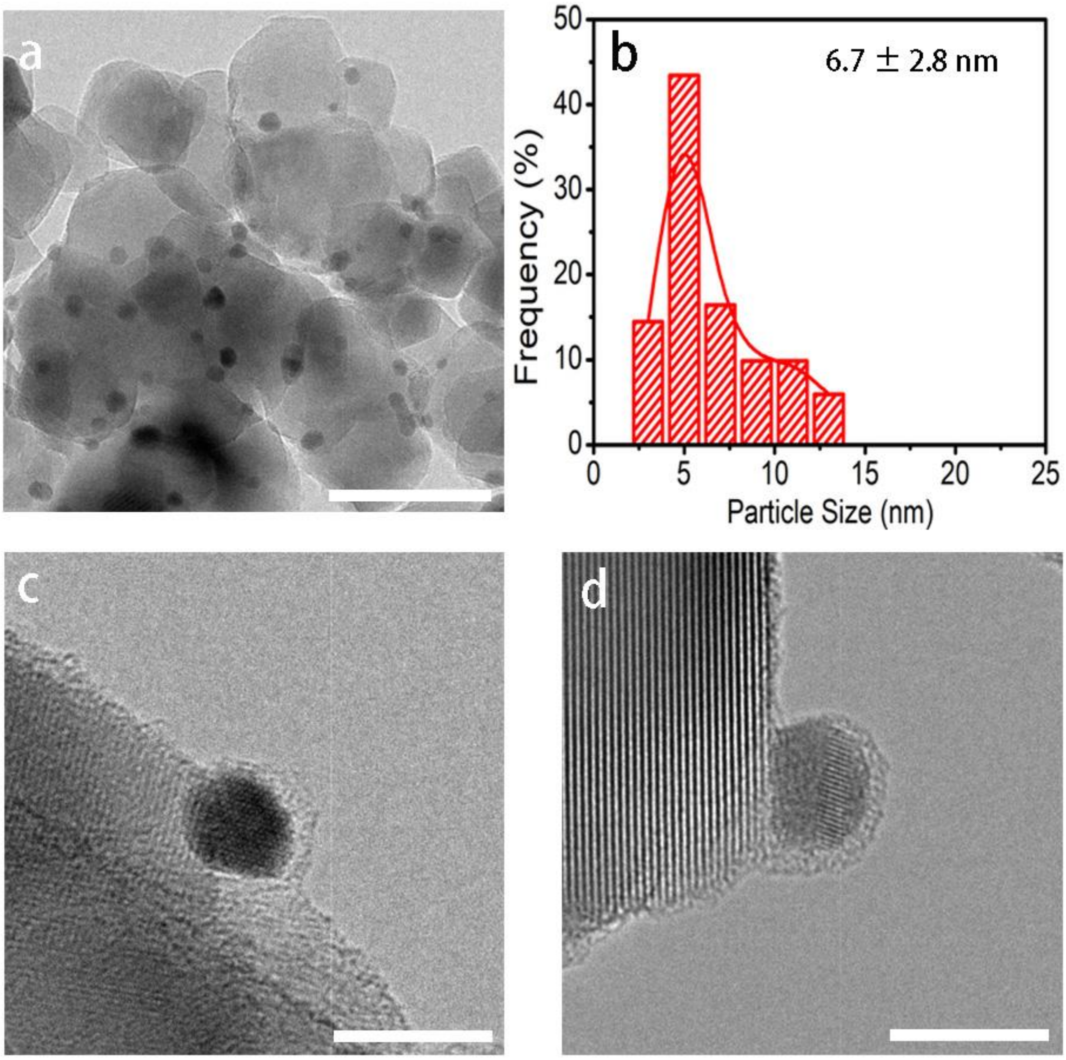

Supplementary Figure 25. Electron microscopy images and particle size distribution of Au/TiO_2@M-N-800-400. **a** TEM image of Au/TiO_2@M-N-800-400. **b** Particle size distribution of Au/TiO_2@M-N-800-400. **c** and **d** HRTEM images of Au/TiO_2@M-N-800-400, in which TiO_x overlayer did not retreat. The scale bar in **a** corresponds to 50 nm, and in **c** and **d** corresponds to 5 nm.

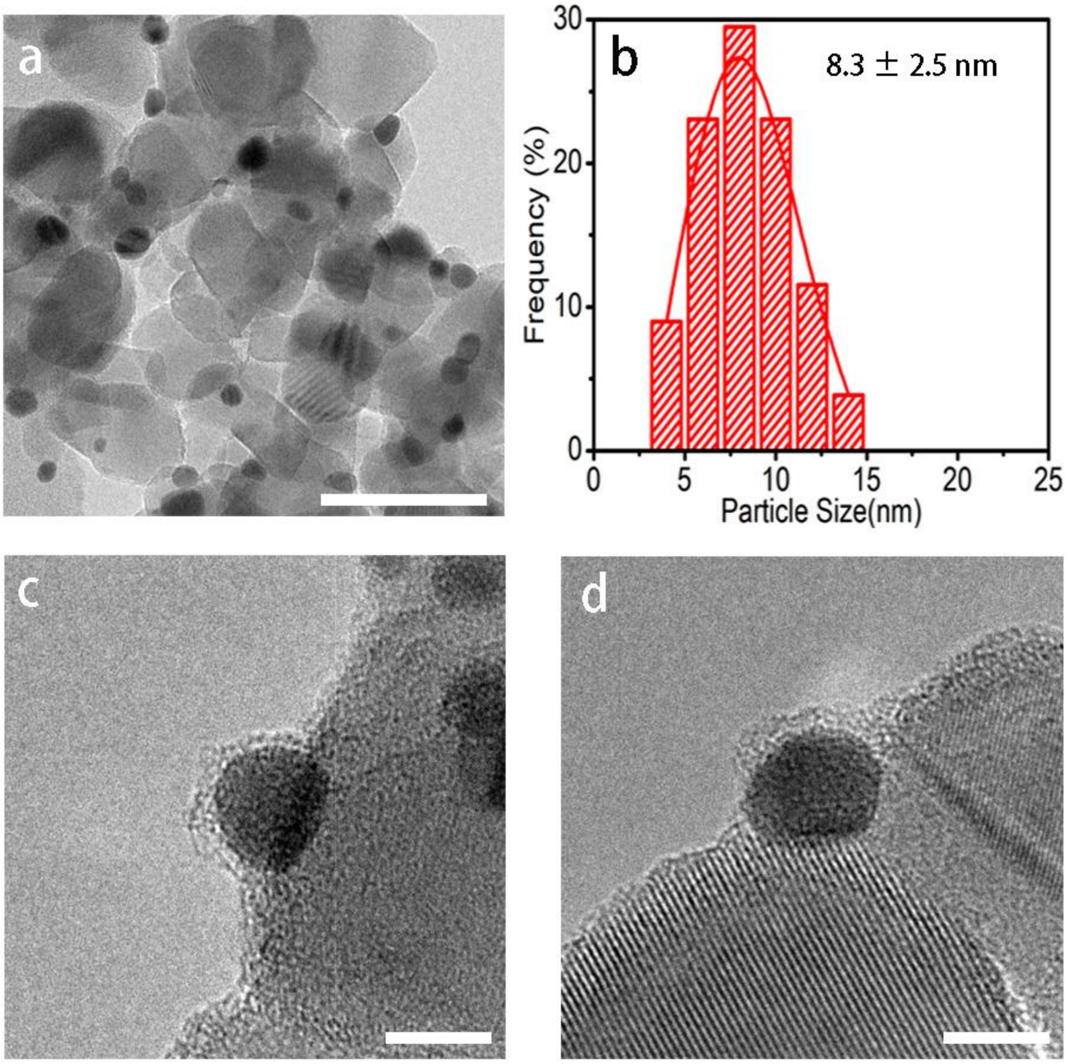

Supplementary Figure 26. Electron microscopy images and particle size distribution of Au/TiO$_2$@M-N-800-500. **a** TEM image of Au/TiO$_2$@M-N-800-500. **b** Particle size distribution of Au/TiO$_2$@M-N-800-500. **c** and **d** HRTEM images of Au/TiO$_2$@M-N-800-500, in which TiO$_x$ overlayer did not retreat. The scale bar in **a** corresponds to 50 nm, and in **c** and **d** corresponds to 5 nm.

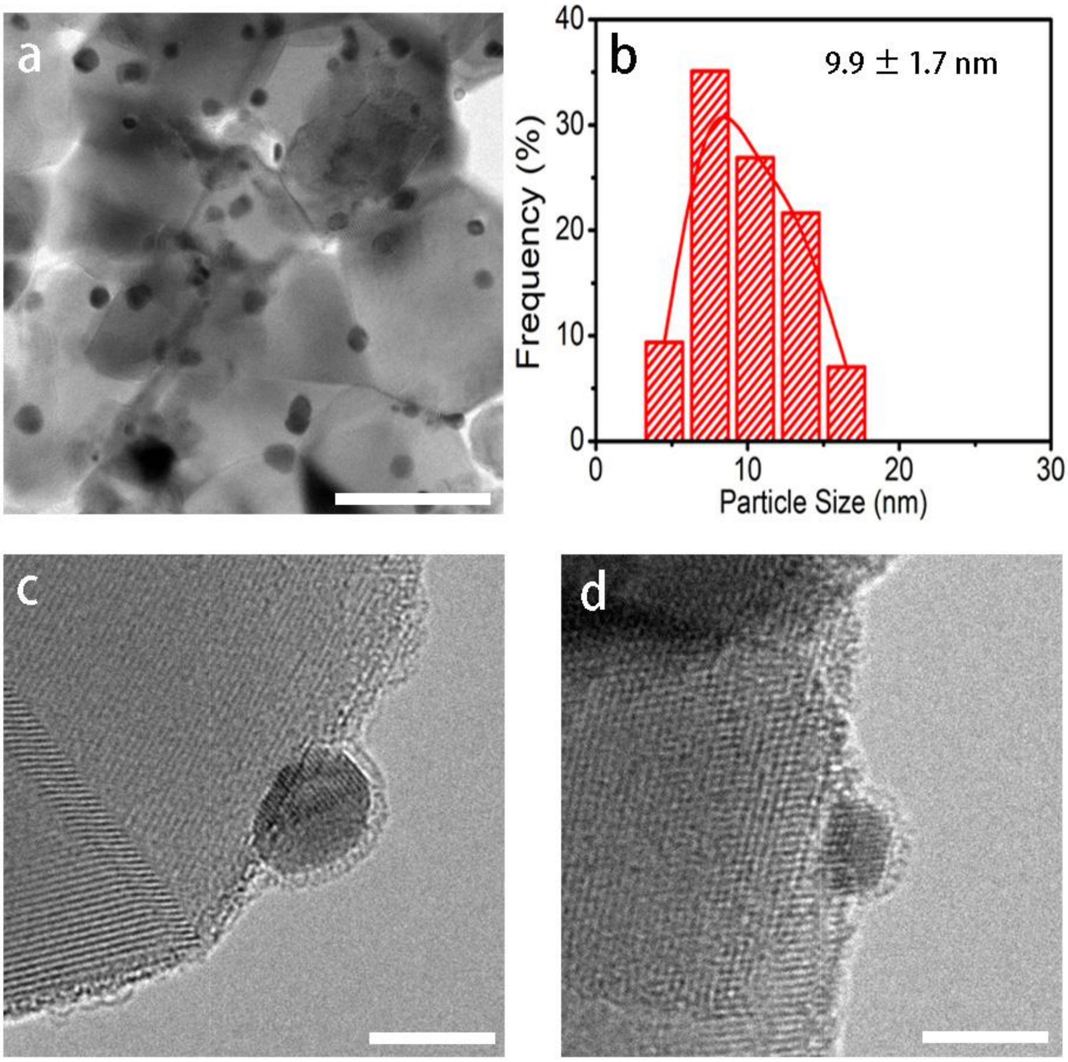

Supplementary Figure 27. Electron microscopy images and particle size distribution of Au/TiO_2@M-N-800-600. a TEM image of Au/TiO_2@M-N-800-600. **b** Particle size distribution of Au/TiO_2@M-N-800-600. **c** and **d** HRTEM images of Au/TiO_2@M-N-800-600, in which TiO_x overlayer did not retreat. The scale bar in **a** corresponds to 100 nm, and in **c** and **d** corresponds to 5 nm.

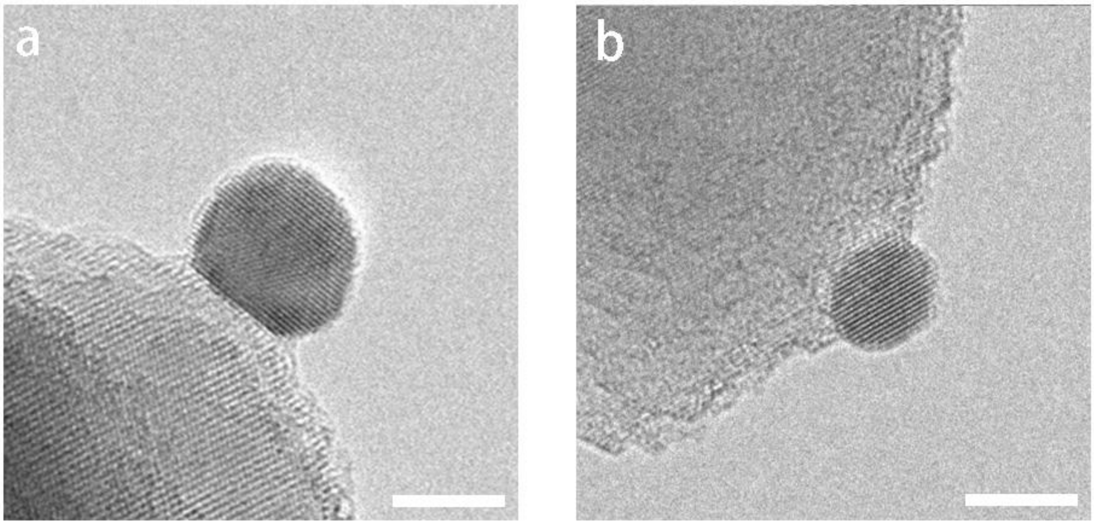

Supplementary Figure 28. HRTEM analysis. a and **b** HRTEM images of Au/TiO_2@M-N, in which Au NPs were bare. The scale bars are all 5 nm.

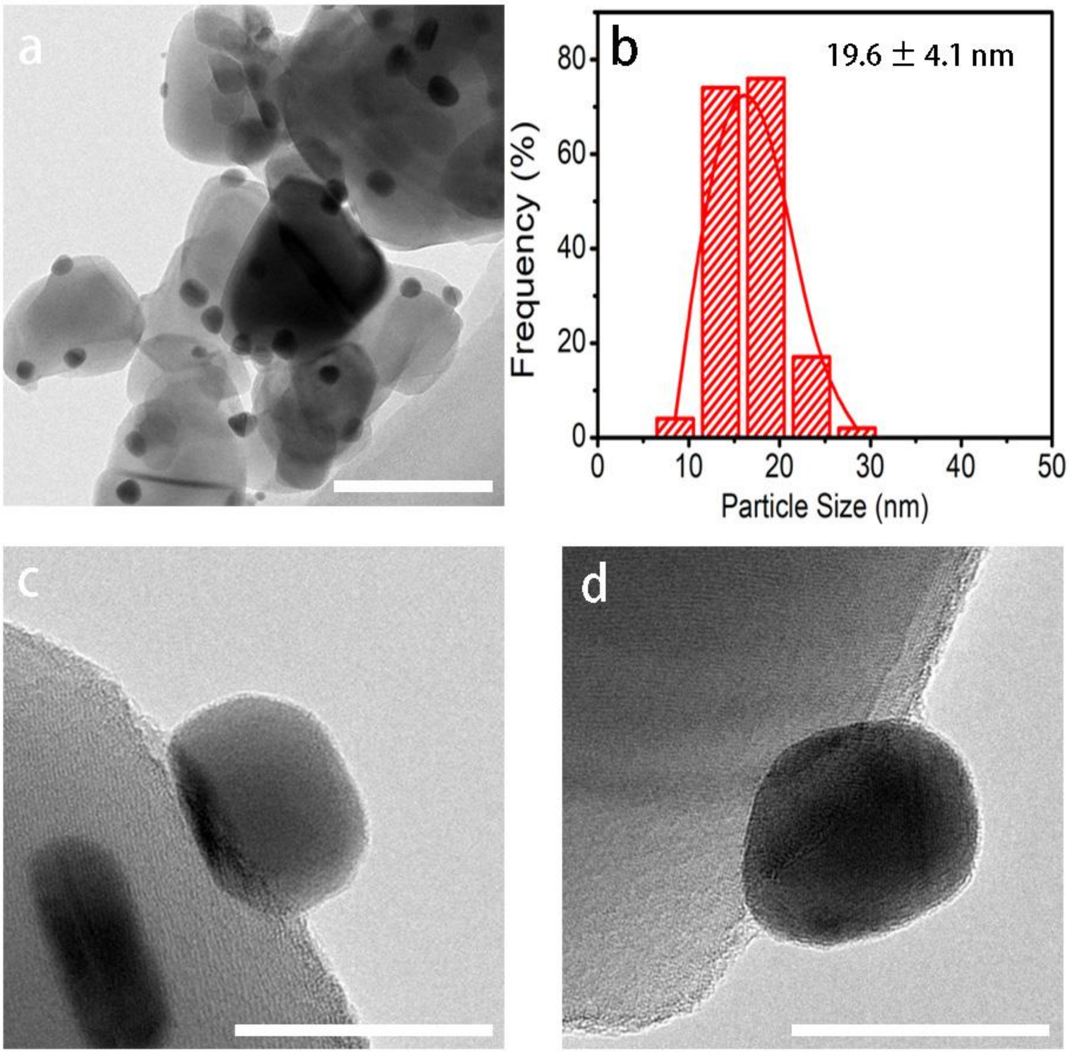

Supplementary Figure 29. Electron microscopy images and particle size distribution of Au/TiO_2@M-N800. **a** TEM image of Au/TiO_2@M-N800. **b** Particle size distribution of Au/TiO_2@M-N800. **c** and **d** HRTEM images of Au/TiO_2@M-N800, in which Au NPs sintered seriously. The scale bar in **a** corresponds to 100 nm, and in **c** and **d** corresponds to 20 nm.

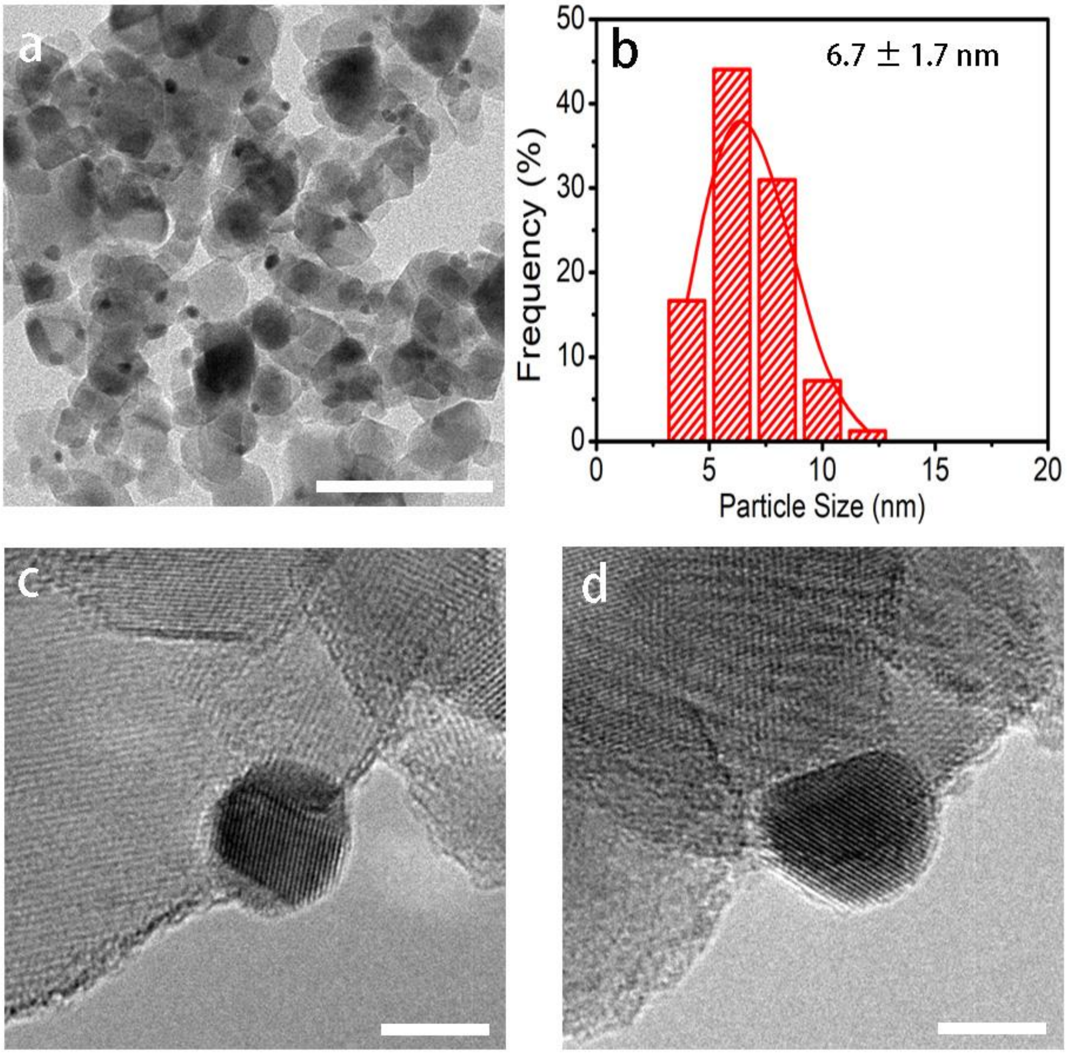

Supplementary Figure 30. Electron microscopy images and particle size distribution of Au/TiO_2@M-600. **a** TEM image of Au/TiO_2@M-600. **b** Particle size distribution of Au/TiO_2@M-600. **c** and **d** HRTEM images of Au/TiO_2@M-600, in which no overlayer was observed. The scale bar in **a** corresponds to 100 nm, and in **c** and **d** corresponds to 5 nm.

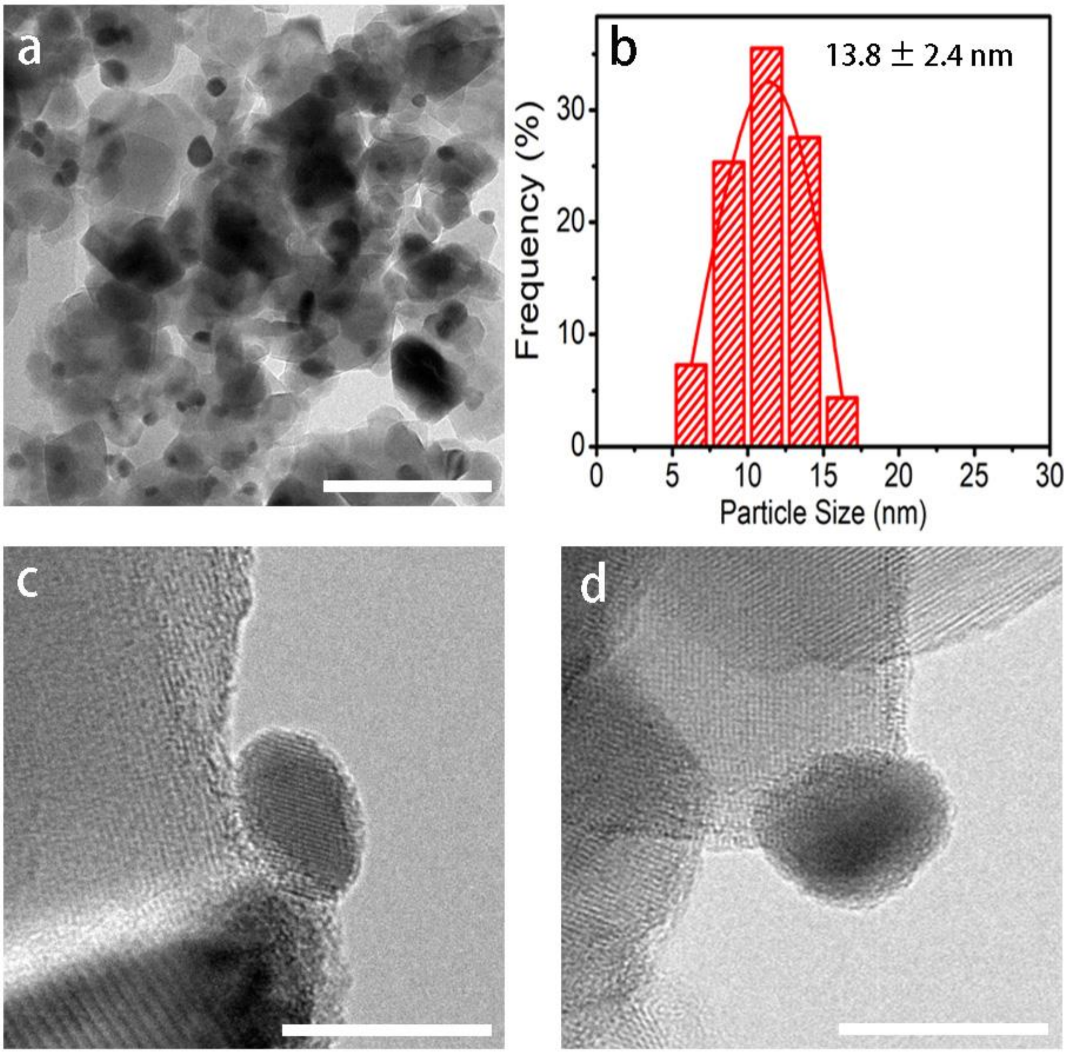

Supplementary Figure 31. Electron microscopy images and particle size distribution of Au/TiO$_2$@M-800. **a** TEM image of Au/TiO$_2$@M-800. **b** Particle size distribution of Au/TiO$_2$@M-800. **c** and **d** HRTEM images of Au/TiO$_2$@M-800, in which no overlayer was observed. The scale bar in **a** corresponds to 100 nm, and in **c** and **d** corresponds to 10 nm.

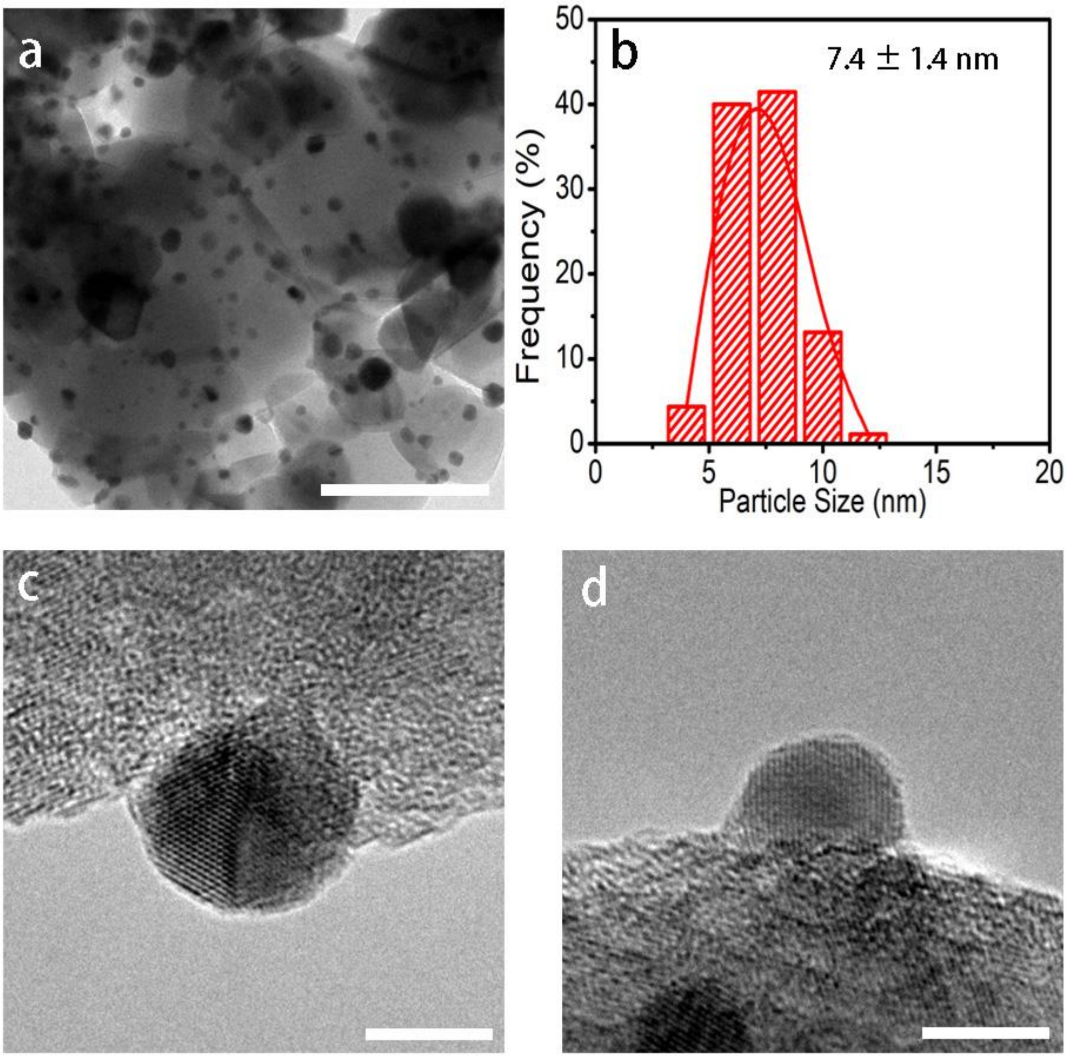

Supplementary Figure 32. Electron microscopy images and particle size distribution of Au/TiO$_2$@M-N-500. **a** TEM image of Au/TiO$_2$@M-N-500. **b** Particle size distribution of Au/TiO$_2$@M-N-500. **c** and **d** HRTEM images of Au/TiO$_2$@M-N-500, in which no overlayer was observed. The scale bar in **a** corresponds to 100 nm, and in **c** and **d** corresponds to 5 nm.

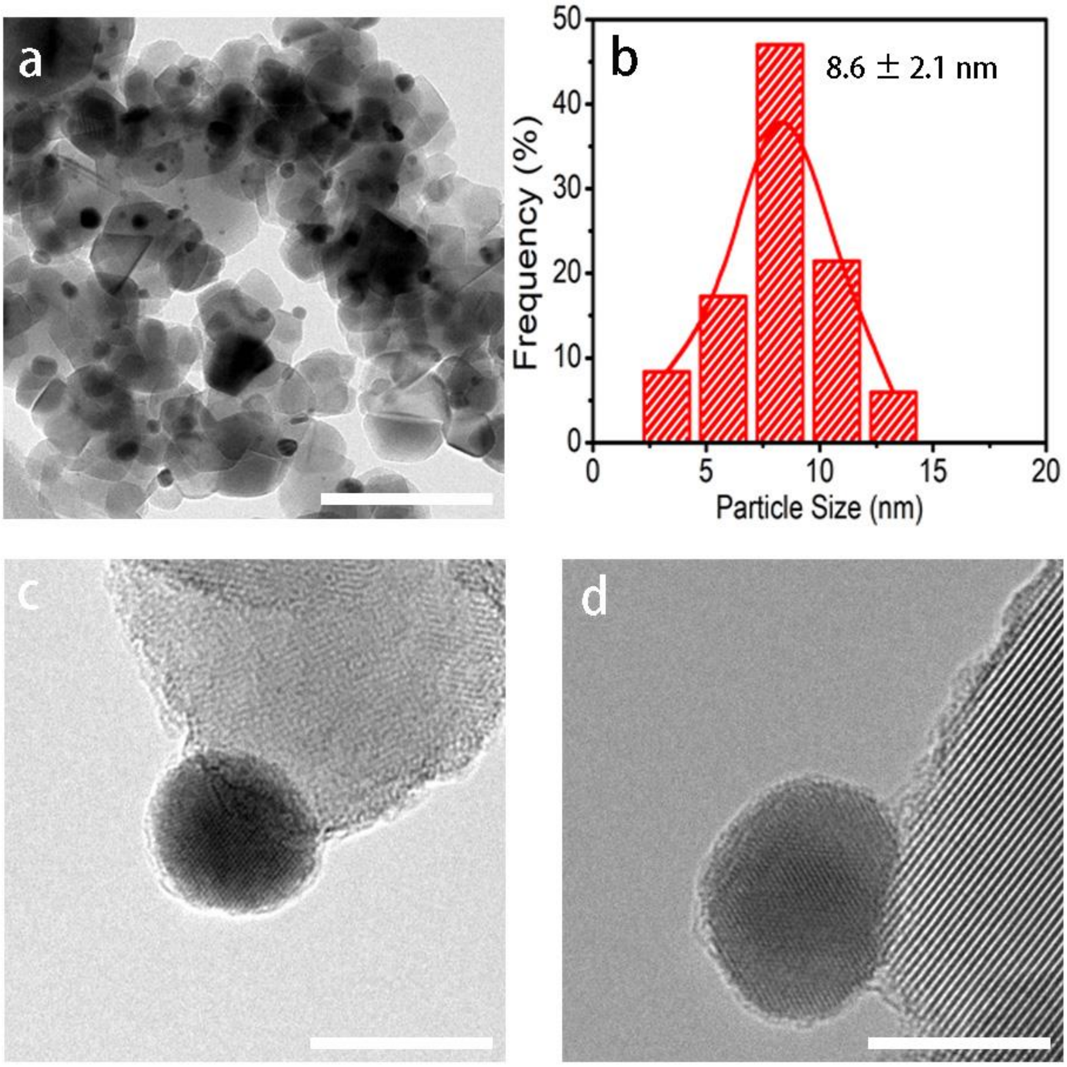

Supplementary Figure 33. Electron microscopy images and particle size distribution of Au/TiO_2@M-N-600. **a** TEM image of Au/TiO_2@M-N-600. **b** Particle size distribution of Au/TiO_2@M-N-600. **c** and **d** HRTEM images of Au/TiO_2@M-N-600, in which no overlayer was observed. The scale bar in **a** corresponds to 100 nm, and in **c** and **d** corresponds to 5 nm.

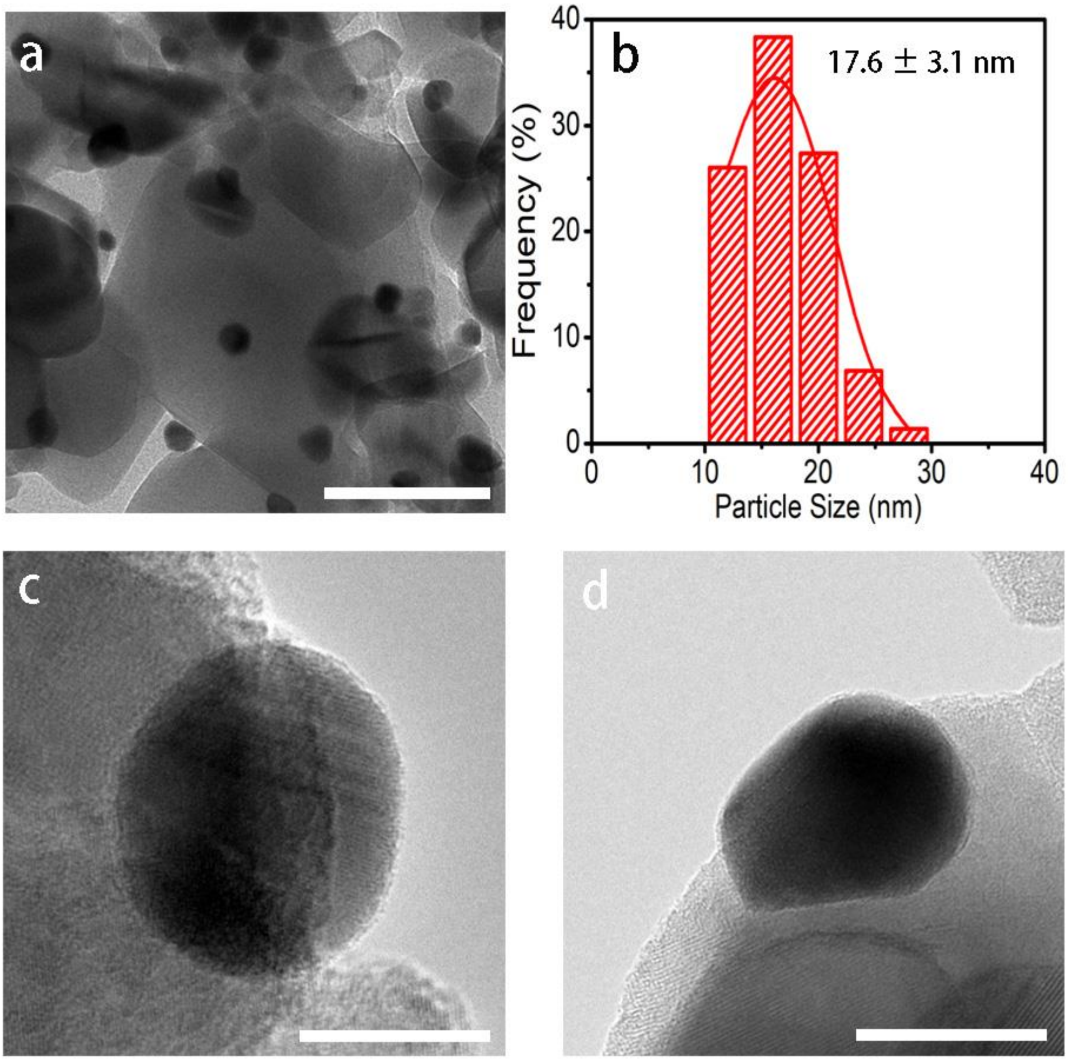

Supplementary Figure 34. Electron microscopy images and particle size distribution of Au/TiO_2-N-800. a TEM image of Au/TiO_2-N-800. **b** Particle size distribution of Au/TiO_2-N-800. **c** and **d** HRTEM images of Au/TiO_2-N-800, in which Au NPs sintered seriously and no overlayer was observed. The scale bar in **a** corresponds to 200 nm, and in **c** and **d** corresponds to 20 nm.

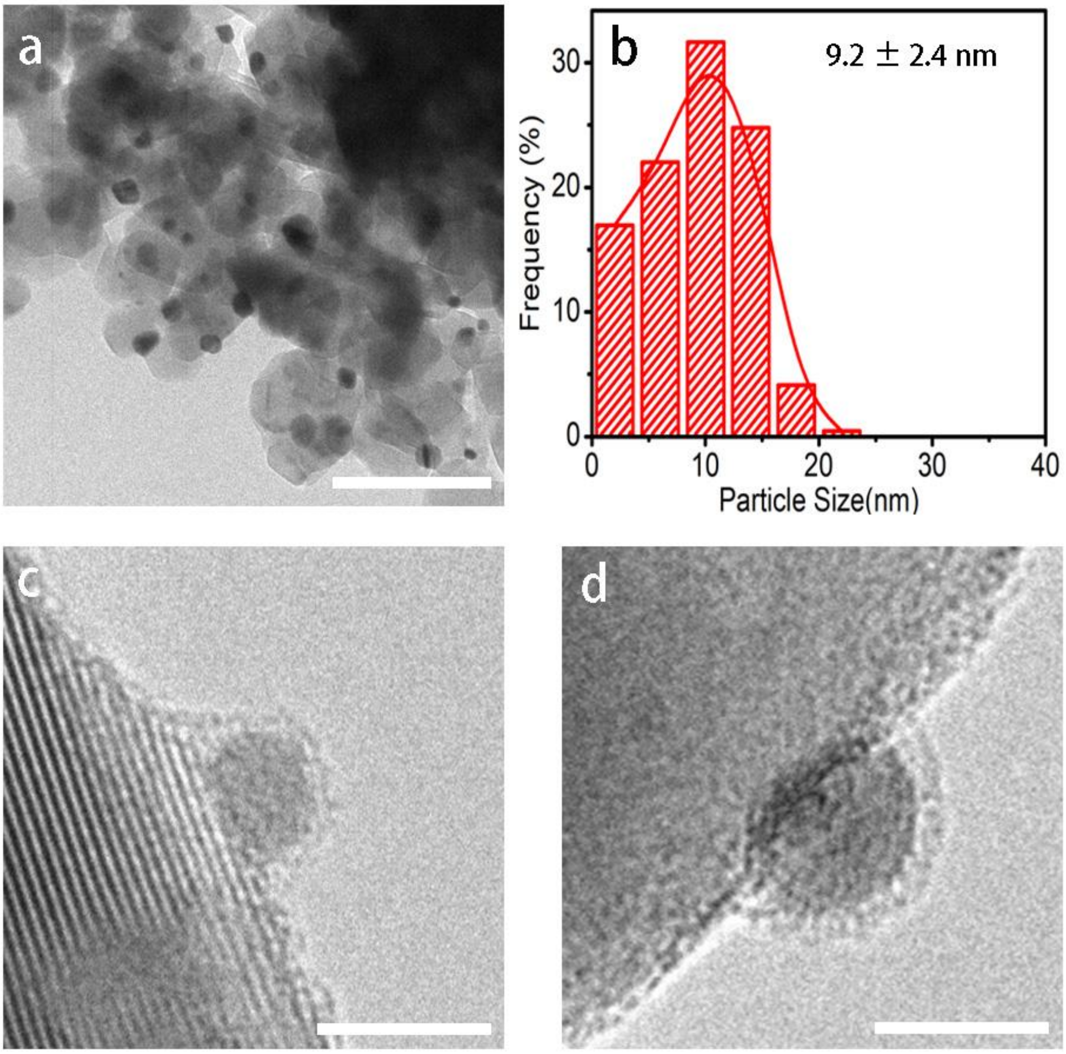

Supplementary Figure 35. Electron microscopy images and particle size distribution of Au/anatase@M-N-800. a TEM image of Au/anatase@M-N-800. **b** Particle size distribution of Au/anatase@M-N-800. **c** and **d** HRTEM images of Au/anatase@M-N-800, in which all Au NPs were encapsulated by TiO_x overlayer. The scale bar in **a** corresponds to 100 nm, and in **c** and **d** corresponds to 5 nm.

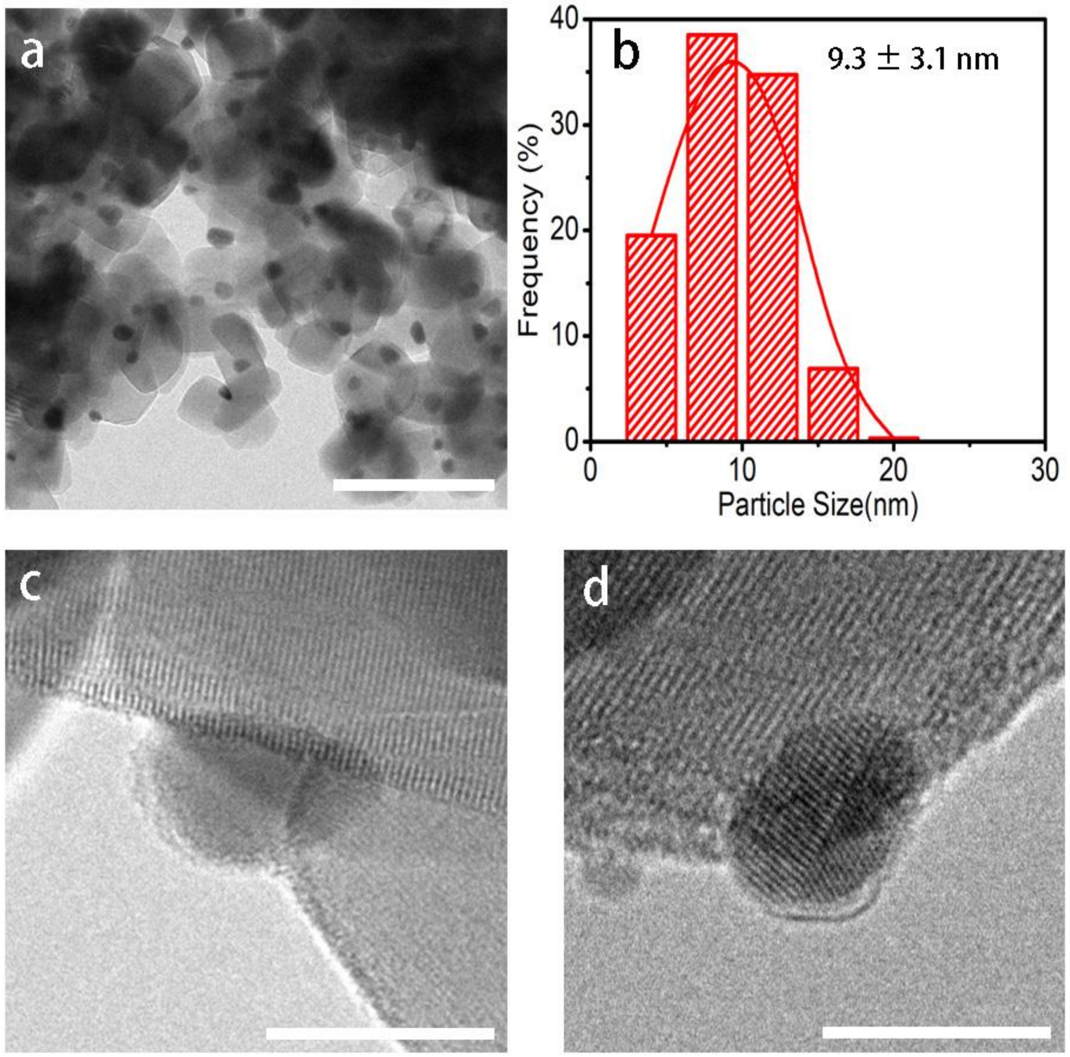

Supplementary Figure 36. Electron microscopy images and particle size distribution of Au/rutile@M-N-800. **a** TEM image of Au/rutile@M-N-800. **b** Particle size distribution of Au/rutile@M-N-800. **c** and **d** HRTEM images of Au/rutile@M-N-800, in which all Au NPs were encapsulated by TiO_x overlayer. The scale bar in **a** corresponds to 100 nm, and in **c** and **d** corresponds to 10 nm.

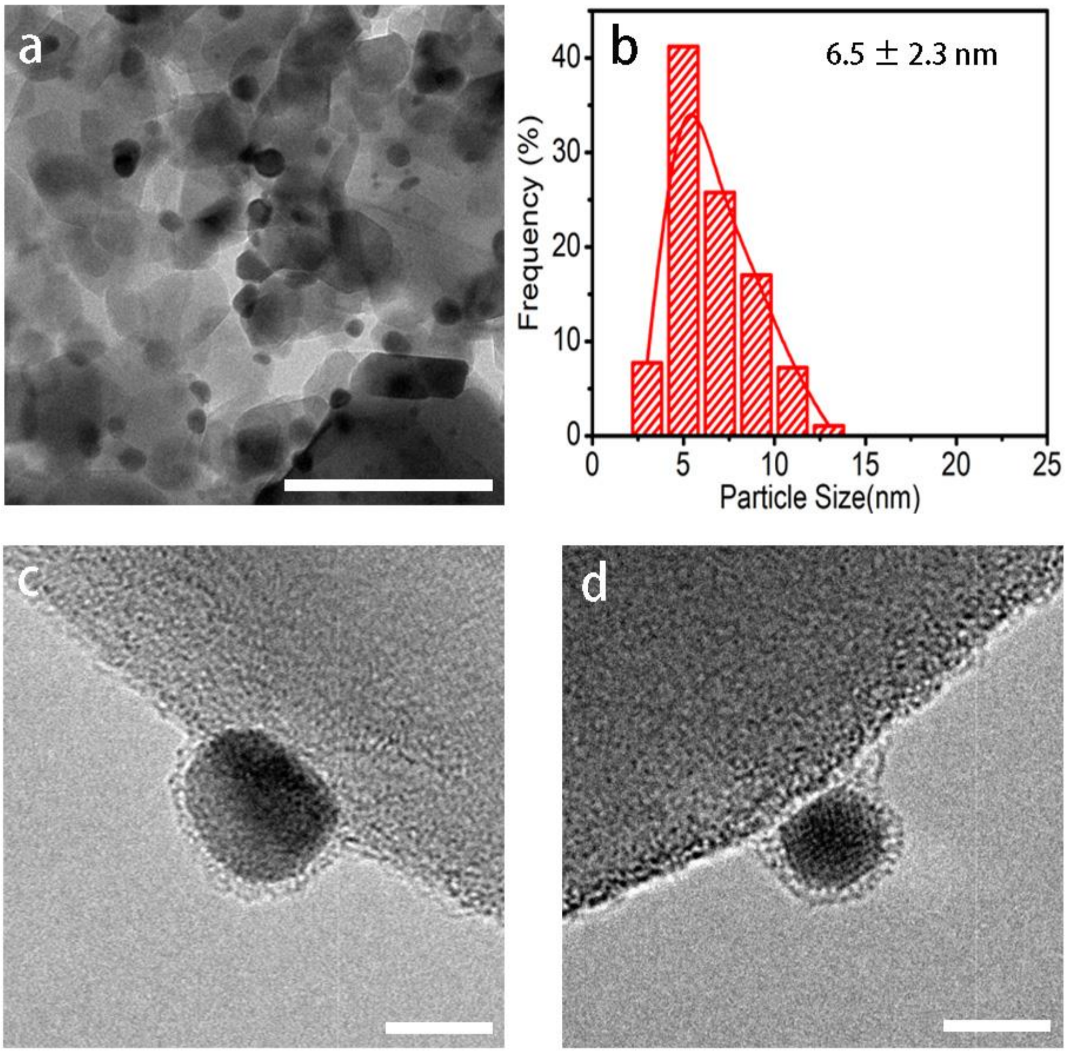

Supplementary Figure 37. Electron microscopy images and particle size distribution of C-Au/TiO_2@M-N-800. **a** TEM image of C-Au/TiO_2@M-N-800. **b** Particle size distribution of C-Au/TiO_2@M-N-800. **c** and **d** HRTEM images of C-Au/TiO_2@M-N-800, in which all Au NPs were encapsulated by TiO_x overlayer. The scale bar in **a** corresponds to 100 nm, and in **c** and **d** corresponds to 5 nm.

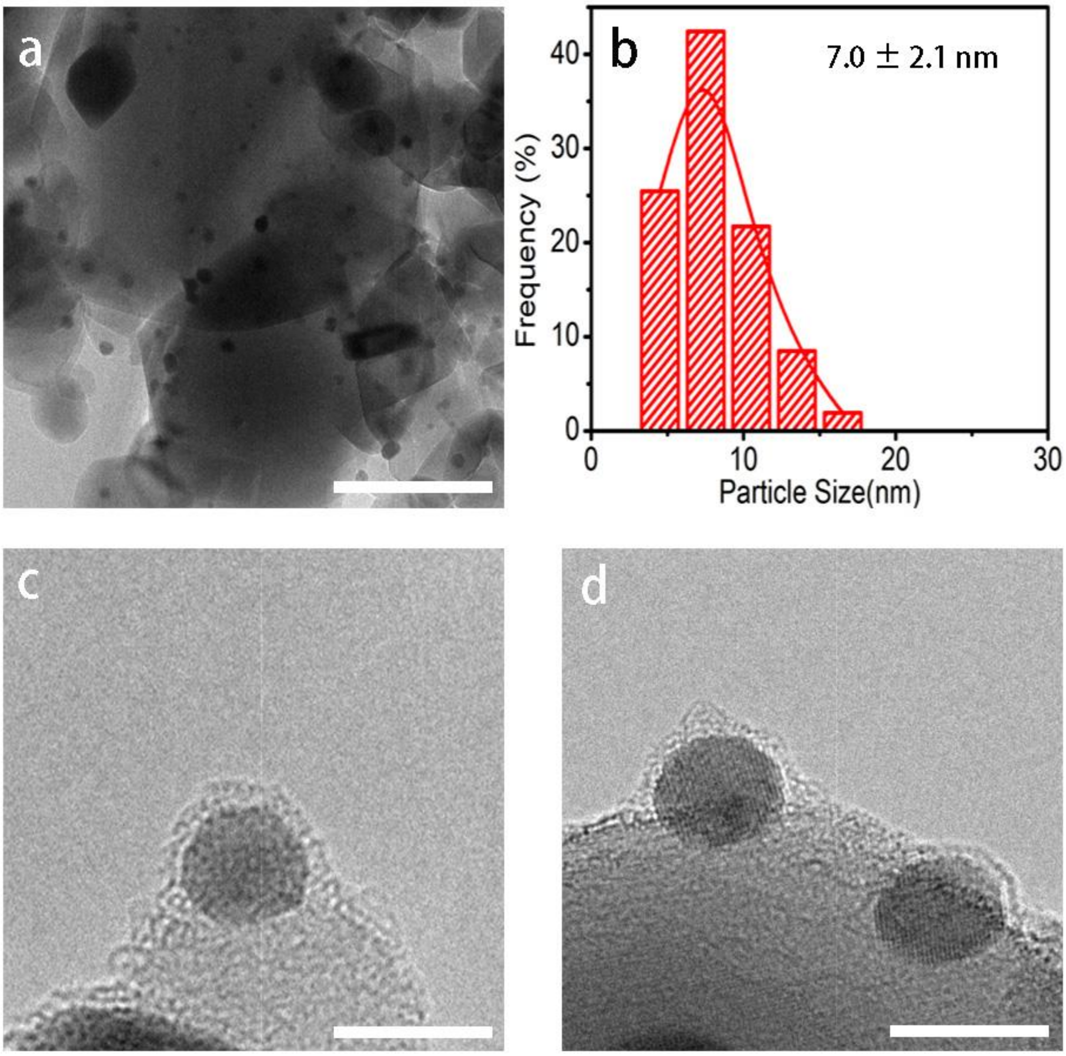

Supplementary Figure 38. Electron microscopy images and particle size distribution of RR2Ti@M-N-800. **a** TEM image of RR2Ti@M-N-800. **b** Particle size distribution of RR2Ti@M-N-800. **c** and **d** HRTEM images of RR2Ti@M-N-800, in which all Au NPs were encapsulated by TiO_x overlayer. The scale bar in **a** corresponds to 100 nm, and in **c** and **d** corresponds to 5 nm.

Supplementary Tables

Supplementary Table 1. Composition ratio of TiO_2 in a series of samples.

Samples	TiO_2	
	Anatase (wt %)	Rutile (wt %)
Au/TiO_2	78	22
Au/TiO_2@M	78	22
Au/TiO_2@M-N	54	36
Au/TiO_2@M-N-800	12	88
Au/TiO_2-800	0	100

Supplementary Table 2. Analysis results of the EXAFS data at Au L_{III}-edge.

**Samples**	**Shell**	**N**	**R(Å)**	**$\sigma^2 \times 10^{-3}$ (Å^2)**	**ΔE_0 (eV)**	**r-factor**
Au foil	Au-Au	12	2.86	7.8	4.5	0.008
	Au-Au	6	4.07	11.8	4.5	
Au/TiO_2	Au-Au1	5.9	2.82	8.52	1.65	0.022
	Au-Au2	0.04	4.00	3.37	1.65	
Au/TiO_2@M	Au-Au1	7	2.83	8.83	3.22	0.017
	Au-Au2	1.4	4.05	9.77	3.22	
Au/TiO_2@M-N	Au-Au1	7.7	2.84	7.93	4	0.018
	Au-Au2	3.5	4.06	11.1	4	
Au/TiO_2@M-N-800	Au-Au1	8.4	2.85	7.81	3.24	0.012
	Au-Au2	4.2	4.06	11.9	3.24	
	Au-Ti	5.6	2.79	73.42	3.74	
Au/TiO_2-800	Au-Au1	9.8	2.86	7.92	4.0	0.006
	Au-Au2	4.2	4.06	10.09	4.0	

N, the coordination number for the absorber-backscatter pair; R, the average distance between absorber and backscatter, σ^2, Debye-Waller factor; ΔE_0, inner potential correction.

Supplementary Table 3. Catalytic performances of Au nanocatalysts compared with the other well-known sintering-resistant Au catalysts reported in literatures.

Catalysts	Loading [a] wt.%	D_{Au} [b] nm	T50 [c] °C	Specific rate ×10^2 mol_{CO} h^{-1} g_{Au}^{-1}	TOF ×10^2 [d] s^{-1}	Test temperature	Ref [e]
Au/TiO_2	3.8	3.5 ±0.8	-2.1	53.6	12	25	This work
Au/TiO_2@M-N-800	3.8	7.5 ±1.6	32	18.6	8.5	25	This work
Au/TiO_2-800	3.8	32.6 ±10.3	245	0.6	1.1	25	This work
Au/TiO_2-H500-SMSI	3.8	3.2 ±0.8	-19.5	66.1	12.9	25	This work
RR2Ti	1.0	4.1 ±2.2	N.D.	153	38.1	25	1
Au/TiO_2-WGC	1.47	3.8 ±0.8	N.D.	179	39.3	25	1
Au/Fe_2O_3-WGC	4.4			22	4.8	27	1
Au/(TiO_2-HAP)-800	2.9	8.6 ±2.3	27	16	8.6	25	1
Au/TiO_2-wcSMSI	0.77	2.0		100	68	25	2
Au/HAP-500	2.8	3.0	18	20	11	25	3
Au/(Fe_2O_3-HAP)-600	3.2	4.5		7.1	1.94	24	4
Au/TiO_2-500	1.0	3.8 ±0.8	-6	27	102		5
Au/ZnO-600	1.94	6.3		18	6.9	100	6
Au/Al_2O_3-700	2.3	2 ±0.8		92	11.1	0	7
Au/(TiO_2-SiO_2)-700	2.2	6.5		45	17.8	64	8
Au/(TiO_2-SiO_2)-800	2.1	3.5 ±1.6		83.3	17.7	20	9
Au/(ZrO_2-SiO_2)-800	5.7	8.7 ±3.9		4.6	2.4	20	9

[a] Au loading.

[b] Particle size of Au NPs.

[c] The temperature required for 50 % CO conversion (T50).

[d] The TOF is calculated according to equation: TOF = $r_{CO*}M_{Au}/D$, D=0.9/d_{Au}, where D is the dispersion of Au and d_{Au} is the diameter of Au NPs.

[e] References.

Supplementary References

1 Tang, H. *et al.* Ultrastable hydroxyapatite/titanium-dioxide-supported gold nanocatalyst with strong metal-support interaction for carbon monoxide oxidation. *Angew. Chem., Int. Ed.* **55**, 10606-10611, (2016).

2 Zhang, J. *et al.* Wet-chemistry strong metal-support interactions in titania-supported Au catalysts. *J. Am. Chem. Soc.* **141**, 2975−2983, (2019).

3 Tang, H. *et al.* Strong metal-support interactions between gold nanoparticles and nonoxides. *J. Am. Chem. Soc.* **138**, 56-59, (2016).

4 Zhao, K., Qiao, B., Wang, J., Zhang, Y. & Zhang, T. A highly active and sintering-resistant Au/FeO_x-hydroxyapatite catalyst for CO oxidation. *Chem. Commun.* **47**, 1779-1781, (2011).

5 Zhan, W. *et al.* A sacrificial coating strategy toward enhancement of metal-support interaction for ultrastable Au nanocatalysts. *J. Am. Chem. Soc.* **138**, 16130-16139, (2016).

6 Liu, J., Qiao, B., Song, Y., Huang, Y. & Liu, J. J. Hetero-epitaxially anchoring Au nanoparticles onto ZnO nanowires for CO oxidation. *Chem. Commun.* **51**, 15332-15335, (2015).

7 Wang, J. *et al.* Thin porous alumina sheets as supports for stabilizing gold nanoparticles. *ACS Nano* **7**, 4902–4910, (2013).

8 Zhu, H., Ma, Z., Overbury, S. H. & Dai, S. Rational design of gold catalysts with enhanced thermal stability: post modification of Au/TiO_2 by amorphous SiO_2 decoration. *Catal. Lett.* **116**, 128-135, (2007).

9 Puértolas, B. *et al.* High-temperature stable gold nanoparticle catalysts for application under severe conditions: the role of TiO_2 nanodomains in structure and activity. *ACS Catal.* **5**, 1078-1086, (2015).
