# Supplementary material for: Ultrastable Au nanoparticles on titania through an encapsulation strategy under oxidative atmosphere
Source: Nat Commun. 2019 Dec 19;10:5790. doi: 10.1038/s41467-019-13755-5 (PMC6923380; doi:10.1038/s41467-019-13755-5)
Supplement: Supplementary file 2 — Peer Review [file 41467_2019_13755_MOESM2_ESM.pdf]

Reviewers' comments:

Reviewer #1 (Remarks to the Author):

In this manuscript, the authors report a method of encapsulating gold nanoparticles (NPs) on a titania support that renders the resultant catalyst resistant to sintering at high-temperatures and in the presence of water vapour, as demonstrated through various CO oxidation tests. This study is the first report of gold encapsulation in an oxidative environment, in contrast to similar studies whereby a reductive treatment was used to induce a classical strong-metal support interaction (SMSI).

Regarding the impact of the work, the stabilisation of Au nanoparticles is of great interest in the field of heterogeneous catalysis. However, similar studies have been recently reported, even by some of the same authors [1]. In [1], very similar studies were carried out (characterisation methods and catalyst tests), albeit with a different route to NP encapsulation. The novelty of the current work is therefore diminished and is simply that melamine is used as a sacrificial agent, which facilitates encapsulation in an oxidative environment. This in itself is interesting, but the understanding of this aspect of the work is limited. Did the authors try alternative agents to melamine or is the effect unique to this molecule and over Au on titania? If these questions were answered, it could broaden the appeal of the work and clearly show that it is of high impact.

On the other hand, the importance encapsulating Au in an oxidising environment has not been set out in the manuscript in its current form and the catalyst does not outperform the state-of-the-art, but is amongst the better reported ones (as shown in Supplementary table 3). Additionally, the encapsulated nanoparticles are over 7 nm, which is rather large compared to other encapsulated NPs (5.2 nm in [1]) and what would be considered to be in the region of highly active, efficient Au NPs (< ca. 5 nm).

In order to enhance the impact of the paper the authors should compare their encapsulated Au/TiO2 catalyst (denoted Au/TiO2@M-N-800) with the classical SMSI-derived Au/TiO2 catalyst reported in [1] and show why the melamine method is preferable.

From a technical perspective, there are several strengths to this manuscript, including the detailed microscopy of the catalyst after various treatments, which clearly shows the subsequent growth and eventual encapsulation of the supported nanoparticles. However, there are also concerns/queries with some aspects of the interpretation of data and methods used. These should be clarified/amended where necessary:

• Regarding in situ DRIFTS: Inferences are made based on the intensity of CO bands in different spectra. It isn't clear how the authors accounted for possible differences in sample mass/instrument settings that could give rise to different intensities. The manuscript also doesn't make it clear if the recorded spectra are in the presence of gas-phase CO (the experimental suggests that they are, but gas-phase CO bands are not assigned). The broad feature between 2200 and 2150 cm-1 clearly resembles part of the characteristic gas-phase CO band (the other part would be found between ~2150 and 2100 cm-1). Gas-phase CO seems to dominate the

spectra of Au/TiO2@M-N-800 and so the assignment of the feature at 2116 cm-1 to a "more positive charge on the surface of Au species" is not persuasive.

• Shifts in the binding energy of Au0 in the Au 4f spectrum of each catalyst is used to infer changes in the valence state of the catalyst surface. However, in many cases the binding energy shifts are too small to be meaningfully ascribed to changes in the catalyst. Certainly differences of 0.02 eV are much too small to be significant and are more likely to be due to instrumental/calibration errors.

• The difference in binding energy between Au/TiO2@M-N-800 and Au/TiO2-800 was ascribed to a difference in interaction between Au and TiO2 in the catalysts. However, the considerable difference in NP size may also account for this. The binding energy for Au0 is size-dependent in supported catalysts and therefore the authors should consider how this may affect the composition of the Au 4f spectra. It may be that the species ascribed to Au+ actually originate from small Au NPs. Goodman and co-workers demonstrated this on Au/TiO2 model catalysts [2].

• The carbon region of the samples could also be instructive to characterise the overlayer formed on the NPs. The authors should comment on or included this data.

• The CO testing data undoubtedly shows that the Au/TiO2@M-N-800 is more active than a comparable Au/TiO2 catalyst without melamine, but what about the Au-TiO2 with classical SMSI effect reported in [1]? This is the most pertinent comparison to draw.

• Regarding the TOF calculations: Can the authors explain how they calculated the dispersion of Au? Additionally, were TOF measurements made at low conversion, ie. in a kinetic regime? The TOF measurements of Au/TiO2 and Au/TiO2@M-N-800 look like they were made using the data at 25 oC in fig 5a. However, the Au/TiO2 is close to 100% conversion and the rate may be mass-transfer limited. The authors should clearly set out under which conditions (including CO conversion) the TOF measurements were calculated and demonstrate that the reaction was under kinetic control.

• In fig 5b they show consecutive cycles up to 800 oC. Unfortunately the conversion quickly reaches 100 % and the information between 0 and 100 C is not very visible. It looks as though the activity at 25 oC decreases from ca. 40% in the first cycle to ca. 20% in the tenth cycle, yet the authors conclude the catalyst exhibited 'prominent' stability during cycles.

• In fig 5c the authors show stable conversion over ten days in a stream of CO, H2O, O2, propene and toluene. Have the authors considered that the water-gas shift reaction might be in play? At this temperature and over this catalyst it is reasonable to expect Au/TiO2 to be active for this reaction, and therefore there will be a thermodynamic equilibrium to consider. If the equilibrium limit of CO conversion is around 90%, the catalyst may well be deactivating during this reaction, but the experimental conditions mean it is difficult to observe. That said, the post-reaction particle size distribution is almost identical.

• The authors write on line 144-145 that the encapsulation state leads to a structural rearrangement of the TiO2 support in the neighbourhood of the metal particle. This is quite vague so can the authors elaborate on what they mean by this? Does it refer to the support 'beneath' the nanoparticle or the support that has encapsulated it? How big is the 'neighbourhood'?

• Can the authors comment on why the activity of the catalysts appeared to increase over time?

• Can the authors comment on a) what percentage of Au NPs were encapsulated and b) if the smaller Au NPs were equally as likely as the larger ones to be encapsulated?

In conclusion, the manuscript reports interesting results with the potential to influence thinking in the field of heterogeneous catalysis - if they can demonstrate that this method of encapsulation is superior to alternative techniques.

References
[1]. Classical strong metal–support interactions between gold nanoparticles and titanium dioxide" Tang et al., Sci. Adv. 2017; 3: e1700231.
[2] Goodman, Catalysis by supported gold nanoclusters in Dekker Encyclopedia of Nanoscience and Nanotechnology, Pages 611-620.

Reviewer #2 (Remarks to the Author):

Wang and co-workers reported the construction of SMSI on the as-prepared Au/TiO2 catalysts using a melamine induced TiOx layer under the calcination treatment. The formed TiOx layer could stabilize the Au nanoparticles from sintering in the oxidation of CO, which is used here as a model reaction. The results are interesting and reasonable. In addition, the method was extendable, which can be used for the formation of SMSI on the commercial catalyst. This manuscript might be acceptable for publication in nat. comm., but major revision is still necessary to address the following issues.

1. In the classical SMSI from the high-temperature reduction treatment, a de-SMSI usually occurred in the water-treatment or oxidative conditions, where the oxide layer was removed under the given conditions. However, this work emphasized that the layer was stable even after the high-temperature calcination, and the reaction conditions with water feed. What is the reason for the oxide layer stabilization?
2. The TOF of the Au catalysts have been provided, it is obtained on the basis of accessible Au? A comparison with the other Au catalysts on the catalytic activity should be addressed, for example, comparison with the Au/TiO2 with wet-chemistry SMSI (Wang and Xiao, JACS), Au/HAP with oxidative SMSI (Wang and Zhang, JACS), Au/TiO2 with classical SMSI (Qiao and Wang, Sci Adv).
3. More evidences should be provided on the formation of SMSI. The author talked about the formation of electronic interaction between Au and TiOx led by the melamine. More details should be provided. Is the melamine completely burned after the calcination? The melamine might be continuous decomposed during the calcination treatment at higher temperature. Does this molecule work at low temperature (because it is decomposed at high temperature)? The composition of the oxide layer, pure TiOx or carbon-containing TiOx, should be studied.
4. The RESULTS section started with the characterization data. A brief introduction of the synthesis procedures should benefit the readers to understand the process.
5. Some other important references on the sinter-resistant catalysts should be added in the revised manuscript, such as Nature Catal. 2018, 1, 540-546; Science, 2012, 335, 1205; Angew, 2012, 51, 5929; 2017, 56, 9747-9751.

Reviewers' comments:

Reviewer #1 (Remarks to the Author):

In this manuscript, the authors report a method of encapsulating gold nanoparticles (NPs) on a titania support that renders the resultant catalyst resistant to sintering at high-temperatures and in the presence of water vapour, as demonstrated through various CO oxidation tests. This study is the first report of gold encapsulation in an oxidative environment, in contrast to similar studies whereby a reductive treatment was used to induce a classical strong-metal support interaction (SMSI).

1. Regarding the impact of the work, the stabilisation of Au nanoparticles is of great interest in the field of heterogeneous catalysis. However, similar studies have been recently reported, even by some of the same authors [1]. In [1], very similar studies were carried out (characterisation methods and catalyst tests), albeit with a different route to NP encapsulation. The novelty of the current work is therefore diminished and is simply that melamine is used as a sacrificial agent, which facilitates encapsulation in an oxidative environment. This in itself is interesting, but the understanding of this aspect of the work is limited. Did the authors try alternative agents to melamine or is the effect unique to this molecule and over Au on titania? If these questions were answered, it could broaden the appeal of the work and clearly show that it is of high impact.

Response: Thank you for your constructive and encouraging comments. We agree with the reviewer that there are some similarity between this work and the classical SMSI in Au/TiO_2. However, it does not mean that the novelty of this work is diminished and the reasons are as follows. Firstly, the classical SMSI between Au and TiO_2 occurs in reductive condition at high temperature (＞500 ^oC), which is reversible under further pretreatment in oxidation environment, leading to invalid effect on the stability and catalytic property of the Au especially for high temperature (＞400 ^oC) oxidation reaction. While in this work the encapsulation induced by melamine occurs under oxidative condition, to the best of our knowledge, it is the first time to report that encapsulation of Au by TiO_2 under oxidative condition. Secondly, the formed TiO_x overlayer does not retreat when further calcination in air at 400 to 600 ^oC, which is totally different from the classical SMSI. Furthermore, we have demonstrated that this kind of TiO_x overlayer still existed after simulated CO emission control reactions at 400 ^oC for 10 days even in the presence of water, exhibiting an excellent activity and durability, which providing a new avenue to develop sintering-resistant gold catalyst with high activity and stability. Meanwhile, inspired by the reviewer 2#'s question 1, we speculate that it is the formation condition of the encapsulation that determines the property of TiO_x overlayer, although the formation mechanism of this phenomenon is still unclear at present. The underlying reason and the nature driving force for this encapsulation may be to minimize the surface energy. And we are now trying to use other organic reagent to replace melamine and the work is in progress. It is worth noting that we have found the similar encapsulation in Pt/TiO_2 using melamine, which was also totally different from that of classical SMSI between Pt and TiO_2, termed by Tauster et al. in the late 1970s. Therefore, this strategy may also be extended to platinum group metals and titania, providing a universal

way to design sintering-resistant supported catalysts.

2. On the other hand, the importance encapsulating Au in an oxidising environment has not been set out in the manuscript in its current form and the catalyst does not outperform the state-of-the-art, but is amongst the better reported ones (as shown in Supplementary table 3). Additionally, the encapsulated nanoparticles are over 7 nm, which is rather large compared to other encapsulated NPs (5.2 nm in [1]) and what would be considered to be in the region of highly active, efficient Au NPs (< ca. 5 nm).

Response: We appreciate the reviewer's professional comments and suggestions. The reviewer is right and we have added the importance of encapsulating Au in an oxidizing environment in the revised manuscript as "To the best of our knowledge, it is the first time to report that Au NPs can be encapsulated by titania under oxidative atmosphere at high temperature, in contrast with condition required for the classical SMSI in Au/TiO_2. Although the resultant Au/TiO_2@M-N-800 does not outperform the state-of-the-art, it is still comparable to the Au/TiO_2-HAP-800 and Au/TiO_2-SiO_2-800 catalysts, which are the most excellent sintering resistance catalyst up to now." While the encapsulated Au NPs are not all larger than the encapsulated Au NPs in classical SMSI (Sci Adv 2017, 3, e1700231) and the smaller Au NPs can also be encapsulated similarly to that of the larger ones (Please see Figure R1 or Supplementary Fig. 3 and Fig. 22). However, it is difficult to observe the morphology and encapsulation of gold when the Au NPs particle size is less than 2 nm. We have added these data to the revised manuscript and supplementary information.

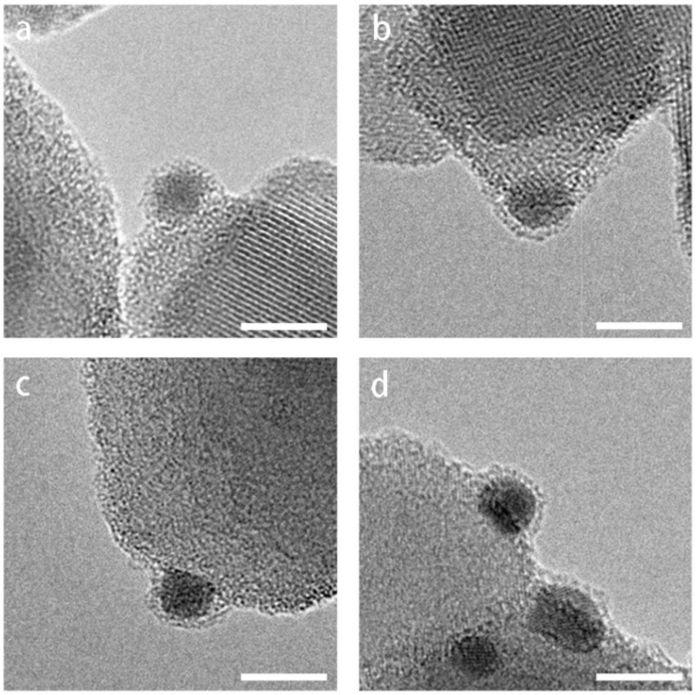

Figure R1. HRTEM analysis. a, b, c and **d** HRTEM images of Au/TiO_2@M-N-800, in which all the small Au NPs were encapsulated by TiO_x overlayer. The scale bars are all 5 nm.

3. In order to enhance the impact of the paper the authors should compare their encapsulated Au/TiO2 catalyst (denoted Au/TiO2@M-N-800) with the classical SMSI-derived Au/TiO2 catalyst

reported in [1] and show why the melamine method is preferable.

Response: This is a good question and suggestion, thank you! We apologize for a lack of the comparison of the activity between the Au/TiO_2@M-N-800 and the Au/TiO_2 in classical SMSI state in our original manuscript, which is presented as follows. Figure R2 showed the activity of Au/TiO_2 in classical SMSI state before and after calcination. The T50 of Au/TiO_2-H500 and Au/TiO_2-H500-O400 were -19.5 and 16.5 ^{o}C with an average particle size about 3.2 and 4.2 nm, respectively (Figure R2-4 or Supplementary Fig. 13-15). HRTEM results showed that Au NPs were encapsulated with a TiO_x overlayer in Au/TiO_2-H500, which retreated under further pretreatment under oxidation condition, consistent with our previously report (Sci Adv 2017, 3, e1700231). It should be noted that the TiO_x overlayer in classical SMSI was much thinner than the overlayer induced by melamine. After calcination at 800 ^{o}C in air for 3 h, the T50 of Au/TiO_2-H500-800 and Au/TiO_2-H500-O400-800 were 191 and 168 ^{o}C and the particle size were 28.6 and 27.2 nm, respectively, in which no overlayer was observed and Au NPs sintered seriously (Figure R5 & 6 or Supplementary Fig. 16 & Fig. 17). Therefore, the overlayer formed in the reduction condition retreated under oxidation condition (classical SMSI) may account for the sintering of the Au NPs in Au/TiO_2-H500-800, leading to invalid effect on the stability and catalytic property of the gold. And the T50 of Au/TiO_2@M-N-800 was much lower than that of Au/TiO_2-H500-800, exhibiting the excellent sintering-resistant ability and superiority of Au/TiO_2@M-N-800 compared with Au/TiO_2-H500-800. The synthesis procedure of the above catalysts and relevant discussion have been added to the corresponding section in the revised manuscript and supplementary information.

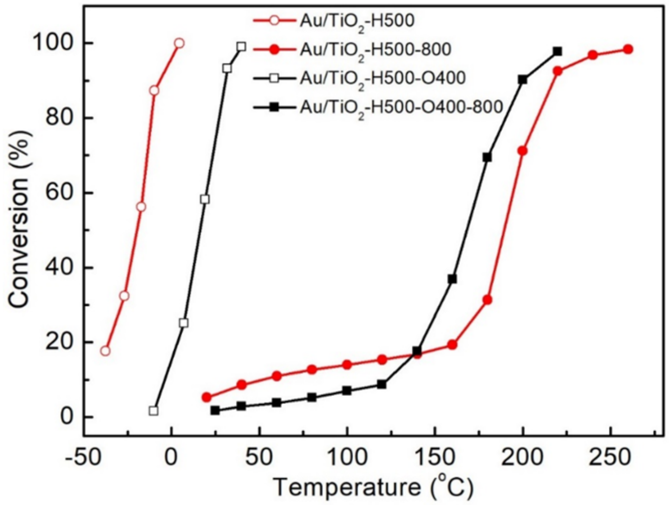

Figure R2. Evaluation of Au/TiO_2 nanocatalysts in CO oxidation. CO oxidation curves of Au/TiO_2-H500, Au/TiO_2-H500-O400, Au/TiO_2-H500-800 and Au/TiO_2-H500-O400-800 with a feed gas comprising 1 vol% CO/ 1 vol% O_2/ 98 vol% He at 33.3 mL min^{-1}.

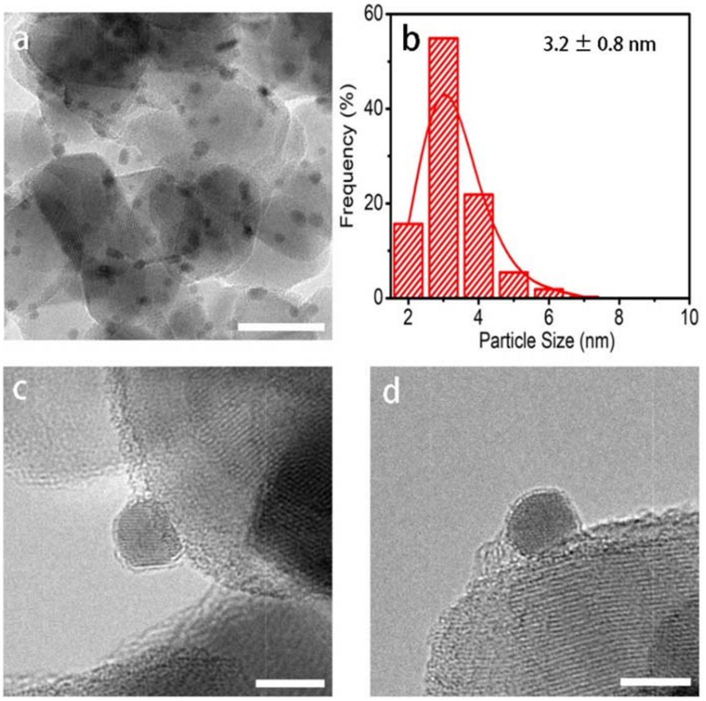

Figure R3. Electroscopy microscpoy images of Au/TiO_2-H500. **a** TEM images of Au/TiO_2-H500. **b** Particle size distribution of Au/TiO_2-H500. **c** and **d** HRTEM images of Au/TiO_2-H500, in which Au NPs were covered by TiO_x overlayer similar to the reference [1]. The scale bar in **a** corresponds to 20 nm, and in **c** and **d** corresponds to 5 nm.

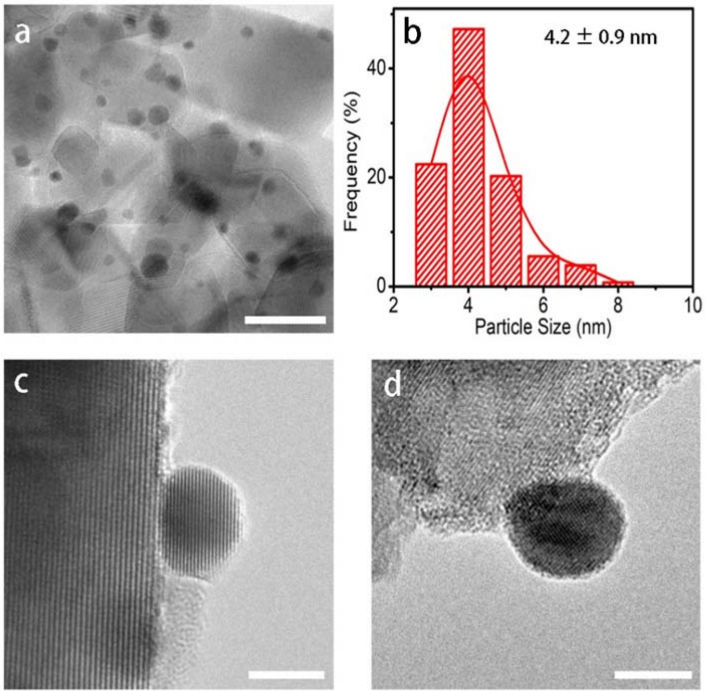

Figure R4. Electroscopy microscpoy images of Au/TiO_2-H500-O400. **a** TEM images of Au/TiO_2-H500-O400. **b** Particle size distribution of Au/TiO_2-H500-O400. **c** and **d** HRTEM images of

Au/TiO_2-H500-O400, in which the overlayer retreated and Au NPs were bare. The scale bar in **a** corresponds to 20 nm, and in **c** and **d** corresponds to 5 nm.

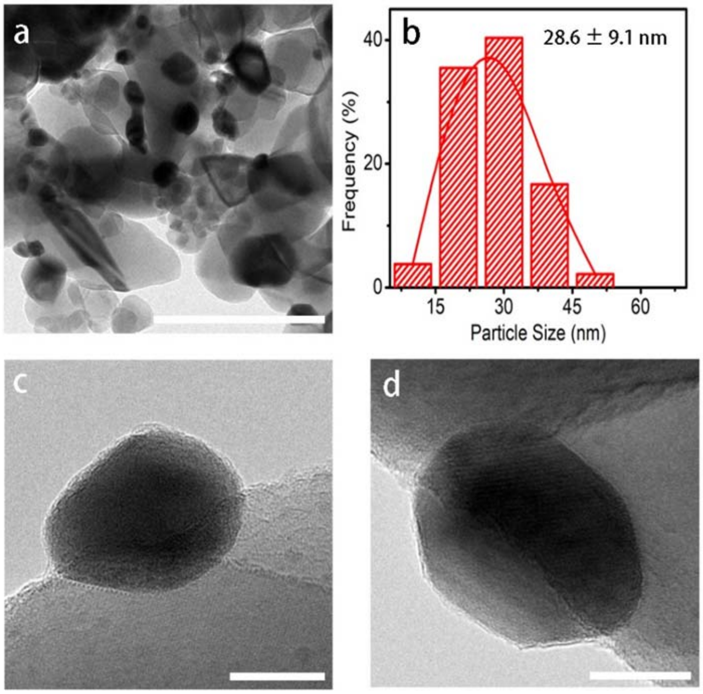

Figure R5. Electroscopy microscpoy images of Au/TiO_2-H500-800. **a** TEM images of Au/TiO_2-H500-800. **b** Particle size distribution of Au/TiO_2-H500-800. **c** and **d** HRTEM images of Au/TiO_2-H500-800, in which Au NPs sintered seriously. The scale bar in **a** corresponds to 200 nm, and in **c** and **d** corresponds to 10 nm.

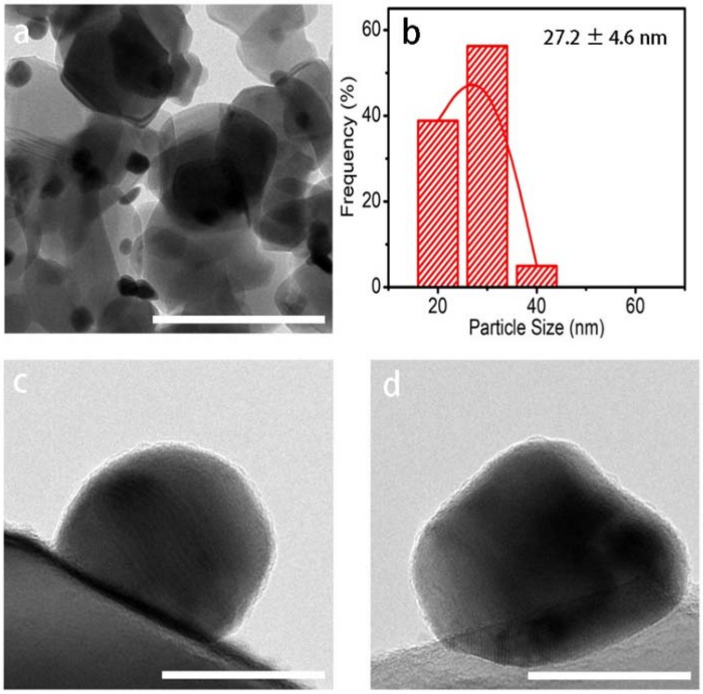

Figure R6. Electroscopy microscpoy images of Au/TiO_2-H500-O400-800. **a** TEM images of Au/TiO_2-H500-O400-800. **b** Particle size distribution of Au/TiO_2-H500-O400-800. **c** and **d** HRTEM images of Au/TiO_2-H500-O400-800, in which Au NPs sintered seriously. The scale bar in **a** corresponds to 200 nm, and in **c** and **d** corresponds to 20 nm.

4. From a technical perspective, there are several strengths to this manuscript, including the detailed microscopy of the catalyst after various treatments, which clearly shows the subsequent growth and eventual encapsulation of the supported nanoparticles. However, there are also concerns/queries with some aspects of the interpretation of data and methods used. These should be clarified/amended where necessary:

• Regarding in situ DRIFTS: Inferences are made based on the intensity of CO bands in different spectra. It isn't clear how the authors accounted for possible differences in sample mass/instrument settings that could give rise to different intensities. The manuscript also doesn't make it clear if the recorded spectra are in the presence of gas-phase CO (the experimental suggests that they are, but gas-phase CO bands are not assigned). The broad feature between 2200 and 2150 cm-1 clearly resembles part of the characteristic gas-phase CO band (the other part would be found between ~2150 and 2100 cm-1). Gas-phase CO seems to dominate the spectra of Au/TiO2@M-N-800 and so the assignment of the feature at 2116 cm-1 to a "more positive charge on the surface of Au species" is not persuasive.

Response: Thank you for your nice comments and good questions. Actually, all the data was obtained on different catalysts with equal mass in the same instrument under the exactly same condition. We apologized for a lack of clarity in describing the measurement condition of DRIFTS in characterizations section of the original manuscript and indicating the gaseous CO peak. The measurement condition of DRIFTS was that: "before CO adsorption, the samples were pretreated for 1 h with He at 120 ^{o}C, then cooled to room temperature. The background spectrum was collected in following He and then the gas (3% CO/He) was introduced into the reaction cell at a total flow rate of 33.3 mL min^{-1}. The spectra were recorded until the peak intensities were steady." Therefore, the reviewer is right that the DRIFTS was collected in the presence of gas-phase CO, the peak of which was 2174 cm^{-1} between 2200 and 2150 cm^{-1}. Although we don't believe that the peak (2116 cm^{-1}) would be the gas-phase CO as CO gaseous bands adsorption were 2174 and 2120 cm^{-1} (Angew. Chem., Int. Ed. 2016, 55, 10606-10611 and J. Am. Chem. Soc. 2016, 138, 56-59), the possibility could be completely excluded according to the mentioned references. To demonstrate this, we further purged the sample with He after CO adsorption saturation. As shown in Figure R7a and R7b in this response, under He purge the 2174 cm^{-1} band decreased rapidly after 30 s and disappeared completely within 2 min while 2116 cm^{-1} still existed, suggesting that this band is the peak of CO adsorption on $Au^{\delta+}$ as CO-$Au^{\delta+}$ is quite stable (more stable than or at least similar to that of CO on Au^{o}) (Chem. Ing. Tech. 2007, 79, 795-806). Furthermore, the blank test was also carried out and reported in the revised manuscript. The blank test procedure was inserted into the Characterization: "The blank test was carried out following the exactly same procedure just without catalyst." Figure R7c and R7d showed that only gaseous CO bands 2174 and 2120 cm^{-1} was detected and all of them disappeared completed within 2 min when purge with He, which was different from that of Au/TiO_2@M-N-800. Therefore, 2116 cm^{-1} should be attributed to CO adsorption on $Au^{\delta+}$ although it was indeed weak due to the encapsulation of gold nanoparticles by TiO_x (J. Am. Chem. Soc. 2012, 134, 10251-10258 and Sci

Adv 2017, 3, e1700231). Moreover, this behavior is exactly similar to that of Au/TiO_2 and Au/TiO_2@M-N (Figure R7e and R7f). From these data and analysis, we conclude that the 2174 cm^{-1} band should be ascribed to the gaseous CO and 2116 cm^{-1} band was due to CO adsorption on $Au^{\delta+}$ instead of gaseous CO. We have added these results and corresponding discussion in to the revised manuscript and supplementary information (Supplementary Fig. 11).

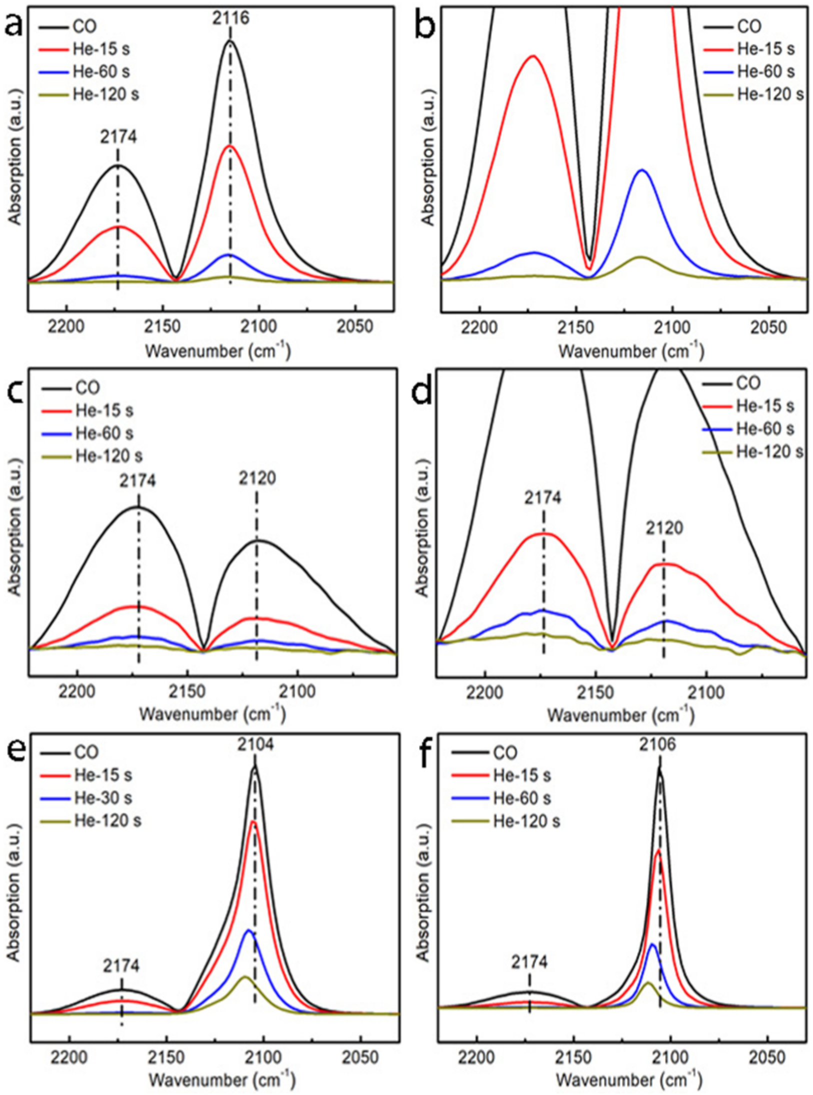

Figure R7. DRIFTS analysis. The *in situ* DRIFT spectra of CO desorption on **a** Au/TiO_2@M-N-800, **b** The enlargement of **a**, **c** The blank test, **d** The enlargement of **c**, **e** Au/TiO_2 and **f** Au/TiO_2@M-N under the purge of helium at room temperature.

5 • Shifts in the binding energy of Au0 in the Au 4f spectrum of each catalyst is used to infer changes in the valence state of the catalyst surface. However, in many cases the binding energy shifts are too small to be meaningfully ascribed to changes in the catalyst. Certainly differences of 0.02 eV are much too small to be significant and are more likely to be due to instrumental/calibration errors.

Response: Yes, the reviewer is right. Thanks very much for your constructive suggestion. To

investigate whether there was instrumental/calibration errors, XPS measurement was carried out once again and almost similar results were obtained, however. And after having a discussion with the some experts in XPS, it was found that the instrumental/calibration errors cannot be excluded completely. Therefore, we have removed the Au 4f spectra from the revised manuscript from the perspective of strict analysis. Thanks very much.

6 • The difference in binding energy between Au/TiO2@M-N-800 and Au/TiO2-800 was ascribed to a difference in interaction between Au and TiO2 in the catalysts. However, the considerable difference in NP size may also account for this. The binding energy for Au0 is size-dependent in supported catalysts and therefore the authors should consider how this may affect the composition of the Au 4f spectra. It may be that the species ascribed to Au+ actually originate from small Au NPs. Goodman and co-workers demonstrated this on Au/TiO2 model catalysts [2].
Response: This is a good question. Actually, the core level binding energy in reference [2] is due to different the electronic structure between small metal clusters (less than 2 nm) and bulk metal (~5 nm) that results in the change of binding energy. And the shift of core-level binding energy usually depends on the nature of the supporting substrate. For weakly interacting substrates such as carbon, the shifts are to higher the core-level binding energy shifts for clusters relative to bulk metal, which was caused by an increase in the *d* electron count with increasing size due to (*s*, *p*)-*d* rehybridization or intra-atomic charge transfer. For substrate with localized *p* or *d* orbital with binding energy overlapping those of cluster *d* orbitals, the cluster binding energy is usually shifted to lower binding energy for cluster relative to bulk metal, which was thought from simple molecular-orbital arguments. Therefore, this kind of binding energy shift should refer to the difference between small metal clusters and bulk metal. However, the particle size of Au NPs in this work are all larger than 3.5 nm, such as 7.5 nm in Au/TiO_2@M-N-800 and 32.6 nm in Au/TiO_2-800, which all belong to bulk metal not small metal clusters and therefore the electronic structure among them has no such a big difference when compared with that of small metal clusters (less than 2 nm) and bulk metal (~5 nm). Therefore, the binding energy shift due to size change from small metal clusters to bulk metal can be excluded and it is believed that the Au^+ has no relationship with the small Au NPs. Thanks very much.

7 • The carbon region of the samples could also be instructive to characterise the overlayer formed on the NPs. The authors should comment on or included this data.
Response: Thanks for your constructive suggestion. To identify whether there was carbon species in the overlayer, electron energy loss spectroscopy (EELS) of Au/TiO_2@M-N-800 was characterized once more and the results were depicted in Figure R8 and R9 (or Supplementary Fig. 7 and Fig. 8). As we can see, no C or N signal was observed in the overlayer region, demonstrating the composition of overlayer was pure TiO_x. (Note: The signal of C and N in EELS are located at 280 and 400 eV, respectively). Moreover, N1s XPS spectra in Au/TiO_2@M definitely demonstrated that melamine was adsorbed on the Au/TiO_2 (Supplementary Fig. 9). And a decrease in the intensity of N1s XPS in Au/TiO_2@M-N may be ascribed to the carbonization of melamine and no N1s XPS signal in Au/TiO_2@M-N-800 was observed which demonstrated melamine had decomposed completely. On the other hand, it is very difficult to identify C species with scanning transmission electron microscopy-energy-dispersive X-ray spectroscopy (STEM-EDX) because of great inaccuracy. And we have added these data with corresponding discussion on to

the revised manuscript and supplementary information.

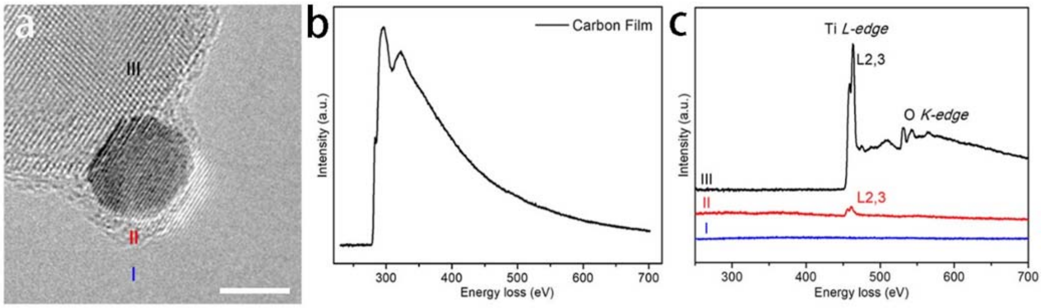

Figure R8. Electron energy loss spectroscopy analysis. a HRTEM images of Au/TiO_2@M-N-800. **b** EELS spectra of carbon film. **c** EELS spectra of Au/TiO_2@M-N-800 from 250 to 700 eV. The spectra was background-subtracted. The scale bar in **a** corresponds to 5 nm.

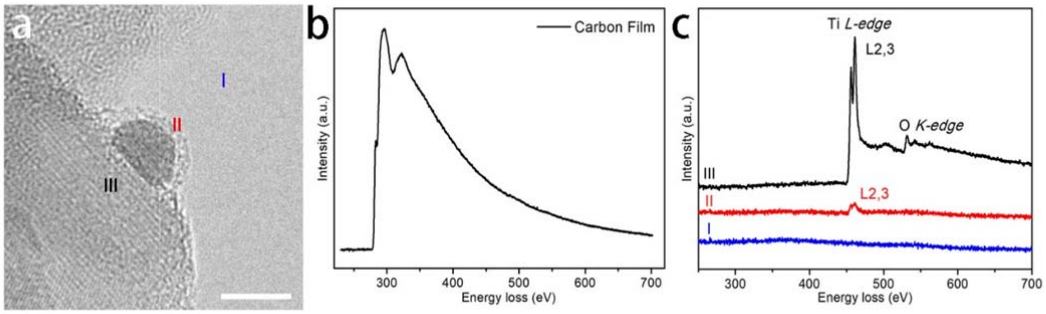

Figure R9. Electron energy loss spectroscopy analysis. a HRTEM images of Au/TiO_2@M-N-800. **B** EELS spectra of carbon film. **c** EELS spectra of Au/TiO_2@M-N-800 from 250 to 700 eV. The spectra was background-subtracted. The scale bar in **a** corresponds to 5 nm.

8 • The CO testing data undoubtedly shows that the Au/TiO2@M-N-800 is more active than a comparable Au/TiO2 catalyst without melamine, but what about the Au-TiO2 with classical SMSI effect reported in [1]? This is the most pertinent comparison to draw.

Response: Thanks for your constructive question. Actually, this question is almost similar to question 3. As we discussed before, Au NPs sintered seriously in Au/TiO_2-H500 when calcination at 800 ^oC because of the retreat of the TiO_x overlayer under oxidative atmosphere (classical SMSI). However, the overlayer in Au/TiO_2@M-N-800 was formed under oxidative atmosphere at 800 ^oC which did not retreat under further calcination in air, indicating high superiority compared with classical SMSI for high temperature oxidation reaction. These discussions are now included in the revised manuscript.

9 • Regarding the TOF calculations: Can the authors explain how they calculated the dispersion of Au? Additionally, were TOF measurements made at low conversion, ie. in a kinetic regime? The TOF measurements of Au/TiO2 and Au/TiO2@M-N-800 look like they were made using the data at 25 oC in fig 5a. However, the Au/TiO2 is close to 100% conversion and the rate may be mass-transfer limited. The authors should clearly set out under which conditions (including CO conversion) the TOF measurements were calculated and demonstrate that the reaction was

under kinetic control.

Response: Thank you for your questions and comments. We apologize for not making this point clearer in the original manuscript. The dispersion of Au was calculated based on the equation $D=0.9/d_{Au}$, where D is the dispersion of Au and d_{Au} is the diameter of Au NPs. Specific reaction rates and turn over frequency (TOF) were evaluated by changing the weight of catalysts from 100 mg to 2 mg to guarantee that CO conversion was below 15%. For each run, the CO conversion was averaged at the steady state and the TOF is calculated according to equation: $TOF = r_{CO}M_{Au}/D$, where r_{CO} is the specific reaction rates and M_{Au} was molar weight of Au. We also apologized that the T50 of Au/TiO_2@M-N-800 catalyst was 32 ^oC and we have corrected this in the revised manuscript.

10 • In fig 5b they show consecutive cycles up to 800 oC. Unfortunately the conversion quickly reaches 100 % and the information between 0 and 100 C is not very visible. It looks as though the activity at 25 oC decreases from ca. 40% in the first cycle to ca. 20% in the tenth cycle, yet the authors conclude the catalyst exhibited 'prominent' stability during cycles.

Response: Yes, the reviewer is right. Thank you for kindly pointing out this mistake, we have corrected these in the revised manuscript.

11 • In fig 5c the authors show stable conversion over ten days in a stream of CO, H2O, O2, propene and toluene. Have the authors considered that the water-gas shift reaction might be in play? At this temperature and over this catalyst it is reasonable to expect Au/TiO2 to be active for this reaction, and therefore there will be a thermodynamic equilibrium to consider. If the equilibrium limit of CO conversion is around 90%, the catalyst may well be deactivating during this reaction, but the experimental conditions mean it is difficult to observe. That said, the post-reaction particle size distribution is almost identical.

Response: Thanks for your good suggestion. We totally agree with the reviewer and therefore water-gas shift (WGS) reaction was performed with Au/TiO_2@M-N-800 catalyst at 500 ^oC with space velocity of 18 L g_{cat}^{-1} h^{-1}. As expected, the conversion almost kept at 45 % during the 90 h test, further certifying the excellent stability of Au/TiO_2@M-N-800 catalyst (Figure R10 or Supplementary Fig. 21). TEM images of the used Au/TiO_2@M-N-800 catalyst exhibited that the particle size of Au NPs remained unchanged. HRTEM images demonstrated that the Au NPs were still encapsulated by TiO_x overlayer after the reaction, leading to unstable Au nanoparticles (Figure R11 or Supplementary Fig. 22). We have added these new data in the revised manuscript and supplementary information.

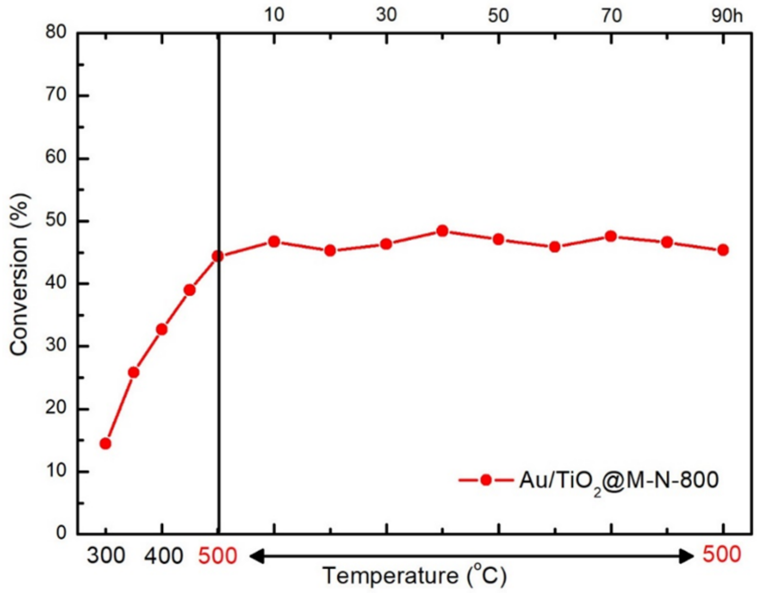

Figure R10. Water-gas shift (WGS) reaction on Au/TiO_2@M-N-800 nanocatalyst. Long-term WGS reaction at 500 ^oC on Au/TiO_2@M-N-800 with a feed gas comprising 2 vol% CO/ 10 vol% H_2O/ 88 vol% He at 30 mL min^{-1}, 0.1 g catalyst, space velocity (SV) = 18 L g_{cat}^{-1} h^{-1}.

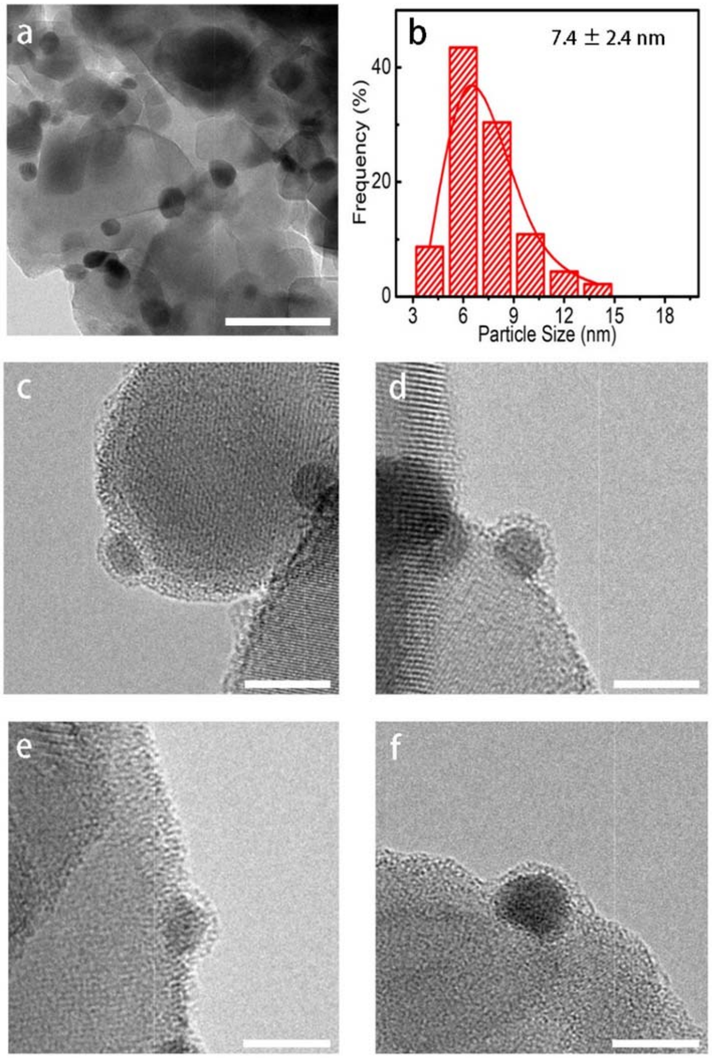

Figure R11. Electroscopy microscpoy images of Au/TiO_2@M-N-800. a TEM images of Au/TiO_2@M-N-800 after WGS reaction at 500 ^oC for 90 h. **b** Particle size distribution of Au/TiO_2@M-N-800 after WGS reaction at 500 ^oC for 90 h. **c, d, e** and **f** HRTEM images of Au/TiO_2@M-N-800 after WGS reaction at 500 ^oC for 90 h, in which Au NPs were still covered by TiO_x overlayer. The scale bar in **a** corresponds to 50 nm, and in **c**, **d**, **e** and **f** corresponds to 5 nm.

12 • The authors write on line 144-145 that the encapsulation state leads to a structural rearrangement of the TiO2 support in the neighbourhood of the metal particle. This is quite vague so can the authors elaborate on what they mean by this? Does it refer to the support 'beneath' the nanoparticle or the support that has encapsulated it? How big is the 'neighbourhood'?

Response: Thanks for your nice comments and good questions/suggestions. Actually, the reorganization of the support in the neighborhood of the metal particle referred to the support that has encapsulated the Au NPs in which Au-Ti bond was formed, which was similar to the classical SMSI behavior of Pt/TiO_2 and Rh/TiO_2 where Pt-Ti and Rh-Ti bond were observed (J. Phys. Chem 1986, 90, 6811-4817 and J. Phys. Chem 1986, 90, 1733-1736). While for the support

beneath the nanoparticle may remained unchanged. Therefore, this rearrangement of the support only generated where there was Au NPs and encapsulation occurred. Otherwise, no rearrangement would occur. We have accordingly added this to the revised manuscript.

13 • Can the authors comment on why the activity of the catalysts appeared to increase over time?

Response: This is a good question. Actually, we are also interested in the increase of the activity over time at 400 ^{o}C because there was almost no change in the particle size of Au NPs during the reaction. However, it was observed that the overlayer of the used catalyst was not so dense compared with the fresh catalyst (Please see Figure R12 or Supplementary Fig. 19 and Fig. 23), suggesting much more active site was accessible, and therefore the activity increased over reaction time. These aspects have been discussed in the revised manuscript and supplementary information.

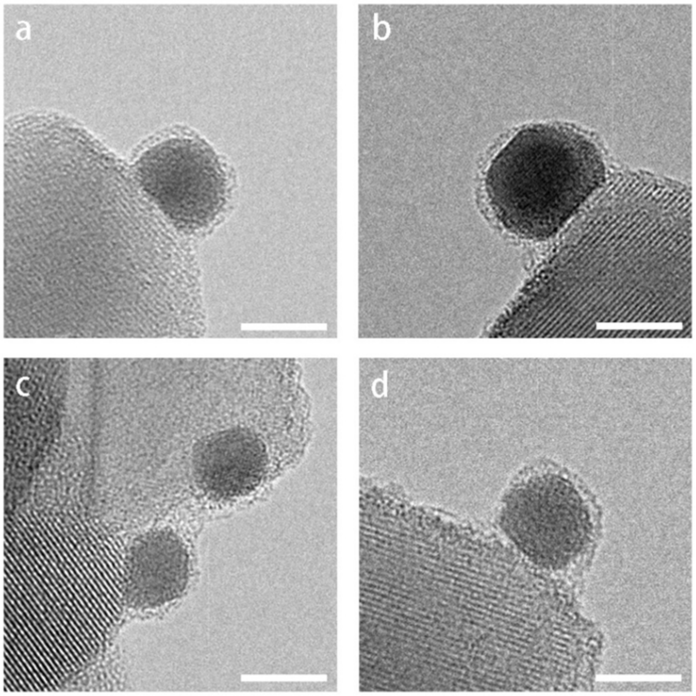

Figure R12. HRTEM analysis of Au/TiO_2@M-N-800. a and **b** HRTEM images of Au/TiO_2@M-N-800 after catalyzing CO oxidation at 400 ^{o}C for 100 h, in which the TiO_x overlayer was not dense as that of the fresh catalyst. **c** and **d** HRTEM images of Au/TiO_2@M-N-800 after simulated CO emission control reaction at 400 ^{o}C for 10 day, in which the TiO_x overlayer was not dense as that of the fresh catalyst. The scale bars are all 5 nm.

14 • Can the authors comment on a) what percentage of Au NPs were encapsulated and b) if the smaller Au NPs were equally as likely as the larger ones to be encapsulated?

Response: (a) From the HRTEM results, for the Au NPs particle size less than 12 nm which were all encapsulated. Therefore, it was calculated that more than 90 % of Au NPs was encapsulated on the basis of the particle size distribution (Supplementary Fig. 1h). We have added this information in the revised manuscript.

(b) Yes, the smaller Au NPs can also be encapsulated similarly to that of the larger ones (Please see Figure R1 or Supplementary Fig. 3 & Fig. 22). However, it is difficult to observe the morphology and encapsulation on Au when the Au NPs particle size is less than 2 nm. And we have added these data to the revised manuscript.

15 In conclusion, the manuscript reports interesting results with the potential to influence thinking in the field of heterogeneous catalysis - if they can demonstrate that this method of encapsulation is superior to alternative techniques.
Response: Thanks again for your professional comments and helpful suggestions. We have followed these comments and suggestions to improve our revised manuscript.

References
[1]. Classical strong metal–support interactions between gold nanoparticles and titanium dioxide" Tang et al., Sci. Adv. 2017; 3: e1700231.
[2] Goodman, Catalysis by supported gold nanoclusters in Dekker Encyclopedia of Nanoscience and Nanotechnology, Pages 611-620.

Reviewer #2 (Remarks to the Author):

Wang and co-workers reported the construction of SMSI on the as-prepared Au/TiO2 catalysts using a melamine induced TiOx layer under the calcination treatment. The formed TiOx layer could stabilize the Au nanoparticles from sintering in the oxidation of CO, which is used here as a model reaction. The results are interesting and reasonable. In addition, the method was extendable, which can be used for the formation of SMSI on the commercial catalyst. This manuscript might be acceptable for publication in nat. comm., but major revision is still necessary to address the following issues.
Response: Thanks very much for your professional comments.

1. In the classical SMSI from the high-temperature reduction treatment, a de-SMSI usually occurred in the water-treatment or oxidative conditions, where the oxide layer was removed under the given conditions. However, this work emphasized that the layer was stable even after the high-temperature calcination, and the reaction conditions with water feed. What is the reason for the oxide layer stabilization?
Response: This is a good question. Actually, we are also interested in this phenomenon and have been working for this topic for a while trying to figure out the reasons and mechanism. But unfortunately, it is very difficult to answer at present. What should be noted is that the encapsulation in this work occurs under oxidative atmosphere, which implies that the TiO_x layer formed is endurable under oxidative atmosphere at high temperature. Recently, Xiao et al. constructed TiO_x overlayer on Au NPs via wet-chemistry strategy, in which the overlayer still existed after the reaction at 300 ^oC for 100 h (J. Am. Chem. Soc. 2019, 141, 2975-2983). Therefore, the formation condition of the encapsulation may determine the property of TiO_x overlayer.

2. The TOF of the Au catalysts have been provided, it is obtained on the basis of accessible Au? A comparison with the other Au catalysts on the catalytic activity should be addressed, for example, comparison with the Au/TiO2 with wet-chemistry SMSI (Wang and Xiao, JACS), Au/HAP with oxidative SMSI (Wang and Zhang, JACS), Au/TiO2 with classical SMSI (Qiao and Wang, Sci Adv).

Response: Thank you for your suggestion. The TOF of Au catalysts was measured at 25 ^{o}C and calculated on the basis of the amount of exposed Au sites (mol of transformed CO per mol of accessible Au site per hour). And we have added the catalytic activity of Au/TiO_2 with wet-chemistry SMSI (Wang and Xiao, J. Am. Chem. Soc. 2019, 141, 2975-2983), Au/HAP with oxidative SMSI (Wang and Zhang, J. Am. Chem. Soc. 2016, 138, 56-59), Au/TiO_2 with classical SMSI (Qiao and Wang, Sci Adv 2017, 3, e1700231) in Table S3. And we have added this discussion into the revised manuscript and supplementary information.

3. More evidences should be provided on the formation of SMSI. The author talked about the formation of electronic interaction between Au and TiOx led by the melamine. More details should be provided. Is the melamine completely burned after the calcination? The melamine might be continuous decomposed during the calcination treatment at higher temperature. Does this molecule work at low temperature (because it is decomposed at high temperature)? The composition of the oxide layer, pure TiOx or carbon-containing TiOx, should be studied.

Response: We appreciate the reviewer's question and suggestion. To demonstrate it, we have performed blank test in which melamine was pretreated at 600 ^{o}C in N_2 atmosphere and further calcination at 800 ^{o}C in air and nothing existed in the quartz boat after calcination in air, which meant melamine has decomposed completely in this condition. On the other hand, no N1s XPS signal in Au/TiO_2@M-N-800 was observed which definitely demonstrated melamine had decomposed completely (Supplementary Fig. 9). Moreover, we have showed that when catalysts was heated at relatively low temperature, for example at 500 or 600 ^{o}C (denoted as Au/TiO_2@M-N-500 and Au/TiO_2@M-N-600), no encapsulation was observed (Supplementary Fig. 31-32), implying melamine only works at high temperature. However, the reasons of this phenomenon still need further investigation. To identify whether there was C or N in the overlayer, electron energy loss spectroscopy (EELS) has been carried out once more. As shown in Figure R8 and R9 (or Supplementary Fig. 7 and Fig. 8) that oxide layer was composed of Ti and O and no signal of C or N was observed, suggesting that the composition of overlayer was pure TiO_x. On the other hand, it is difficult to identify C species with scanning transmission electron microscopy-energy-dispersive X-ray spectroscopy (STEM-EDX) because of great inaccuracy. And we have added these data with corresponding discussion on to the revised manuscript and supplementary information.

4. The RESULTS section started with the characterization data. A brief introduction of the synthesis procedures should benefit the readers to understand the process.

Response: Thanks very much for your professional and helpful suggestion. We have added the synthesis procedures of the catalysts to the revised manuscript as "The catalysts were prepared with deposition-precipitation (DP) method and followed by modification with melamine and pretreatment at 600 ^{o}C in N_2 atmosphere and further calcination at 800 ^{o}C in air."

5. Some other important references on the sinter-resistant catalysts should be added in the

revised manuscript, such as Nature Catal. 2018, 1, 540-546; Science, 2012, 335, 1205; Angew, 2012, 51, 5929; 2017, 56, 9747-9751.

Response: Thank you for your suggestion. We are sorry for missing these important references. We have cited these references in the corresponding section in the revised manuscript.

REVIEWERS' COMMENTS:

Reviewer #1 (Remarks to the Author):

The authors listened carefully to the concerns previously raised and have included additional data to support their claims, which are now well-substantiated.

One suggestion regarding the Au 4f XPS data: After reconsideration, the authors decided to remove the data entirely as no trends were observed. Although this is acceptable, it may be worthwhile leaving the data in the supplementary information and stating that no significant changes in the binding energy were observed. This is simply because XPS is almost always presented for such catalysts and the reader would likely be interested to know if any changes were seen. Additionally, the absence of any changes underlines that the melamine treatment and subsequent calcinations do not impact the speciation/oxidation state of the Au.

Overall, the improved manuscript is now suitable for publication. The findings represent an interesting and important step forward in the design of stable supported nanoparticles and will more than likely influence thinking in the field.

Reviewer #2 (Remarks to the Author):

Appropriate responses have been made to my questions, and the corresponding modification was included in the manuscript. I recommend the publication in Nat. Commun.

REVIEWERS' COMMENTS:

Reviewer #1 (Remarks to the Author):

The authors listened carefully to the concerns previously raised and have included additional data to support their claims, which are now well-substantiated.

One suggestion regarding the Au 4f XPS data: After reconsideration, the authors decided to remove the data entirely as no trends were observed. Although this is acceptable, it may be worthwhile leaving the data in the supplementary information and stating that no significant changes in the binding energy were observed. This is simply because XPS is almost always presented for such catalysts and the reader would likely be interested to know if any changes were seen. Additionally, the absence of any changes underlines that the melamine treatment and subsequent calcinations do not impact the speciation/oxidation state of the Au.

Overall, the improved manuscript is now suitable for publication. The findings represent an interesting and important step forward in the design of stable supported nanoparticles and will more than likely influence thinking in the field.

Response: Thanks very much for your nice comment! As suggested, Au 4*f* XPS spectra have been added to the supplementary information and we have added the stating in the revised manuscript as "While no obvious changes in the binding energy of Au 4*f* were observed, suggesting that melamine treatment and subsequent calcinations did not affect the oxidation state of the surface Au significantly (Supplementary Fig. 12)."

Reviewer #2 (Remarks to the Author):

Appropriate responses have been made to my questions, and the corresponding modification was included in the manuscript. I recommend the publication in Nat. Commun.

Response: Thanks very much for your nice comment!
